# Solvent‐Free Fabrication Methods of Metal Halide Perovskites

**DOI:** 10.1002/adma.202416604

**Published:** 2025-08-21

**Authors:** Ran Ji, Arindam Mallick, Boris Rivkin, Hurriyet Yuce‐Cakir, Oana D. Jurchescu, Yana Vaynzof

**Affiliations:** ^1^ Chair for Emerging Electronic Technologies Technical University of Dresden Nöthnitzer Str. 61 01187 Dresden Germany; ^2^ Leibniz‐Institute for Solid State and Materials Research Dresden Helmholtzstraße 20 01069 Dresden Germany; ^3^ Department of Physics and Center for Functional Materials Wake Forest University Winston‐Salem NC 27109 USA

**Keywords:** metal halide perovskites, scalable fabrication, solvent‐free deposition

## Abstract

Metal halide perovskites have ascended as a remarkable class of materials in recent years, demonstrating exceptional promise for application in various electronic and optoelectronic devices. The vast majority of research on these materials focuses on their processing from solution, which is relatively easily executed in laboratory settings, but its scalability for industrial mass production remains a significant hurdle. Furthermore, its reliance on highly toxic solvents imposes limitations with respect to large‐area fabrication and have a negative environmental impact. This review comprehensively explores the current status of solvent‐free fabrication methods for metal halide perovskites, outlines the current challenges and opportunities, and provides a critical assessment of the technological readiness and future research directions. The development of robust and scalable solvent‐free fabrication methodologies is essential to realizing the full potential of metal halide perovskites. We hope that this review will serve as a catalyst, inspiring and guiding researchers to explore new strategies for the solvent‐free deposition of these remarkable materials, thereby expediting their integration into technological applications.

## Graphical abstract

This review explores the current status of solvent‐free fabrication methods of metal halide perovskites, such as thermal evaporation, magnetron sputtering, pulsed laser deposition, e‐beam and atomic layer deposition, solid phase growth, and laser printing. It outlines the current state of knowledge and discusses key challenges and future research directions.

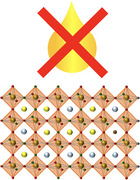

## 1 Introduction

Metal halide perovskites (MHPs) have attracted enormous attention as an emerging class of semiconductors with highly desirable and extremely versatile properties. Remarkably, they can be easily processed at ambient or close to ambient temperatures, yet they exhibit high luminescence efficiency and allow degree of energetic disorder, comparable to traditional inorganic semiconductors like GaAs,^[^ ^1^ ^]^ which require a much more demanding processing. Other favorable properties of perovskite semiconductors include high absorption coefficients,^[^ ^2^ ^]^ low exciton binding energy,^[^ ^3^ ^]^ and high charge carrier mobilities.^[^ ^4^ ^]^ This unique combination makes perovskite materials exceptionally attractive for application in a wide range of optoelectronic devices, such as photovoltaic and light‐emitting diodes (LEDs), photonic devices and lasers, field‐effect transistors (FETs), and memory devices.

The rapid progress in perovskite photovoltaic devices is awe‐inspiring: since their first introduction in 2009,^[^ ^5^ ^]^ the power conversion efficiency (PCE) of these devices increased nearly sixfold, surpassing 26%.^[^ ^6^ ^]^ Theoretical calculations suggest that efficiencies up to 30% are possible with further optimization, making perovskite solar cells (PSCs) one of the most promising technologies among third‐generation photovoltaic devices.^[^ ^7^ ^]^ Similarly, perovskite LEDs have advanced rapidly since their first demonstration in 2014.^[^ ^8^, ^9^ ^]^ Recent breakthroughs also enabled the use of perovskites in electronics, with impressive performance of FETs based on three‐ and two‐dimensional perovskite materials.^[^ ^10^, ^11^ ^]^

Particular interest has been dedicated to the solution‐processing of MHPs, leading to an extensive study of their crystallization dynamics,^[^ ^12^, ^13^ ^]^ solvent and additive engineering.^[^ ^14^, ^15^ ^]^ The main focus on processing from solution has been fueled by several contributing factors. First, it is easily accessible in laboratory settings using a spin‐coater, a widely available, low‐cost deposition tool. Since laboratory‐scale samples are typically of small size (≈1 cm^2^), spin‐coating is highly suitable for rapidly processing thin films of this size. Secondly, solution processing offers a range of possibilities for controlling and manipulating the properties of perovskite films. Particularly noteworthy is the control over film formation established by solvent and additive engineering, which proved indispensable in achieving high device performance.^[^ ^16^, ^17^ ^]^ We and others have demonstrated that solvent engineering and additives can be employed to tune the grain size,^[^ ^18^, ^19^ ^]^ orientation, and phase of the MHPs, and lead to defect passivation, improved stability, and elimination of hysteresis.^[^ ^20^, ^21^ ^]^

While the processing of MHPs from solution is simple and accessible in a laboratory setting, it is not easily transferable to large‐scale manufacturing and mass production of large‐area devices required for industrial applications. First of all, spin coating is not applicable to photovoltaic module‐sized devices (≈1–2 m^2^) due to the limitations on maximum sample size (typically less than 18 inches). Additionally, about 90% of the precursor ink is spun off during deposition, making it wasteful and increasing the overall fabrication costs. Notably, much of the knowledge and expertise developed for optimizing spin‐cast devices is not directly applicable to solution‐based large‐area fabrication techniques such as printing or slot die coating.^[^ ^22^ ^]^ For example, while the use of antisolvents to trigger the crystallization of spin‐cast perovskite layers is ubiquitous and widely optimized,^[^ ^23^ ^]^ this strategy is not transferable to their large area processing due to the significant volumes of antisolvents needed and the difficulty of doing so uniformly across large areas. Another limitation stems from the fact that solution processing of MHPs is often performed by utilizing highly toxic solvents, such as dimethylformamide (DMF). In small‐scale settings such as laboratory research, these solvents can be used safely by applying appropriate handling and safety precautions. However, the mass production of large‐area perovskite‐based devices cannot rely on toxic solvents that are carcinogenic and miscible in water, hence posing the risk of contamination of drinking water and increasing health concerns for the personnel involved.^[^ ^24^ ^]^

These serious challenges have inspired increasing interest in developing solvent‐free deposition methods for MHPs. The proposed techniques range from industry standards, such as thermal evaporation (TE) or sputtering, to unconventional methods like laser printing. This review provides a comprehensive overview of solvent‐free fabrication methods for thin perovskite films, critically discussing their advantages and disadvantages and offering an assessment of the current state of the art. Section 2 is devoted to vacuum‐based deposition techniques such as TE, magnetron sputtering, chemical vapor deposition (CVD), atomic layer deposition (ALD), pulsed laser deposition (PLD), and electron beam evaporation. Next, solvent‐free printing and melt‐based techniques are discussed in Sections 3 and 4, respectively. Section 5 provides a brief overview of solvent‐free fabrication techniques for MHP as bulk or powders through vertical Bridgman growth and mechano‐synthesis, respectively, with special attention to their complementary capacity in preparing source material for other deposition techniques. The review concludes with a critical analysis of the challenges that remain to be addressed and a proposal for future research directions (Section 6), which we hope will motivate the readers to develop new strategies for solvent‐free deposition of MHPs, thus expediting their integration into industrial applications.

## 2 Vacuum Deposition Techniques

The deposition of MHPs can be achieved through a diverse array of vacuum‐based methods. Among these, the most widely explored method is based on their deposition from vapor, a process known as TE. The seminal work by Snaith and co‐workers reporting on the first thermally deposited PSCs in 2013 was one of the first indicators that this method is a strong contender to supersede the solution‐processing of perovskites.^[^ ^25^ ^]^ More recently, other methods such as magnetron sputtering, CVD, ALD, and PLD have also made significant progress. This section provides an overview of these methods and describes the current state of knowledge regarding their application to the deposition of MHPs. We first discuss the crystal growth dynamics of MHP during vapor processing. Then, we introduce the principles of various deposition methods and their progress in the field of perovskite (opto) electronic devices.

### 2.1 Crystal Growth Dynamics of Vapor‐Based Processing Methods

The remarkable progress in developing high‐quality MHP films has been significantly driven by advancements in crystallization control. Over the past decade, substantial insights into perovskite formation mechanisms have been established. However, most crystallization strategies have been developed for solution‐processed systems, and a systematic understanding of crystallization dynamics in solvent‐free fabrication methods remains lacking. Fortunately, despite differing crystallization environments, mechanisms derived from solution‐based approaches can partially inform the analysis of dry‐processed systems. For the widely studied ABX_3_ perovskites, the A‐site is occupied by organic or inorganic cations (e.g., methylammonium [MA^+^], formamidinium [FA^+^], or cesium [Cs^+^]), the B‐site by metal cations (Pb^2^^+^ or Sn^2^^+^), and the X‐site by halide anions (Cl^−^, Br^−^, or I^−^).^[^ ^26^ ^]^ These components assemble into ordered networks during fabrication, where corner‐sharing [BX_6_]^4−^ octahedra form tetragonal or cubic frameworks stabilized by A‐site cations occupying interstitial voids.^[^ ^27^ ^]^ The formation of perovskite networks proceeds through sequential stages, starting with the emergence of precursor particles.

In solvent‐free methods such as PLD or magnetron sputtering, the initial precursor dissociates and generates colloidal particles, hypothesized to involve soft coordination complexes between organic/inorganic components via lead polyhalide frameworks.^[^ ^28^ ^]^ This process, critical for nucleation and growth in solution‐based systems, has been extensively characterized through absorption spectroscopy and dynamic light scattering, revealing colloidal sizes ranging from nanometers to micrometers.^[^ ^28^, ^29^ ^]^ Their size is influenced by precursor concentration, organic/inorganic ratios,^[^ ^30^ ^]^ additives,^[^ ^30^ ^−^ ^33^ ^]^ acid‐base equilibria,^[^ ^34^, ^35^ ^]^ etc. In contrast, solvent‐free processes—conducted under vacuum or gaseous environments—lack direct characterization tools for colloidal components, as colloids form via physical dispersion (e.g., thermal ablation, particle bombardment, or laser irradiation) rather than solvent‐mediated coordination. It is postulated that external energy input (e.g., laser fluence) may modulate colloid dissociation, thereby regulating particle size.

The subsequent step is the nucleation process. In solution environment, nucleation involves solute precipitation from a supersaturated solution. Similarly, nucleation in solvent‐free processes involves interfacial energy competition among the substrate, condensing material, and vapor phase. The minimum‐energy shape of a nucleus resembles a cap. The critical size of the nucleus depends on the deposition rate and the precursor sticking coefficients. The large nuclei create isolated patches (islands) on the substrates. After nucleation, crystal growth becomes a non‐uniform process influenced by precursor supply, ionic/hydrogen bonding interactions, and competition between nucleation and growth caused by mass transport and heat transport. The crystalline film growth depends on the surface mobility of the “adatom” (vapor atoms). Typically, the adatom diffuses through several atomic distances before sticking to a stable position within the newly formed film. The surface temperature of the substrate determines the adatom's surface diffusion ability. High temperatures favor rapid and defect‐free crystal growth, whereas low‐temperature crystal growth may be besieged by energetic particle impingement that results in disordered or amorphous structures.

**Figure 1:**
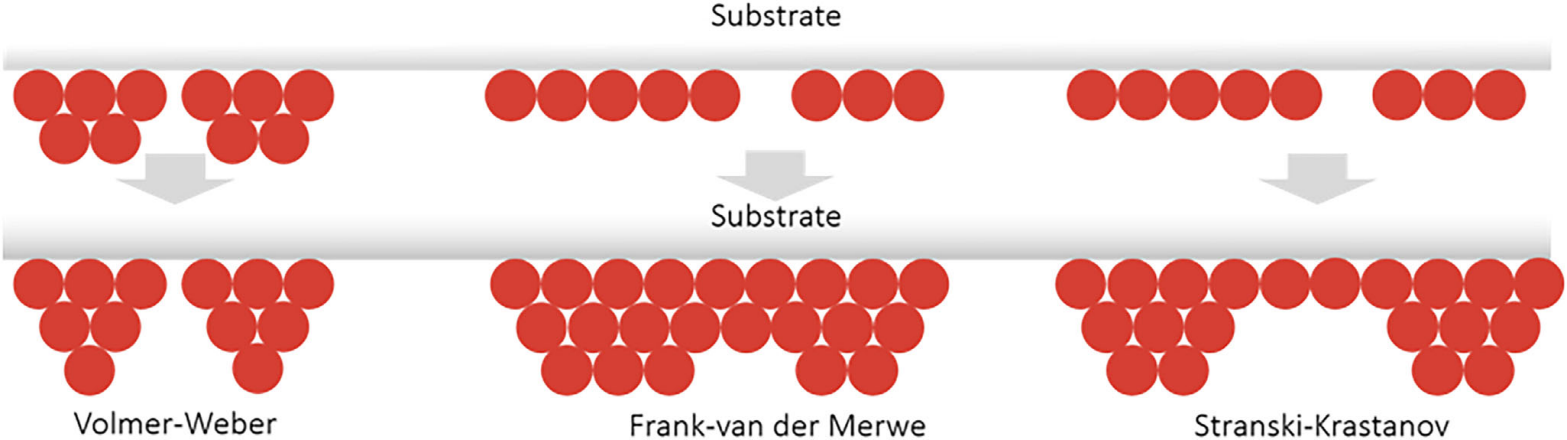
Illustration of the film formation mechanisms.^[^ ^36^ ^]^ Reproduced with permission. Copyright 2021, Wiley‐VCH.

In general, the crystal‐growth process of vapor deposition is mainly governed by three growth modes: the Volmer–Weber mode (island growth), Frank–van der Merwe mode (layer growth), and Stranski–Krastanov mode (layer‐island hybrid growth) as illustrated in **Figure** **1**. In the Volmer–Weber mode, deposited atoms or molecules nucleate directly on the substrate surface and grow into islands. Subsequently, the islands grow and eventually merge to form a continuous polycrystalline film. This mode is favored when the adsorbate–adsorbate bonding is much stronger than adsorbate–surface interactions. On the other hand, layer growth occurs when the atoms are more strongly bound to the substrate than to each other, which is typically observed in epitaxial systems. In such a case, a complete monolayer forms first on the surface of the substrate and is then covered with a less tightly bound second layer. In a layer‐island growth mode, the adsorbate–surface and adsorbate–adsorbate interactions are balanced. When the lattice of the substrate is generally not matched with that of the perovskite layer or even its precursors, the binding force between the vaporized atoms or molecules and the substrate is not strong, and the perovskite film growth mainly follows the Volmer‐Weber mode.^[^ ^36^, ^37^ ^]^ The resulting islands (or initial nucleation sites) then grow into grains and determine the microstructure of the layer. Intuitively, the nuclei density should increase with the condensation rate, suggesting a strong link between the deposition parameters and the film microstructure. Another critical factor is that different perovskite precursors stick differently to the substrate. Specifically, it is generally considered that perovskite growth during co‐evaporation occurs via two steps: 1) metal halides preferentially stick to the substrate, followed by 2) conversion to a perovskite by organic halides or cesium halides. This makes it necessary to consider the differential sticking coefficients of the precursors when developing strategies for microstructural control of vapor‐deposited perovskite layers. Meorover, since the formation of the perovskite network is an exothermic reaction (e.g., −0.1 eV for MAPbI_3_)^[^ ^38^, ^39^ ^]^ and the activation energy is low (e.g., 0.70–1.0 eV for MAPbI_3_),^[^ ^40^, ^41^ ^]^ the perovskites can crystallize rapidly, causing uncontrollable film morphology. Slow crystal growth is essential in the formation of high‐quality perovskite layers. Specific strategies for improving crystallization will be detailed in subsequent sections and organized by fabrication methodology.

### 2.2 Thermal Evaporation

Research efforts invested in MHPs’ TE have grown rapidly, motivated by many advantages of TE, such as precise control of deposition conditions and rates across various perovskite compositions, high reproducibility in resulting film thicknesses, excellent film uniformity over large areas, complete and conformal substrate coverage, minimum material waste, and amenability to a wide range of substrates, from transparent conductive oxide‐coated glass to flexible metal or plastic foils, and textured silicon. TE is amongst the most straightforward techniques for upscaling, easing the future integration of perovskites into existing industrial manufacturing lines. In this section, we will introduce various methods for TE of perovskites, describe the mechanism for film formation, and discuss the impact of various deposition parameters on film properties. We also address the influence of substrate choice and the current landscape in developing material engineering approaches for TE, including the use of additives and post‐treatment.

#### 2.2.1 Methods for the Deposition of MHPs by TE

**Figure 2:**
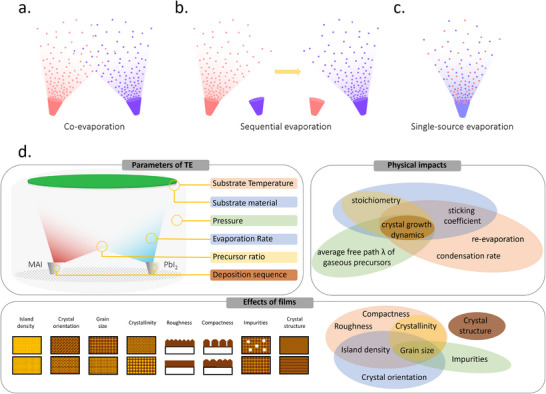
Schematic diagram of a) co‐evaporation, b) sequential evaporation, and c) single‐source evaporation. d) Key parameters in TE and their impact on perovskite thin film growth.

Three different methodologies can be applied to the formation of MHPs by TE: co‐evaporation, sequential evaporation, and single‐source evaporation. Co‐evaporation, as shown in **Figure** **2**a, was reported to produce high‐quality perovskite layers.^[^ ^42^ ^−^ ^46^ ^]^ In this technique, each precursor is placed into a separate crucible inside the evaporation chamber. The crucibles are simultaneously heated at independently controlled temperatures appropriate to sublime each precursor. The individual deposition rates are monitored using quartz crystal microbalances (QCMs) to control the stoichiometry of the deposited film precisely. The concurrent evaporation of all precursors facilitates a reaction in close proximity to, or directly on the substrate, enabling the formation of the desired perovskite structure. While often proven effective, co‐evaporation has an inherent disadvantage, as it precludes the independent optimization of the deposition conditions (temperature and partial pressure) for each of the precursors, which can be particularly challenging for organo‐halide compounds. Co‐evaporation may also suffer from poor reproducibility caused by the crosstalk of the QCMs during the evaporation of organic halides.^[^ ^47^, ^48^ ^]^ Sequential evaporation, illustrated in Figure 2b, was developed to mitigate these drawbacks. In its simplest form, sequential TE is a division of co‐evaporation into two or more deposition steps, with one precursor deposited after the other. This separation enables the independent control of the deposition conditions for each precursor to improve the MHP film quality.

While co‐evaporation and sequential evaporation remain the most popular, both methods require a regular calibration of the deposition rates and precursor ratios to obtain high‐quality films with the desired stoichiometry. This can be particularly challenging for organic‐inorganic hybrid halide perovskites due to the irregular and often unpredictable evaporation characteristics of the organic halide precursor. To circumvent this problem, single‐source TE—shown in Figure 2c—has been proposed. This technique utilizes a pre‐synthesized target material in the form of a powder or a crystal, which is evaporated from a single crucible onto the substrate, ideally without any chemical reaction. This approach aims to simplify the process by eliminating the need for in situ chemical reactions and intricate stoichiometry control during deposition. In comparison with the former two methods, single source evaporation is less widely investigated, but still showed success in conjunction with a wide range of perovskite material systems, such as CH_3_NH_3_PbI_3_ (MAPbI_3_),^[^ ^49^ ^−^ ^52^ ^]^ CH(NH_2_)_2_PbI_3_ (FAPbI_3_),^[^ ^53^ ^]^ CsPbBr_3_,^[^ ^54^ ^]^ 2D BA_2_MA_3_Pb_4_I_13_,^[^ ^55^, ^56^ ^]^ Cs_2_AgBiBr_6_,^[^ ^57^ ^]^ CsBi_3_I_10_,^[^ ^58^ ^]^ CsGe_0.5_Sn_0.5_I_3_,^[^ ^59^ ^]^ and CsGeI_3_.^[^ ^60^ ^]^ An essential branch in single‐source evaporation is flash evaporation, by which the temperature of the crucible is increased rapidly, enabling the ultra‐fast sublimation of the perovskite precursor within seconds. This high‐speed evaporation, intended to prompt the perovskite to be vaporized directly, may also inadvertently decompose it back to its precursors. Despite this drawback, flash evaporation offers significant advantages in high‐throughput fabrication in industrial production compared to the more time‐consuming sequential or co‐evaporation methods.

#### 2.2.2 Mechanism of Film Formation by TE

In general, the TE process consists of three steps: i) precursors vaporization: in this step, the precursors are heated to their sublimation point, causing a large number of particles to transition into the gaseous phase and leave the surface of the solid precursors; ii) vapor transport: the low pressure in the chamber offers a sufficiently large mean free path so that the gaseous precursors can propagate from the source to the substrate with a negligible number of collisions; iii) precursor deposition and film formation: the precursor particles condense on the substrate, nucleate and grow into a film.

The properties of the film resulting from the TE process are influenced by many parameters, including the evaporation rate, background pressure, substrate, and temperature, which influence the nucleation and growth process (Figure 2d). These parameters are under extensive investigation and optimization to develop protocols for forming dense, continuous, and uniform perovskite films, which is a key prerequisite for high‐performance optoelectronic devices. Therefore, understanding the principles of the evaporation process and the various influencing factors during the film deposition is fundamental to the success of this technology design and control. The following section discusses the strategies and progress of various TE‐based methodologies developed for high‐quality perovskite films.

#### 2.2.3 Compositional Tuning

Methylammonium (CH_3_NH_3_^+^, MA^+^)‐based perovskite compositions became the preferred materials in the early days of solution‐processed perovskite research. This is due to a combination of excellent properties: high absorption coefficient, the 1.55 eV bandgap that is well‐suited for solar energy conversion, and long carrier diffusion lengths. These characteristics made MAPbI_3_ the benchmark for solution‐processed materials. The studies of TE perovskites followed the same route and focused on the same material system. In 2013, Liu et al. used co‐evaporation to fabricate the first TE‐processed MAPbI_3‐x_Cl_x_.^[^ ^24^ ^]^ They co‐evaporated MAI and PbCl_2_ precursors, obtaining uniform and flat films of MAPbI_3−x_Cl_x_. The resulting perovskite films exhibited similar crystal properties, better film coverage, and higher uniformity compared to their spin‐coated counterparts. Soon after, Malinkiewicz et al. showed that MAPbI_3_ could be obtained by co‐evaporation of MAI and PbI_2_.^[^ ^61^ ^]^ These works introduced TE more generally as a viable method to fabricate high‐quality perovskite films and sparked interest in future investigations.

**Figure 3:**
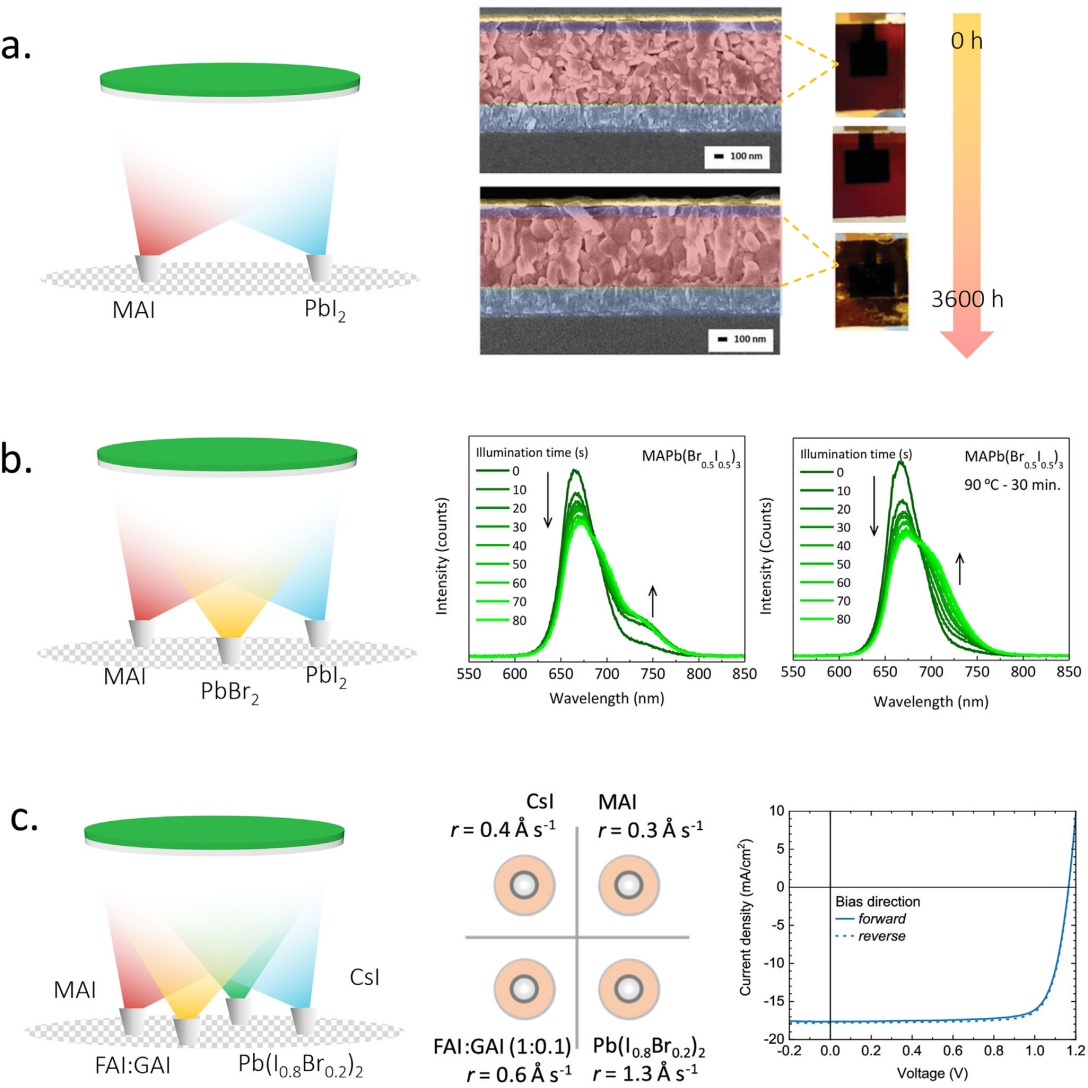
a) Left: Schematic diagram of double‐source co‐evaporation process. Right: photographs taken from the glass side and cross‐sectional SEM images of fresh and thermal aging at 85 °C and 10% relative humidity (RH) MAPbI_3_ solar cell devices. Reproduced with permission. Copyright 2021, Wiley‐VCH.^[^ ^62^ ^]^ b) Left: Schematic diagram of triple‐source co‐evaporation process. Middle and right: UV–vis spectra of MAPbI_x_Br_3‐x_ aging under illumination at room temperature and 90 °C for 30 min. Reproduced with permission. Copyright 2017, American Chemical Society.^[^ ^66^ ^]^ c) Left and middle: schematics of quadro‐source co‐evaporation and the deposition sources layout. Right: *J*–*V* curve of the quadruple‐cation double halide PSC. Reproduced under the Creative Commons license.^[^ ^72^ ^]^

While excess MAI is generally considered to be responsible for the poor thermal stability of solution‐processed perovskites, this does not seem to be the case when the perovskite films are deposited using TE. Dewi et al. found that pure TE MAPbI_3_ perovskites are considerably more stable than their solution‐processed counterparts and those based on multi‐cation formulations (**Figure** **3**a).^[^ ^62^ ^]^ This observation led the authors to hypothesize that the excellent TE MAPbI_3_ thermal stability is related to the inherent strain‐stress‐free and compact TE film. Further studies have confirmed the remarkable stability of TE MA perovskite, withstanding continuous stress at 85 °C for thousands of hours.^[^ ^62^ ^−^ ^64^ ^]^

The composition tuning in TE MAPbI_3_ began with adjusting the ratio of the two precursor materials: MAI and PbI_2_. A study by Teuscher et al. highlighted the importance of precise stoichiometric control for obtaining uniform perovskite films with high optoelectronic quality. They observed that an excess of PbI_2_ resulted in unreacted PbI_2_ crystals on the film surface, whereas large pinholes in the perovskite film were observed when films were grown under an MAI‐rich condition.^[^ ^65^ ^]^

Inspired by the success of compositional engineering in solution‐processed perovskites, where mixed‐cation and mixed‐halide compositions exhibited higher stability and photovoltaic properties, researchers have extended this approach to TE. As such, instead of just two source crucibles, they now use multiple crucibles to achieve a wider range of compositions and ultimately meet the high standards of optoelectronic devices. For example, Longo et al. pioneered this approach by co‐evaporating MAI, PbBr_2_, and PbI_2_ to fabricate mixed‐halide perovskite compositions of MAPb(Br_0.2_I_0.8_)_3_ and MAPb(Br_0.5_I_0.5_)_3_ with bandgaps of 1.72 and 1.87 eV, respectively.^[^ ^66^ ^]^ However, halide segregation was observed at higher bromide concentrations (Figure 3b). This result highlighted the difficulty of obtaining stable perovskites simply by controlling halogen anions. In 2018, Gil‐Escrig et al. incorporated cesium into the mix by co‐evaporating MAI, CsBr, FAI, and PbI_2_.^[^ ^67^ ^]^ This was the first demonstration of TE triple‐cation perovskite with a Cs_0.5_FA_0.4_MA_0.1_Pb(Br_0.17_I_0.83_)_3_ composition. This complex perovskite showed promising light stability and photovoltaic performance. However, the more complicated perovskite composition also increased the complexity of the TE operation, requiring multiple reference experiments to determine and calibrate the evaporation rate of each precursor. These experiments rely on accurate knowledge of parameters like material density, acoustic impedance, and geometric parameters for each precursor and evaporation source, which can introduce variability and affect the reproducibility of the process. Therefore, finding a balance between compositional complexity and process simplicity is crucial for achieving reliable and reproducible fabrication of high‐quality perovskite films via TE.

Another shortcoming of MA‐based compositions is the notoriously challenging control of MAI TE. For instance, MAI evaporates isotropically upon heating and is not withheld by a crucible shutter; instead, it diffuses globally into the evaporation chamber. There, it substantially increases the chamber pressure and is often not precisely detected by standard deposition control techniques such as QCMs. This can lead to inaccurate deposition rate estimations by the QCM, thereby affecting the homogeneity and reproducibility of the films.^[^ ^68^ ^]^ This challenge motivated research efforts to focus on MA‐free TE perovskites.

In 2020, two groups independently reported the fabrication of MA‐free PSCs.^[^ ^69^, ^70^ ^]^ By co‐evaporating CsBr, PbI_2_, and FAI precursors, they achieved uniform films with promising photovoltaic performance, demonstrating the feasibility of high‐quality perovskite films without MAI. Furthermore, Gil‐Escrig et al. used PbBr_2_ and CsI to individually control the bromide and cesium content, allowing the deposition of perovskites with wide bandgaps between 1.7 and 1.8 eV.^[^ ^71^ ^]^ Li et al. simplified the process by mixing the metal halides with defined ratios to obtain a liquid‐phase molten salt, which can be formed at a much lower temperature than the melting point of each metal halide.^[^ ^44^ ^]^ More importantly, since it requires fewer crucibles to perform TE, this method allows for a reduced process complexity. Following the same approach, Susic et al. premixed both inorganic and organic components to obtain CsMAFA GA (Guanidinium) quadruple‐cation double halide perovskite films (Figure 3c), which, to the best of our knowledge, is the most complex perovskite composition currently achieved through TE. Currently, most TE facilities are equipped with four crucibles at most. Until chamber manufacturing technology is further improved, this precursor‐mixing method can help to improve the reproducibility and homogeneity of TE perovskites by simplifying the evaporation process.

**Figure 4:**
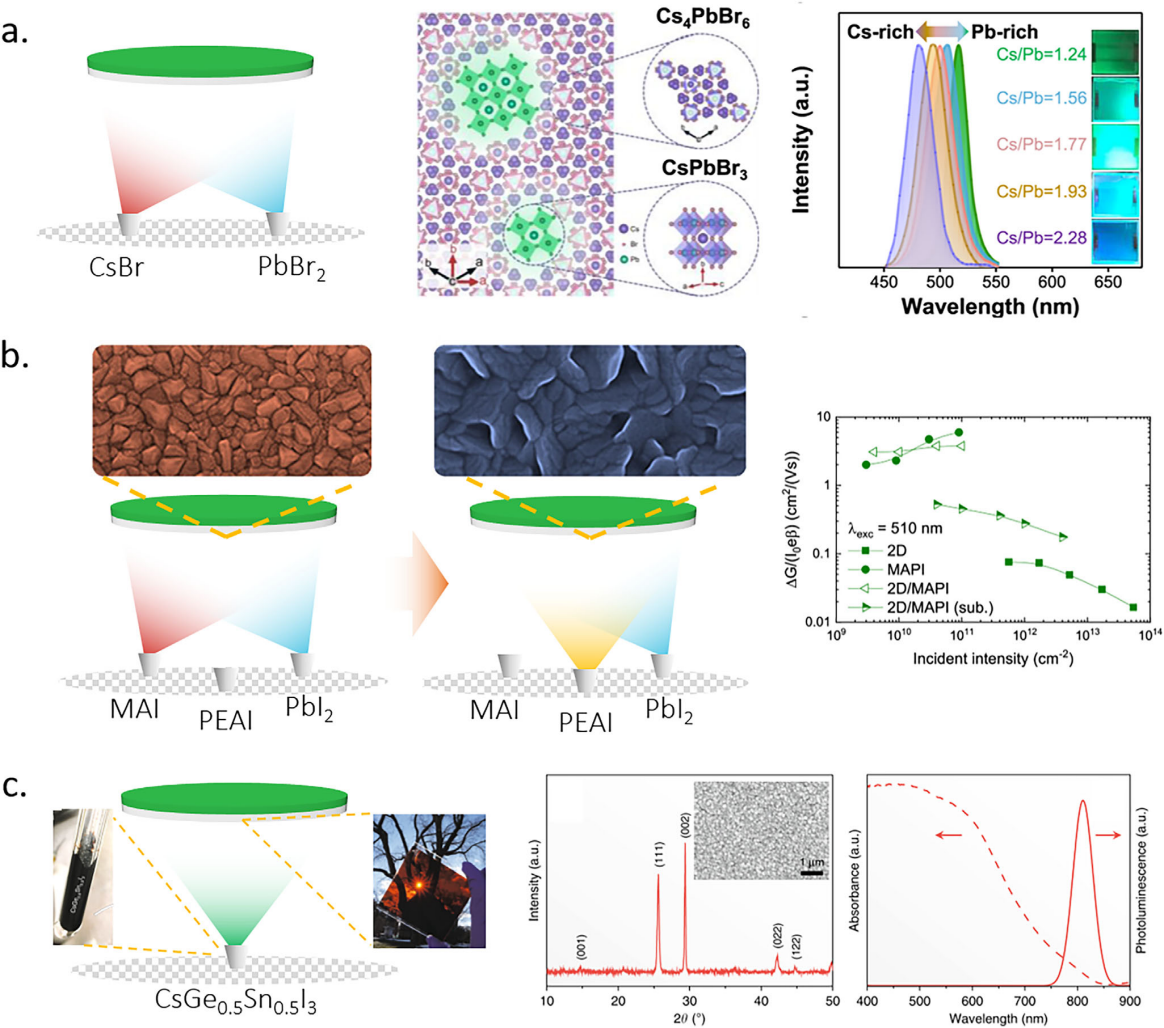
a) Left: Schematic diagram of double‐source co‐evaporation process. Middle: spatial relation between Cs_4_PbBr_6_ and CsPbBr_3_. Right: photoluminescence (PL) properties of CsPbBr_3_ with varied Cs/Pb ratio. Reproduced under the Creative Commons license.^[^ ^76^ ^]^ b) Left: Schematic of TE of 3D/2D perovskite heterojunction. Right: maximum time‐resolved microwave conductivity signal heights expressed in charge carrier yield times mobility for excitation at 510 nm. The annotation (sub.) denotes the light shining through the quartz substrate. Reproduced with permission. Copyright 2019, American Chemical Society.^[^ ^79^ ^]^ c) Left: Schematic diagram of single‐source TE of CsGe_0.5_Sn_0.5_I_3_ and photographs of precursor and perovskite film. Middle and right: XRD, SEM, UV–vis, PL spectrum of the CsGe_0.5_Sn_0.5_I_3_ film. Reproduced under the Creative Commons license.^[^ ^59^ ^]^

Since they lack the volatile organic component found in hybrid perovskites, inorganic perovskites, mainly CsPbX_3_ (X = Cl, Br, I), are expected to offer better reproducibility during TE. This is because the evaporation process for inorganic perovskites involves fewer components with more predictable behavior, leading to greater consistency in the deposited films. The first successful TE of an inorganic perovskite, specifically CsPbBr_2_I, was reported in early 2016.^[^ ^73^ ^]^ This composition exhibits a strong influence of precursor stoichiometry on the resulting film's properties. Research by Becker et al. revealed that an excess of CsI during CsPbI_3_ deposition favors the formation of the γ‐phase at a low temperature.^[^ ^74^ ^]^ In contrast, PbI_2_‐rich growth conditions lead to the formation of non‐perovskite δ‐phase. Huang et al. observed a similar trend and suggested that CsI‐rich growth conditions promote the generation of zero‐dimensional (0D) Cs_4_PbI_6_ that acts as a stabilizer for the γ‐CsPbI_3_ phase.^[^ ^75^ ^]^ Similarly, 0D Cs_4_PbBr_6_ was detected in CsPbBr_3_ processed under CsBr‐rich conditions by Du et al. Interestingly, this excess CsBr enhanced the photoluminescence efficiency of the films (**Figure** **4**a), making them potentially suitable for incorporation in LEDs.^[^ ^76^ ^]^ Li et al. suggested that introducing excess PbBr_2_ can promote the formation of 2D CsPb_2_Br_5_, thereby increasing the grain size of the inorganic perovskite and effectively passivating the defects, making it beneficial for photovoltaic applications.^[^ ^77^ ^]^ Jie et al. remarked that the deposition rate ratio of inorganic precursors cannot reliably reflect the actual stoichiometry of the perovskite films.^[^ ^78^ ^]^ They found an optimal molar ratio of CsBr:PbBr_2_ = 0.7:1 during evaporation, yielding films with a composition that is closest to the targeted atomic ratio of Cs:Pb:Br =  1:1:3, and attributed this result to re‐evaporation of PbBr_2_ from the sample, which skews the ultimate ratio in the films. These studies underscored a crucial aspect of TE for perovskite deposition: it's not enough to start with the ideal precursor ratio to achieve the desired perovskite film stoichiometry. The tendency of some precursors to re‐evaporate from the substrate needs to be considered and compensated for. To achieve appropriate film characteristics, the stoichiometric ratio must be adjusted precisely for different applications.

Through adjustments of the A‐site cation composition, quasi‐2D perovskites are also fabricated by TE. In 2018, Fan et al. fabricated Ruddlesden‐Popper (RP) quasi‐2D perovskite (BA)_2_(MA)_3_Pb_4_I_13_ thin films using a single source to evaporate (BA)_2_(MA)_3_Pb_4_I_13_ (n = 4) powder.^[^ ^55^ ^]^ Here, in‐situ annealing was shown to improve the phase purity and crystallinity. La‐Placa et al. co‐evaporated phenethylammonium iodide (PEAI) and PbI_2_ to form 2D perovskite on 3D MAPbI_3_ (Figure 4b).^[^ ^79^ ^−^ ^81^ ^]^ Due to the lack of crystal orientation control of the 2D perovskite, the efficiency of charge transport through the 3D/2D composite was reduced. However, the orientation of the 2D compound can be adjusted by tuning the evaporation rate, which will be elaborated on further below. It was demonstrated that the n value of 2D perovskite significantly impacts the performance of optoelectronic devices. Unfortunately, the fabrication of a pure 2D phase and an exact *n* value using TE remains challenging.

Besides the composition tuning of A‐site cations and halide anions, some research groups have also adjusted the composition of the B‐site cations. For instance, Pb‐Sn alloyed B‐site cations resulted in perovskites with a narrow bandgap of 1.28–1.35 eV.^[^ ^80^ ^−^ ^82^ ^]^ Chen et al. replaced Pb with a Ge‐Sn alloy, realizing the TE of CsSn_0.5_Ge_0.5_I_3_ perovskite, and demonstrated better air stability and optical properties when compared with pure Sn or Pb‐based perovskite (Figure 4c).^[^ ^59^ ^]^ Luo et al. replaced Pb^2+^ by Eu^2+^ to fabricate CsEuBr_3_ by co‐evaporating CsBr and EuBr_2_.^[^ ^83^ ^]^ This composition exhibits efficient and stable blue emission and good exciton transport, and it is hence a promising candidate for blue perovskite LEDs.

Using other, less studied perovskite compositions such as Rb_3_CeI_6_, Cs_2_AgBiCl_6_,^[^ ^84^ ^]^ Cs_2_NaBiI_6_,^[^ ^85^ ^]^ Cs_2_Ag_x_Na_1‐x_Bi_y_In_1‐y_Cl_6_,^[^ ^86^ ^]^ it was shown that the ideal stoichiometric ratio could be achieved through TE. These studies demonstrate that TE can yield continuous and pinhole‐free films with uniform thickness even without any additional post‐deposition annealing. This is a significant advantage over solution‐processed films, which often require additional treatments to achieve similar quality.^[^ ^87^, ^88^ ^]^ This finding highlights the unique ability of TE to preserve the desired stoichiometry and morphology in multi‐component perovskite films. However, the TE films reported in these works typically have small grain sizes, leading to a higher density of grain boundaries, which limits the performance of optoelectronic devices. Therefore, further research is needed to develop strategies for mitigatin defects associated with grain boundaries and optimizing the microstructure of thermally evaporated films for improved device applications.

#### 2.2.4 Chamber Pressure

**Figure 5:**
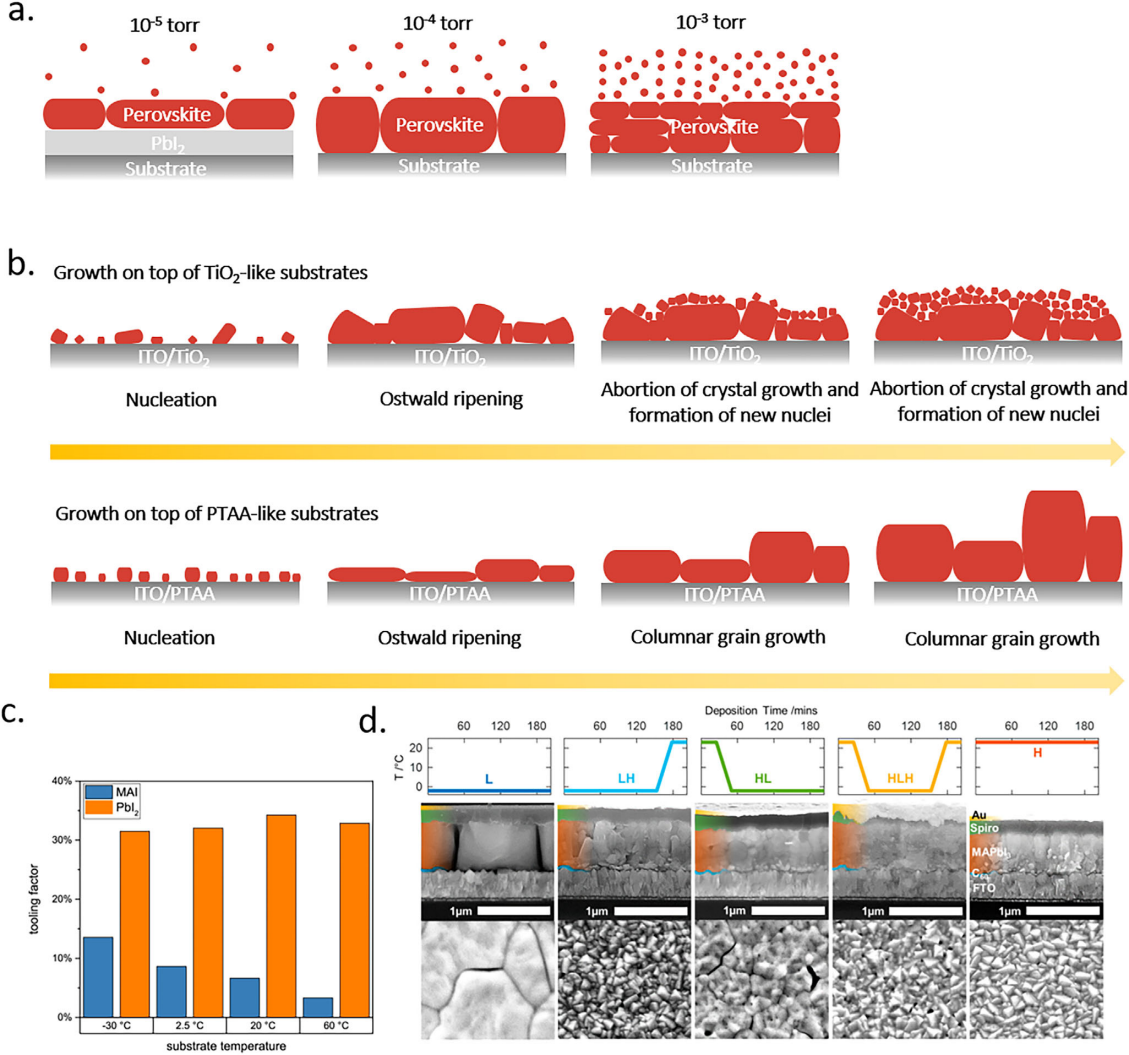
a) Impact of pressure on perovskite microstructure. Reproduced with permission. Copyright 2016, Wiley‐VCH.^[^ ^89^ ^]^ b) Impact of surface polarity on the growth of perovskite films. Reproduced with permission. Copyright 2021, Wiley‐VCH.^[^ ^89^ ^]^ c) Impact of substrate temperature on the tooling factors of MAI and PbI_2_. Reproduced with permission. Copyright 2020, American Chemical Society.^[^ ^101^ ^]^ d) The microstructure of MAPbI_3_ perovskite layers under varied substrate temperatures. Reproduced under the Creative Commons license.^[^ ^103^ ^]^

Chamber pressure was shown to influence the growth kinetics of perovskite, thereby affecting film quality and grain size. Therefore, controllable chamber pressure is essential for high‐quality TE perovskites. The chamber pressure is controlled by adjusting the temperature of the evaporation sources and can be fixed at a constant value by a tunable gate valve integrated with the chamber. Hsiao et al. controlled the partial pressure of the organic halide gas for the second step in a sequential TE MAPbI_3_ perovskite, finding that extreme pressure (high and low) leads to small grain sizes.^[^ ^89^ ^]^ Moreover, PbI_2_ cannot fully convert into perovskite under low‐pressure conditions. Large, micrometer‐sized grains only grew under medium‐pressure conditions (**Figure** **5**a). This result was confirmed independently by Arivazhagan et al., who found that co‐evaporated MAPbI_3_ possesses sufficient conversion, resulting in large grain sizes only with moderate background pressure.^[^ ^90^ ^]^ While these two studies demonstrated the impact of chamber pressure on the growth of TE perovskite, other works highlighted the difficulties in pressure control during the fabrication of TE organic‐inorganic perovskites. A key contributor to this is the fact that organic halide precursors such as MAI exhibit non‐directional evaporation behavior. This also leads to high and fluctuating background pressures in the range of 10^−5^ to 10^−4^ mbar during MAI deposition. The increasing background pressure probably originates from the gaseous side‐products of MAI degradation.^[^ ^91^, ^92^ ^]^ Similarly, FAI degrades under vacuum and thermal stress.^[^ ^93^, ^94^ ^]^ Kroll et al. described the effect of increasing background pressure on the growth of FA‐based perovskites.^[^ ^95^ ^]^ They pointed to several degradation reactions that occur upon heating and evaporation, rapidly increasing the fraction of degradation products with increasing source temperature. These degradation products increase the background pressure in the vacuum chamber. They seemingly reduce the mean free path of the evaporated molecules, promote scattering events, and impede the probability of FAI reaching the sample.

#### 2.2.5 Properties of the Substrate and Interfacial Layer

Sticking coefficient is the term used to describe the ratio of the number of adsorbate atoms (or molecules) that adsorb, or “stick,” to a surface to the total number of atoms that impinge upon that surface during the deposition.^[^ ^96^ ^]^ The sticking coefficient of substrates affects the condensation rate, which, in turn, influences the growth kinetics of TE perovskite films. A practical example of this is the inaccuracy in thickness measurements caused by the low sticking coefficient of organic precursors on QCMs. Borchert et al. pointed out that phosphorus‐containing impurities influence the adhesion of the MAI to the QCM.^[^ ^68^ ^]^ The presence of impurities leads to an overestimation of the actual deposition rate or the adhesion on the gold‐covered QCM surface. Klipfel et al. found that the substrate strongly affects the first 30 nm of the perovskite layer, which functions as a seed layer and influences the composition of the MAPbI_3_ layer after 30 nm.^[^ ^97^ ^]^ Similarly, Abzieher et al. found that the growth dynamics of perovskites are highly dependent on the underlying substrate material, affecting the crystallographic and morphological properties of the resulting thin films.^[^ ^98^ ^]^ Additionally, film quality depends on the surface polarity of the substrate (Figure 5b), with a non‐polar surface promoting optimal crystallization dynamics. Palechor‐Ocampo et al. similarly found that the substrate affects the stoichiometry of the evaporated material and consequently changes the electrical properties.^[^ ^99^ ^]^ Olthof et al. studied in detail the formation of MAPbI_3_ in the first tens of nanometers.^[^ ^100^ ^]^ They observed that chemical reactions take place at the interface, especially in the case of the metal oxides, whose catalytic properties lead to the formation of volatile products. The perovskite film grows only once the substrate is covered by physisorbed species, creating a passivating layer.

Substrate temperature also influences the properties of the TE perovskite films. Marcel et al. studied the influence of substrate temperature on the evaporation process for MAI and PbI_2,_ which they co‐evaporated to obtain MAPbI_3_ films (Figure 5c).^[^ ^101^ ^]^ They found that while the adhesion to the substrate is mainly constant for PbI_2_, the condensation of MAI is significantly reduced at higher substrate temperatures. This affects the final stoichiometry of co‐evaporated perovskite films. Kottokkaran et al. and Lohmann et al. have made similar observations regarding the reduced adhesion of MAI at elevated substrate temperatures.^[^ ^102^, ^103^ ^]^ They found that MAPbI_3_ films co‐evaporated onto a cold (−2 °C) substrate exhibited large, micrometer‐sized crystal grains, while films that formed at room temperature (23 °C) only produced grains of 100 nm (Figure 5d). This behavior is similar in CsPbBr_3_ inorganic perovskites, where substrate heating has led to smaller grain sizes but promoted better perovskite crystallization and smoother film surface.^[^ ^104^ ^]^

#### 2.2.6 Evaporation Rate

**Figure 6:**
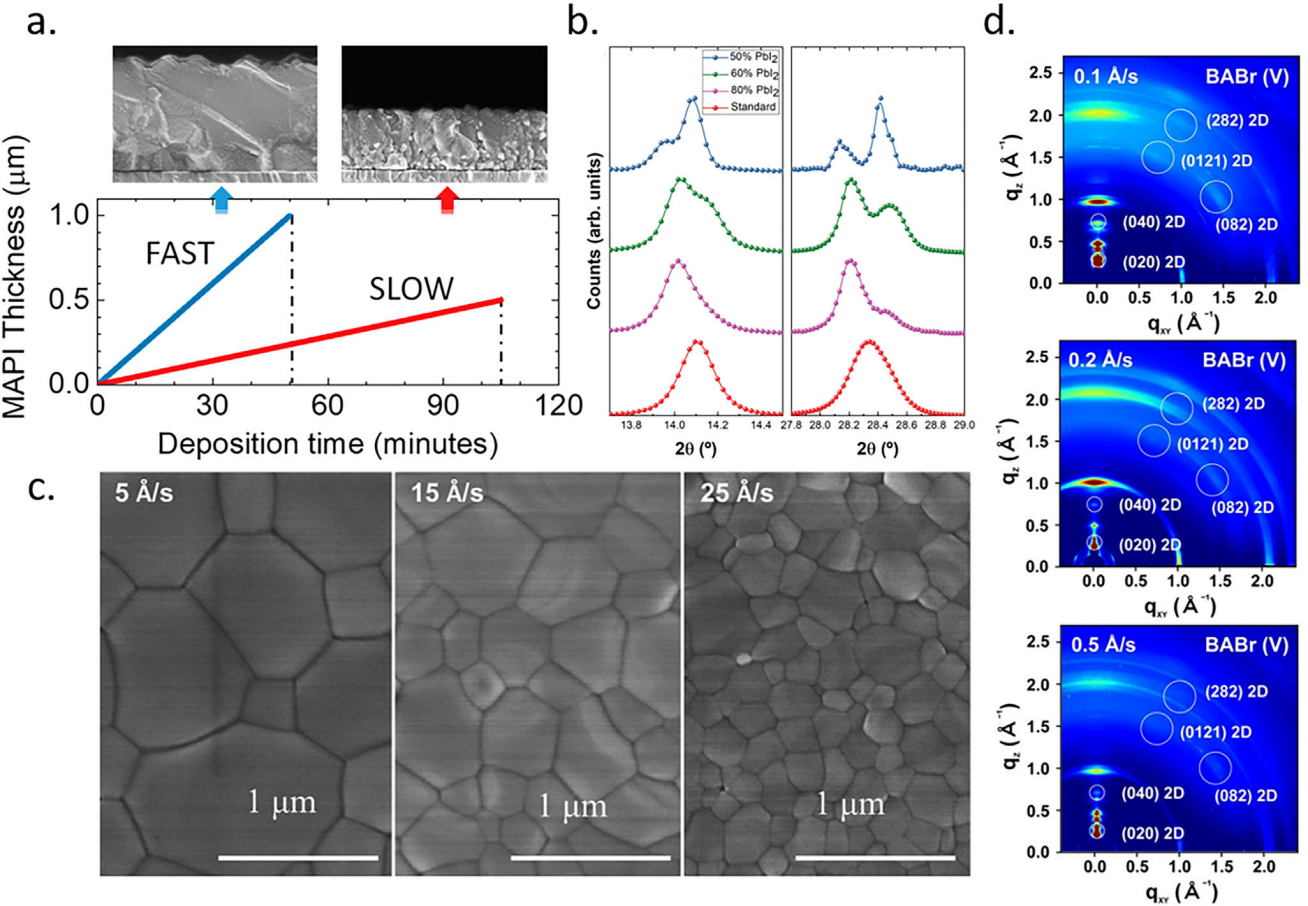
Impact of evaporation rate on perovskite layer: a) thickness, Reproduced with permission. Copyright 2023, American Chemical Society.^[^ ^46^ ^]^ b) Phase purity, Reproduced with permission. Copyright 2019, Wiley‐VCH.^[^ ^105^ ^]^ c) Microstructure, Reproduced with permission. Copyright 2018, Wiley‐VCH.^[^ ^37^ ^]^ and d) Orientation. Reproduced with permission. Copyright 2022, Royal Society of Chemistry.^[^ ^106^ ^]^

The composition of complex perovskites, containing multiple cations and halides, can be readily adjusted by controlling the evaporation rates of each precursor during TE. However, even when the ratio between the precursors is kept constant, the absolute evaporation rates themselves significantly impact the properties of the resulting films. Piot et al. found that TE MAPbI_3_ films deposited under slow evaporation show relatively low optical absorption.^[^ ^46^ ^]^ In contrast, rapid evaporation generates compact and void‐free films with much stronger light absorption (**Figure** **6**a). They also found that TE MAPbI_3_ exhibits a tetragonal phase, the same as solution‐processed MAPbI_3_, under low evaporation rates but forms a more stable cubic phase under fast evaporation conditions (Figure 6b).^[^ ^105^ ^]^ The scanning electron microscopy (SEM) images show that larger grain sizes can be obtained at a rapid evaporation rate.

Interestingly, the impact of the evaporation rate is different in TE CsPbBr_3_. Chen et al. found that a large‐grain microstructure can be generated when using a low evaporation rate (Figure 6c).^[^ ^37^ ^]^ Increasing the evaporation rate led to a dramatic reduction in the grain size and an inhomogeneous grain distribution. The evaporation rates did not alter the cubic phase of CsPbBr_3_ but strongly influenced their crystallinity and crystal orientation. They also found that a lower evaporation rate led to higher crystallinity and a crystal orientation along the (ℓ00) planes. Li et al. similarly reported that, in TE CsPbBr_2_I, a fast evaporation rate increases the nucleation probability and promotes the generation of small grains. They explained this experimental result considering that besides the changes in grain size, the excess kinetic energy from the high‐rate evaporation probably leads to swift reflection and re‐evaporation into the vacuum, causing pinholes on the surface. On the other hand, the low evaporation rate may cause a high carrier recombination rate.

For 2D TE perovskites, the evaporation rate was shown to affect the crystal orientation. The growth of vertically oriented 2D perovskite by vapor deposition was reported by Choi et al.^[^ ^106^ ^]^ A (BA)_2_(MA)_n‐1_Pb_n_I_3n‐1_Br_2_ 2D surface layer was deposited by a reaction of BABr vapor with MAPbI_3_ thin films. GIWAXS patterns (Figure 6d) confirm the presence of Bragg spots in a q_z_ range of 1.0–2.0 Å^−1^ corresponding to the (2 8 2), (0 12 1), and (0 8 2) planes of the RP phase perovskite when the deposition rate is 0.1 Å/s. This finding indicates that the RP perovskite preferentially grows perpendicular to the perovskite surface. Significantly, vertically oriented 2D surface perovskite does not hinder the charge transport in the vertical direction. At a higher deposition rate of 0.5 Å/s, continuous Debye–Scherrer diffraction rings indicate an increasing degree of isotropic orientation.

#### 2.2.7 Additives

Additive engineering has proven to be a powerful technique for enhancing the quality of solution‐processed perovskites. By introducing small amounts of compounds like MACl in FAPbI_3_,^[^ ^107^ ^]^ methylammonium acetate (MAAc) in MAPbI_3_,^[^ ^108^ ^]^ dimethylammonium iodide (DMAI) in CsPbI_3_,^[^ ^109^ ^]^ or SnF in FASnI_3_,^[^ ^110^ ^]^ various benefits can be introduced, such as reducing the formation energy of perovskite to improve crystal quality, maintaining the stability of the desired phases, preventing side reactions, or reducing the trap density. Unfortunately, this strategy is not directly transferable to thermally evaporated perovskites since most additives commonly used in solution processing have low decomposition or sublimation temperatures. This means that they tend to re‐evaporate from perovskite films during thermal annealing, rendering them ineffective. Therefore, developing new additives specifically tailored to the requirements of TE is crucial. These additives must withstand the process's high temperatures and remain incorporated within the perovskite film to exert their beneficial effects. This represents a key challenge and opportunity for further research in the field of TE perovskites.

Tuning the halide anions within the lattice allows for adjusting the band gap of halide perovskites, which has direct implications to their optoelectronic properties. In addition, Cl has been shown to enhance the crystallinity and grain size by reducing the film formation energy during the growth of MA and FA‐based perovskites. Building on the knowledge gained from solution‐processed perovskites, the most common additives in TE are various chlorides such as PbCl_2_,^[^ ^45^, ^111^ ^]^ MACl,^[^ ^112^ ^]^ and CsCl.^[^ ^113^ ^]^ Similar to the finding in solution‐processed counterparts, above chlorides were shown to enhance the grain sizes of halide perovskites in co‐evaporation and sequential evaporation.

**Figure 7:**
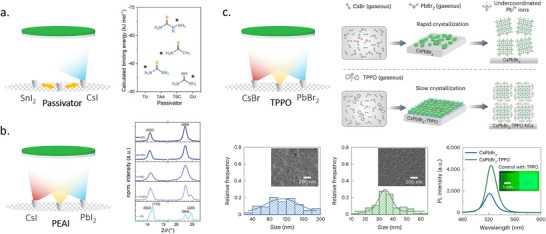
Use of additives in TE of a) Sn‐based perovskites. Reproduced with permission. Copyright 2021, Wiley‐VCH.^[^ ^114^ ^]^ b) CsPbI_3_. Reproduced with permission. Copyright 2021, Wiley‐VCH.^[^ ^115^ ^]^ and c) CsPbBr_3_. Reproduced with permission. Copyright 2021, Springer Nature.^[^ ^118^ ^]^

Another additive borrowed from solution processing is SnF, which was shown to prevent the oxidation of Sn^2+^ into Sn^4+^ in Sn‐based perovskite. Due to its high decomposition and sublimation temperatures, it is compatible with the TE.^[^ ^80^, ^82^ ^]^ Li et al. explored alternative additives, including thiourea, thioacetamide, thiosemicarbazide, and guanidine, for TE CsSnI_3_ to replace SnF (**Figure** **7**a).^[^ ^114^ ^]^ They found that the first three compounds, similarly to SnF, can effectively retard the oxidation of Sn^2+^ in CsSnI_3_ films due to the presence of the Lewis‐base S═C─N functional group. Thiosemicarbazide can increase the grain size, reduce trap density, and improve the stability of TE CsSnI_3_.

CsPbI_3_ is prone to unwanted phase transitions, which can negatively impact its performance in devices. To address this challenge, additives were introduced to improve phase stability. A noteworthy example is PEAI, which has been shown to stabilize the γ‐phase in co‐evaporated CsPbI_3_ and promote a preferred crystal orientation along the (0 0 l) plane, and suppress the less desirable the (1 1 0) and (2 2 0) orientations (Figure 7b), while also reducing the trap density.^[^ ^115^, ^116^ ^]^ Mali et al. reported that guanidinium iodide (GAI) has a similar effect on the phase stability of TE γ‐CsPbI_3_ as PEAI.^[^ ^117^ ^]^

The additives mentioned above were initially explored in the context of solution processing before they were explored in TE, where they showed similar beneficial effects. However, some additives, such as thiosemicarbazide, PEAI, and GAI, behave similarly to MAI during evaporation, making it challenging to control their TE. Therefore, it is necessary to develop additives that can evaporate unidirectionally and do not decompose into gaseous products.

Recently, Tang et al. introduced a multifunctional Lewis‐base additive, triphenylphosphine oxide (TPPO), which simultaneously served as a crystallization retardant, size‐constrained ligand, and a surface passivator (Figure 7c).^[^ ^118^, ^119^ ^]^ Remarkably, this additive is effective in a variety of perovskite compositions, including CsPbBr_3_, CsPbBr_x_Cl_3‐x,_ and MAPbBr_3_. For instance, high‐quality TE nanocrystal films were incorporated as the active layer in blue LEDs, achieving the highest performance among comparable devices. TPPO has also been successfully used in OLED devices fabricated by the TE, further proving its compatibility with the TE process.^[^ ^120^, ^121^ ^]^ This work is significant because it showcases an additive specifically designed for TE, independent of solution processing. It opens up new possibilities for developing and utilizing additives tailored to the unique requirements of TE, paving the way for which points out the direction for future development of additives in TE perovskites.

Dopants are a special type of additive that profoundly impact the electrical characteristics of TE perovskites and fine‐tune their electronic properties. Schram et al. demonstrated both n‐ and p‐type doping of FACsPb(IBr)_3_ using co‐evaporation of tetrakis (hexahydropyrimidinopyrimidine) ditungsten(II) (W2(hpp)_4_) and 2‐(7‐dicyanomethylene 1,3,4,5,6,8,9,10‐octafluoro‐7H‐pyrene‐2‐ylidene)‐malononitrile, respectively.^[^ ^122^ ^]^ By carefully controlling the amount of these molecules, they could shift the Fermi level of TE perovskites within a range of 900 mV. Furthermore, sequential deposition of n‐ and p‐type perovskite layers was demonstrated to form PN or NP junctions that featured the characteristic rectification behavior.

#### 2.2.8 Post Treatment

In addition to methods that affect the TE process during deposition, post‐treatments are also widely applied to further improve the crystal quality of TE perovskites. The most common post‐treatment for TE perovskites is annealing, which involves heating the deposited film to a specific temperature for a certain duration. Post‐annealing is almost always necessary for sequentially deposited TE perovskites as it provides the energy required to promote the diffusion of organic cations into the PbI_2_ framework, which is necessary to convert from precursors to perovskite. However, this treatment is not always required for co‐evaporated perovskites. Ji et al. studied the effect of post‐annealing on FACs‐based TE perovskite with different stoichiometries.^[^ ^70^ ^]^ They found that post‐annealing is beneficial when the film is deficient in A‐site cations, has no noticeable effect on samples with stoichiometric balance, and can even adversely impact films that are A‐site cation‐rich. This suggests that in the ideal stoichiometric case, the co‐evaporation process itself provides sufficient energy for precursors to convert into perovskite completely, rendering post‐annealing unnecessary.

Multiple reports suggest that the annealing atmosphere also affects the quality of TE perovskite. Huang et al. observed that the charge carrier transport in CsPbI_3_ becomes more efficient if annealing is performed in ambient air,^[^ ^75^ ^]^ which was attributed to a reduction in the trap density of states and suppression of the nonradiative recombination in the presence of oxygen.^[^ ^123^, ^124^ ^]^ Similar observations were made regarding FA‐based perovskites.^[^ ^44^, ^45^ ^]^

Beyond simple thermal annealing, other post‐treatment methods can be beneficial. Lu et al. developed a vapor‐assisted post‐treatment method in which a film of the yellow phase FAPbI_3_ was converted to a highly crystalline black phase by vapor exposure to methylammonium thiocyanate or formamidinium thiocyanate at 100 °C.^[^ ^125^ ^]^ Although the perovskite was initially solution‐processed in this work, the post‐treatment itself is solvent‐free and aligns with the broader goal of minimizing or eliminating solvent use in perovskite fabrication.

#### 2.2.9 Photovoltaic Devices Based on TE Perovskites

The performance of thermally evaporated perovskite photovoltaics has progressed from an initial 15.4% to over 26%, now rivalling solution‐processed counterparts.^[^ ^25^, ^42^ ^]^ This advancement primarily stems from evolving crystal growth modulation strategies, enabling high‐quality fabrication of perovskite systems that have expanded from the MA‐based perovskites explored in the early research stages to FA‐based and Cs‐based compositions. Newly developed strategies effectively reduced the bulk defect densities in perovskite films, leading to an increase in the efficiency of the photovoltaic devices. Furthermore, interfacial engineering techniques adapted from solution‐process methodologies have diminished surface defect densities, enhancing photovoltaic performance. Most research efforts are dedicated to the deposition of the perovskite active layer by TE, while the charge transport layers are primarily processed from solution. Nevertheless, various reports employed solvent‐free deposition approaches that demonstrate comparable functionality.^[^ ^126^ ^−^ ^129^ ^]^

Unlike solution‐processed perovskites, TE perovskite fabrication demonstrated enhanced device performance through the two‐step deposition procedure and not the one‐step methodologies. This superiority originates from the inherent challenges in controlling crystallization under vacuum conditions, where separated precursor deposition and nucleation/growth processes substantially simplify crystallization management. Some TE‐formed devices exhibit intrinsic advantages in the context of device stability – a multifaceted aspect influenced by crystallinity, defect concentration, bond strength, and intrinsic decomposition temperature.^[^ ^62^ ^]^ These benefits are likely related to the in‐situ growth processes without annealing that yield nearly tensile‐stress‐free compact films with exceptional interfacial robustness. Such characteristics confer intrinsic stability even to metastable MAPbI_3_, emphasizing that stress‐minimized growth processes critically enable perovskite properties to exceed existing limitations and potentially surpass chemical engineering approaches for stability enhancement.^[^ ^62^ ^]^ TE perovskite photovoltaics extensively incorporate achievements from solution‐based systems, including additive selection, transport layer optimization, and surface passivation strategies. Herein, we systematically summarize key performance‐enhancing strategies and corresponding photovoltaic parameters in the developmental trajectory of TE‐processed PSCs in **Table** **1**.

**Table 1:** Summary of PSCs based on TE.

Perovskite	Small area photovoltaic parameters				Stability	Area	Evaporation procedure	Architecture	Year	Key strategy	Refs.
	*V*_oc_ [V]	*J*_sc_ [mA cm^−2^]	FF [%]	PCE [%]							
MAPbI_3_	1.07	21.5	67	15.4		0.076 cm^2^	co‐evaporation	n‐i‐p: FTO/TiO_2_/perovskite/Spiro‐OMeTAD/Ag	2013	Co‐evaporation of PbCl_2_ and MAI	[25]
MAPbI_3_	1.06	22.69	73.2	17.6	–	0.1 cm^2^	sequential	n‐i‐p: ITO/Ca/C_60_/perovskite/TAPC/TAPC:MoO_3_/MoO_3_/Ag	2016	Tuning background chamber pressure during MAI evaporation	[89]
MAPbI_3_	1.14	22.08	80.5	20.3	–	0.1 cm^2^/1 cm^2^ (PCE = 15%)	co‐evaporation	p‐i‐n: ITO/TaTm:F_6_‐TCNNQ/TaTm/perovskite/C_60_/C_60_:Phlm/Ag	2016	Doping of transport layer	[130]
MAPbI_3_	1.12	23.3	77.7	20.28	98% of initial PCE after 110 days under 35% RH	0.16 cm^2^/1 cm^2^ (PCE = 18.97%)/4 cm^2^ (PCE = 16.59%)/16 cm^2^ (PCE = 11.22%)/21 cm^2^ module (PCE‐18.13%)	co‐evaporation	n‐i‐p: FTO/SnO_2_(TiO_2_)/perovskite/Spiro‐OMeTAD/Ag	2020	Combining effort on TE deposition optimization, surface treatment, interfacial passivation, and optical management	[131]
MAPbI_3_	1.15	22.43	79.6	20.5		0.16 cm^2^	co‐evaporation	p‐i‐n: ITO/PTAA(MeO‐2PACz)/perovskite/C_60_/BCP/Cu	2020	Tuning substrate temperature and transporting layer	[101]
FAMACsGAPbI_x_Br_3‐x_	1.148	18.5	78.4	16.5	–	–	co‐evaporation	p‐i‐n: ITO/MoO_3_/TaTm/perovskite/C_60_/BCP/Ag	2021	Fixing CsI amount and controlling PbI_2_ and PbBr_2_ ratio to tune the perovskite bandgap	[71]
	1.154	17.8	78.7	16.3	–						
	1.183	17.1	81.1	16.5	100% of initial PCE after 500 h in dark for encapsulated device/ 90% of initial PCE after 340 h under 1sun at 25 °C in N_2_						
	1.21	16.7	76.9	15.7	–						
FACsPbI_3_	1.11	24.88	77	21.3	101% of initial PCE after 189 days under 35% RH under dark	0.09 cm^2^/9.6 cm^2^ module (PCE = 14.6%)	sequential	n‐i‐p: FTO/TiO_2_/perovskite/Spiro‐OMeTAD/Au	2021	Low‐temperature vacuum annealing	[132]
FAMAPbI_3_	1.05	25.7	76	20.4	(MAI = 32%)100% of initial PCE after 1000 h under light	0.16 cm^2^/1 cm^2^ tandam (PCE = 24.7%)	co‐evaporation	p‐i‐n: ITO/MeO‐2PACz/perovskite/C_60_/BCP/Ag	2021	Tuning MAI/FAI ratio/ wash MeO‐2PACz surface	[133]
MAPbI_3_	1.12	22.3	82.4	20.61	98% of initial PCE after 1000 h under 30% RH/ 80% of initial PCE after 500 h under 85 °C and 10% RH/ 80% of initial PCE after 100 h under AM1.5	0.086 cm^2^/1 cm^2^/1.96 cm^2^	co‐evaporation	p‐i‐n: ITO/Spiro‐TTB(PTAA or MeO‐2PACz)/perovskite/PCBM/BCP/Ag	2021	Tuning the background chamber pressure during the growth process to creat graded composition and Fermi levels	[134]
CsPbI_3_	1.09	17.33	79.41	15	60% of initial PCE after 120 h under 80 °C with encapsulation	0.045 cm^2^	co‐evaporation	p‐i‐n: ITO/PTAA/perovskite/C_60_/BCP/Cu	2021	PEAI additive	[115]
CsPbI_3_	1.1	18.3	80.9	16.3	94% of initial PCE after 30 days in N_2_ under dark	0.09 cm^2^	co‐evaporation	n‐i‐p: FTO/TiO_2_/perovskite/Spiro‐OMeTAD/Au	2021	tuning stoichiometry and annealing condition	[75]
FACsPbI_3_	1.15	25.92	81.78	24.42	97% of initial PCE after 1300 h under 35% RH under dark/ no degradation after 4000 h under <5% RH under dark/70% of initial PCE after 1200 h in Ar at 60 °C under dark/92% of initial PCE after 450 h under MPP tracking in N_2_	0.1 cm^2^/1 cm^2^ (PCE = 23.44%)/14.4 cm^2^ (PCE = 19.87%)	sequential	n‐i‐p: FTO/TiO_2_/perovskite/Spiro‐OMeTAD/Au	2022	Chloride anion introduced with PbCl_2_; separate Inorganic and organic deposition into two chamber;	[45]
FAMACsGAPbI_x_Br_3‐x_	1.148	17.3	80	15.9	75% of initial PCE after 700 h under 85 °C	–	co‐evaporation	p‐i‐n: ITO/PTAA/perovskite/C_60_/BCP/Ag	2022	CsMAFAGA quadruple‐cation	[72]
FAMAPbI_3_	1.06	23	79	19.3	–	0.25 cm^2^/1 cm^2^	co‐evaporation	p‐i‐n: ITO/PTAA/perovskite/C_60_/BCP/Ag	2022	introducing 20% PbCl_2_ to replace PbI_2_	[135]
FACsPbI_3_	1.175	26.47	84.94	26.41	95% of initial PCE after 2800 h under 10% RH and 25 °C under dark/ 84% of initial PCE after 1500 h in Ar at 60 °C/80% of initial PCE after 600 h under light in N_2_	0.1 cm^2^/1 cm^2^ (PCE = 24.88%)	sequential	n‐i‐p: FTO/SnO_2_/perovskite/T_2_(Spiro‐OMeTAD derivative)/Au	2024	replacing spiro‐OMeTAD with the derivative with thiomethyl‐substituted fluorene; separate Inorganic and organic deposition into two chamber; MACl additive	[42]
MAPbI_3_	1.19	23.1	81	22.5	61% of initial PCE after 1000 h under 70% RH and 25 °C under dark/ 74% of initial PCE after 500 h in N_2_ at 85 °C/80% of initial PCE after 500 h under AM1.5 in N_2_	0.135 cm^2^/ 1 cm^2^ (PCE = 21.9%)/9 cm^2^ module (PCE = 21.0%) /25 cm^2^ (PCE = 12.3%)/25 cm^2^ module (PCE = 20.5%)	co‐evaporation	p‐i‐n: ITO/TAA‐tetramer/perovskite/C_60_/BCP/Ag	2025	replacing PTAA with TAA‐tetramer	[129]

#### 2.2.10 Conclusion on Thermal Evaporation

A long‐standing challenge in solvent‐free perovskite fabrication was to achieve a degree of crystallization control comparable to that in solvent environments to enable the growth of high‐quality crystalline films. The procedure to separate the deposition processes of metal halides and organic halides, combined with chloride ion additives, facilitated satisfactory outcomes and led to photovoltaic devices with efficiencies exceeding 26%. This demonstrates that state‐of‐the‐art TE techniques now possess capabilities to control crystal growth on par with solution‐based methods, marking a new phase of development. Current challenges in the field include:

- Throughput limitations: TE exhibits a significant throughput disadvantage compared to blade‐coating and other techniques. It is limited with regard to deposition rates and requires separate precursor deposition and post‐annealing steps. Developing high‐throughput deposition strategies is critical to promoting industrial adoption.
- Solution‐dependent functional layers: High‐performance TE‐based photovoltaic devices still rely on solution‐processed functional layers (e.g., charge transport layers). Advancing fully vapor‐deposited devices is essential for industrial scalability.
- Limited material compatibility: TE has only demonstrated efficient optoelectronic devices based on a small number of perovskite systems, e.g., MAPbI_3_ and FAPbI_3_, whereas solution‐based methods have been validated across many compositions. The optimization of material‐specific TE processes is required to expand the appeal of solvent‐free perovskite fabrication.
- Nanostructure versatility: While TE excels in producing high‐quality films for photovoltaics, its application in micro‐ and nanostructured films (e.g., for LEDs) demands dedicated investigations.

In summary, TE‐based perovskite deposition continues to evolve. Although a gap in the maturity of TE and solution‐based techniques persists, partly due to disparities in the extent of research commitments, the overall trajectory is promising.

### 2.3 Magnetron Sputtering

#### 2.3.1 Mechanism and Advantages of Magnetron Sputtering

**Figure 8:**
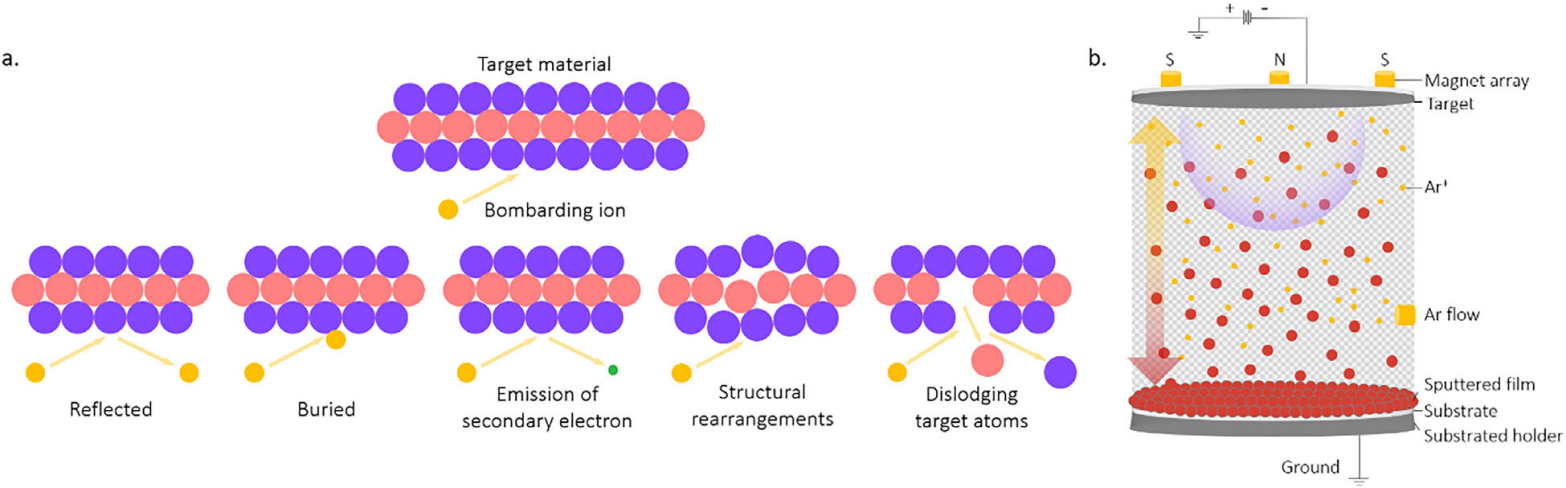
a) A schematic of possible interactions between the bombarding high‐energy ions and the target surface. b) An illustration of the sputtering process involving an RF magnetron.

Magnetron sputtering is typically performed in a moderate vacuum with a background pressure of 1–100 µbar created using an inert gas. A plasma is generated, and energetic, positive ions bombard the surface of a target, causing the ejection of atoms, ions, or molecules. A thin film forms when these species condense on a substrate. Several different interactions can occur between the bombarding high‐energy ions and the target surface, as shown in schematic **Figure** **8**a. These interactions ultimately determine the efficiency and characteristics of the sputtering process and the resulting thin film.

Two main theoretical models are used to describe the sputtering process.^[^ ^136^ ^]^ According to the thermal‐vaporization theory, the surface of the target vaporizes, releasing atoms into vacuum, when reaching a sufficiently high temperature, as a result of the bombardment with energetic ions. On the other hand, as per the momentum‐transfer theory, the incident particles transfer their momentum to atoms on the target surface atoms, making them lose. For efficient momentum transfer, the atomic weight of the sputtering gas, usually Ar, should have an atomic weight similar to that of the target. During stable conduction conditions, electrons are emitted from the cathode and accelerated towards the positive electrode, ionizing the sputtering gas molecules (e.g., argon). The ionized atoms (Ar^+^) are then impinging on the surface of the cathode electrode, i.e., the target. As a result, secondary electrons and sputtered atoms are produced, leading to further gas ionization, which is sputtered away from the surface.^[^ ^137^ ^]^ Direct current (DC) sputtering is compatible with conductive targets and has been widely applied for growing metal oxide thin films from a metal target and an Ar and O_2_ gas mixture that serves as a reactive gas. In the contrasting case of insulators such as oxides, charges from Ar ions remain localized upon impact and gradually build up on the target until further surface bombardment becomes impossible. This charge accumulation can be neutralized by applying an radio frequency (RF) potential to the target, where sequential bombardment with positive ions and electrons occurs. Due to the higher mobility within the plasma than the ions, more electrons will reach the insulating target surface during the positive half cycle than positive ions during the negative half cycle. Hence, the target will accumulate negative charges and develop a negative self‐bias that repels electrons near the target, creating a cloud of positive ions in front of the target surface. These ions will continue bombarding the target, and continuous sputtering can be achieved. It is possible to further increase the sputter yield by adding a strong magnetic field along with the RF power source that confines the charged plasma close to the target surface. Electrons emitted at a certain angle from the target are subject to Lorentz forces that induce a helical motion. This trajectory prolongs the electron residence time in the discharge area, enhancing the deposition rate by increasing the effective path length and probability of ion collisions. Sputtered atoms are primarily neutral, much heavier, and unaffected by the magnetic field. A schematic of the sputtering process involving an RF magnetron technique is shown in Figure 8b.

Sputter deposition offers several advantages over other thin‐film deposition methods:

- Materials with high vaporization temperatures can be processed into thin films at much lower temperatures than evaporation methods.
- A stoichiometric deposition is possible.
- By regulating/adjusting parameters like sputtering power and gas pressure, film thickness and composition, can be precisely controlled, which might enable tailored perovskite properties such as an optimal bandgap.
- Sputtered films feature better adhesion than other methods, even at room temperature, making them suitable for flexible and textured substrates at low and room temperature and further facilitating their integration in a wide range of device architectures.
- The films are highly uniform and compact, and the deposition rate is substantially higher than other techniques.
- By using several targets in sequence, multiple distinct layers can be deposited without breaking the vacuum, thereby facilitating multi‐component perovskite films and other layer preparation, e.g., charge transport layers and electrodes for complete device fabrication.
- The complete omission of solvents and deposition at room temperature considerably reduces its environmental footprint.
- The technique has a high utilization ratio of materials.
- Can be readily scaled to large areas (up to 3 × 6 m^2^ or roll‐to‐roll), making it suitable for industrial transfer and commercialization.

While sputtering offers numerous advantages for perovskite deposition, it is important to acknowledge its limitations. For instance, particles with high kinetic energy may damage sensitive functional underlying layers. This concern is particularly relevant when dealing with organic materials. In addition, it is challenging to obtain large perovskite grains, and porous scaffold layers underneath the perovskite layer can lead to voids, compromising device quality. Overcoming these challenges is essential for successfully transitioning this laboratory‐scale research to industrial‐scale manufacturing.

The following section outlines the current state of progress in magnetron sputtering of MHPs. First, the deposition of inorganic lead‐based components is introduced, which requires a post‐treatment to convert them into perovskites. Next, advances in single‐source sputtering of hybrid perovskites are summarized, including the latest achievements in applications in high‐performance solar cells. Finally, the deposition of all‐inorganic large bandgap perovskites is described, which is of interest for other optoelectronic applications such as photodetectors.

#### 2.3.2 Sputtering PbO, PbI_2_, or PbS and Subsequent Conversion into Perovskites

Sputtering can be used to create perovskite films in two main ways: either directly depositing perovskite films from a perovskite target or sequentially depositing Pb‐based compositions (e.g., PbO, PbS, or PbI_2_) and organic halide salts. Zhang et al. reported the deposition of PbO by DC reactive magnetron sputtering from a pure metallic Pb target using an Ar/O_2_ gas mixture in 2017.^[^ ^138^ ^]^ The as‐deposited PbO film was converted to MAPbI_3_ by dipping it into an isopropanol solution of MAI, followed by annealing at 100 °C for 30 min. The resulting perovskite films exhibited good surface morphology with grain sizes ranging from 50 to 600 nm and good coverage. Champion solar cells with active areas of 0.09 cm^2^ and 1.07 cm^2^ exhibited PCE of 14.1% and 10.7%, respectively, in the following device structure: FTO/ nanocrystalline rutile titania (NRT)/MAPbI_3_/Spiro‐MeOTAD/Au. The authors highlighted the capability of low‐temperature processed NRT to extract photogenerated electrons effectively.

In a similar study, in 2020, perovskite thin films were conformally deposited on 100 cm^2^ textured Si substrates using a two‐step vacuum process. There, Hwang et al. converted thin films of a sputtered PbO precursor by passing MAI vapor over PbO thin films in a two‐zone furnace CVD process for 30 min.^[^ ^139^ ^]^ The conversion of PbO into perovskite was confirmed by X‐ray diffraction (XRD) and TEM–EDS measurements, albeit a residual layer of PbO was present at the TiO_2_/perovskite interface. It was also found that a pre‐treatment, which converts PbO into PbI_2_, is necessary to convert it into MAPbI_3_ sequentially. A device (FTO/c‐TiO_2_/MAPbI_3_/Spiro‐MeOTAD/Au) with an area of 1.5 × 1.5 cm^2^ resulted in 10.2% PCE with a sputtered‐PbO precursor, whereas a PCE of 15% was achieved for spin‐coated PbI_2_ precursor.

**Figure 9:**
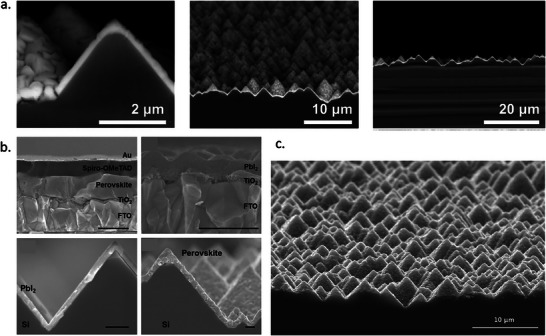
a) Conformal perovskite on a textured silicon surface. Reproduced under the Creative Commons license.^[^ ^140^ ^]^ b) Cross‐sectional SEM images of a completed solar cell, sputtered PbI_2_ film on a dense TiO_2_/FTO substrate, PbI_2_ film deposited on Si substrate, and perovskite film deposited on Si substrate. Reproduced with permission. Copyright 2017, IOP Publishing on behalf of the Japan Society of Applied Physics.^[^ ^141^ ^]^ c) SEM image of conformal perovskite on 100 cm^2^ textured substrate. Reproduced with permission. Copyright 2017, Elsevier.^[^ ^139^ ^]^

In the same year, Lee et al. reported a perovskite film on textured silicon with a dry two‐step conversion process (**Figure** **9**a).^[^ ^140^ ^]^ There, a conformal PbO_x_ precursor layer was deposited by RF magnetron sputtering, MAI powder was directly spread over the precursor film, and film deposition was finalized through thermal annealing in air. Once the reaction was complete, the remaining MAI was removed by a stream of N_2_ gas, rinsed with isopropanol, and dried by spinning. The final films were investigated using micro‐photoluminescence (µ‐PL), micro‐light beam‐induced current (LBIC) 3D mapping, stress simulation, and conductive atomic force microscopy (c‐AFM). It was concluded that subjection to bending and stress has a disadvantageous effect on the device performance, and HF vapor treatment of PbO films and substrate chemical rounding can be effective for stress‐relaxing and hence enhance the film quality.

Raifuku et al. reported the fabrication of MAPbI_3_ by sputter deposition of PbI_2_ films from a PbI_2_ target and subsequent exposure to MAI gas at 100 °C.^[^ ^141^ ^]^ The authors observed that the sputter‐processed perovskite films exhibit characteristics similar to those of their solution‐processed counterparts but reported a low PCE of 1.84% for a device structure of FTO/TiO_2_/MAPbI_3_/Spiro‐MeOTAD/Au (Figure 9b). The same team also showed that the substrate was fully covered with a pinhole‐free layer with grains with diameters as large as 500 nm. In addition, that study also demonstrated that sputtering could create a homogeneous phase‐pure MAPbI_3_ film even on rough substrates like textured Si. This is a significant step forward, as it opens possibilities for using sputtered perovskites in tandem solar cells.

More recently, Hwang et al. developed a two‐step sputtering process for creating PSCs.^[^ ^142^ ^]^ They used a PbI_2_ target to first deposit a 200 nm PbI_x_ film at 50 W RF power and 9 µbar Ar pressure. This film was then treated with a series of steps to improve its properties, including post‐processing through iodination, DMSO treatment, and thermal annealing to adjust the stoichiometry, crystallinity, and surface morphology. Next, the PbI_2_ films were converted into perovskite using a direct contact and intercalation process similar to Lee et al., where MAI powder was spread over the PbI_2_ film surface and heated at 180 °C for 30 mins.^[^ ^140^ ^]^ Further improvements of the interfacial and bulk properties of the MAPbI_3_ films were achieved by methylamine (MA) vapor annealing. This process resulted in high‐quality PSCs with a device architecture of FTO/TiO_2_/MAPbI_3_/Spiro‐OMeTAD/Au, which yielded a PCE of 12.2% as compared to ∼ 4% of completely untreated devices. It was also shown that a good‐quality uniform perovskite film can be processed on a 25 cm^2^ textured silicon surface, paving the way for Si/perovskite tandem solar cells through sputter deposition (Figure 9c).

Based on the dry‐conversion processed MAPbI_3_ layers discussed above, the same group introduced an additional anion exchange step to replace iodide with bromide by dipping films into a MABr/isopropanol solution, resulting in perovskite films with a wider bandgap.^[^ ^143^ ^]^ The bandgap of the mixed halide perovskite films was increased with dipping time, and a uniform depth profile of the perovskite composition was observed by time‐of‐flight secondary ion mass spectrometry. It was also shown that, when processed into solar cells, an enhanced open circuit voltage (V_oc_) and improved PCE, increasing from 3.4% to 4.1%, can be obtained by regulating the I/Br ratio through the ion‐exchange method. This demonstrates the potential of this simple ion exchange method as an effective post‐deposition method for fine bandgap tuning.

In addition to PbO and PbI_2_, as‐deposited sputtered amorphous PbS thin films can also be used to grow homogeneous and pinhole‐free MAPbI_3_ thin films. In this case, PbS layers are converted first into PbI_2_ through exposure to I_2_ gas at room temperature, then the film is dipped into MAI solution for a few minutes and finally annealed at 100 °C for 20 min, as reported by Silva Filho et al. in 2018.^[^ ^144^ ^]^ The successful conversion of PbI_2_ into MAPbI_3_ was confirmed by various characterization methods, including EDS, XRD, optical absorption, photoluminescence, FTIR, and Raman spectroscopy.

#### 2.3.3 Direct Sputtering of Organic–Inorganic Perovskites and Post‐Processing

Bonomi et al.^[^ ^145^ ^]^ successfully deposited MAPbI_3_ thin films by RF‐magnetron sputtering from a single target consisting of MAI and PbI_2_ with a 30% w/w excess of MAI to compensate for its higher volatility.^[^ ^143^ ^]^ The resulting films were phase‐pure, with a negligible (barely detectable) content of unreacted PbI_2_ and full surface coverage. However, SEM imaging revealed the presence of cracks in thicker films, indicating a potential limitation of this approach. While the light absorption and emission of the sputtered films were comparable to those of as‐grown solution‐processed MAPbI_3_ films, their photoluminescence lifetime was only a few nanoseconds, suggesting significant losses due to nonradiative recombination. The authors proposed that post‐growth treatments could potentially passivate defects to improve the photoluminescence quantum yield.

**Figure 10:**
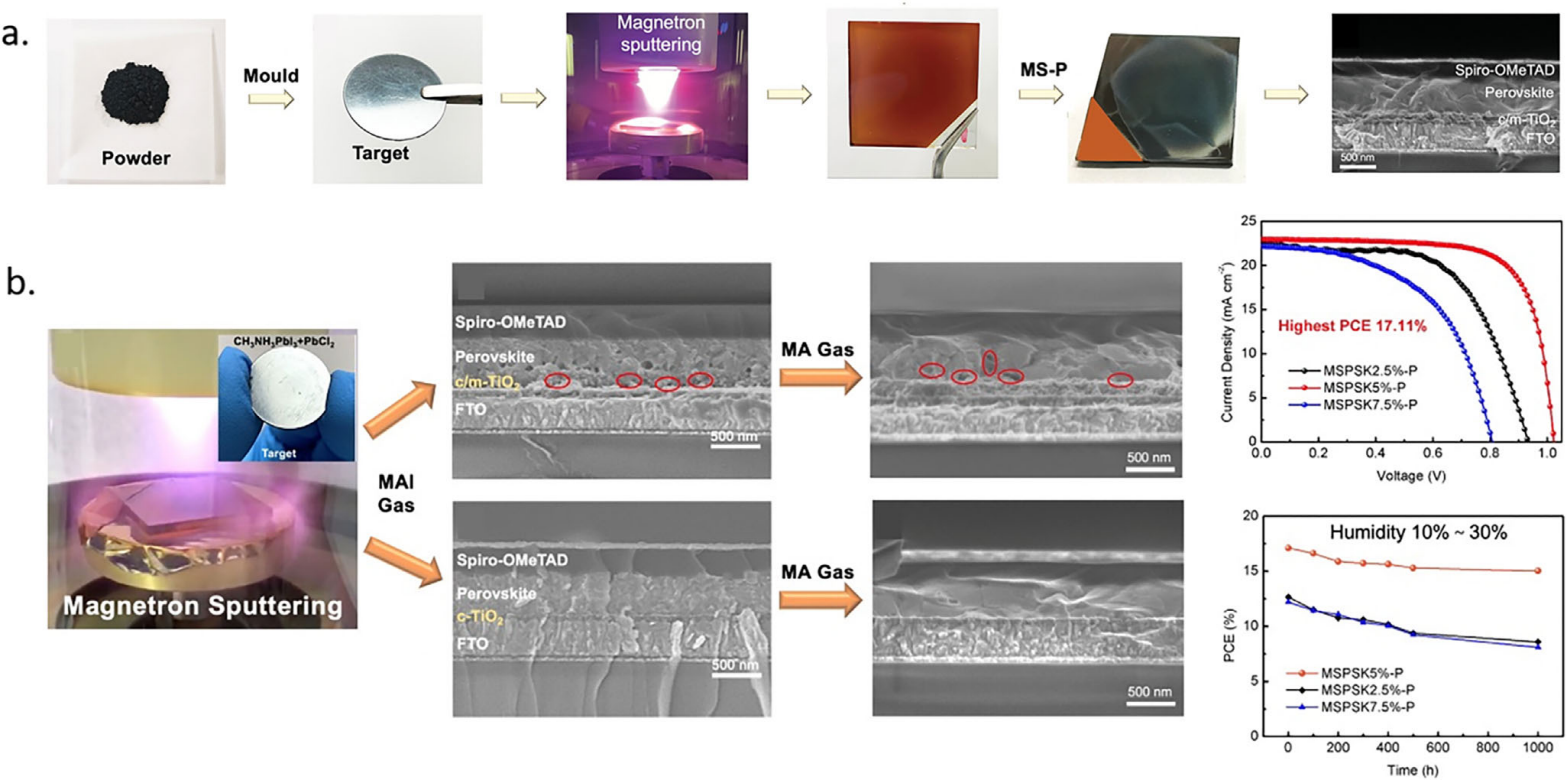
a) The steps of fabrication of PSCs via magnetron sputtering from a single target. Reproduced under the Creative Commons license.^[^ ^146^ ^]^ b) the incorporation of PbCl_2_ into the MAPbI_3_ target and subsequent treatment with MAI and MA gases leading to improved performance and device stability. Reproduced with permission. Copyright 2023, American Chemical Society.^[^ ^148^ ^]^

In 2021, Gao et al. prepared sputter targets from mechano‐synthesized MAPbI_3_ powder and deposited films by magnetron sputtering (**Figure** **10**a), followed by a post‐processing treatment of vapor‐assisted annealing in MAI and methylamine (MA) gas to improve the crystal quality of the MAPbI_3_ films.^[^ ^146^, ^147^ ^]^ They found that the sputtering voltage significantly influenced the film quality. When high voltages (800 V) were employed, the films were rough due to large sputtered particles. In contrast, the use of extremely low voltages resulted in slow sputtering and ultimately led to the decomposition of the perovskite. Remarkably, they achieved a deposition rate high enough to create a 500 nm thick perovskite film in just five minutes. However, the resulting film appeared light brown, probably due to an imbalanced stoichiometry. A post‐processing step with MAI and MA vapor was reported to improve the film quality and increase the carrier lifetime significantly. This treatment led to a doubling the short‐circuit current in solar cells made from these films, facilitating a champion PCE of ≈12%. In addition, Cl was introduced to improve the properties of perovskite thin films and device performances, boosting the champion PCE to ≈15% due to larger grain sizes, fewer pinholes, and higher carrier lifetime.

In a follow‐up study in 2022, the same group further refined their sputtering process and reported a procedure where PbCl_2_ was mechanically milled into the MAPbI_3_ target to regulate the crystallization process and improve the quality of perovskite films (Figure 10b).^[^ ^148^ ^]^ The addition of PbCl_2_ led to a reduction in trap density within the perovskite films and a further increase in the PCE to 17.10%. The authors noted that mesoporous TiO_2_ layers were only poorly percolated by sputtered perovskite, resulting in a significant number of unfilled mesoscopic pores. Such suboptimal buried interfaces hindered electron extraction, limiting the PCEs to only ≈4% for untreated films and ≈15% for post‐processed films. By switching to a planar device architecture without mesoporous TiO_2_ layers, they achieved PCEs of ≈16%.

In 2023, the deposition of all functional layers of PSCs through magnetron sputtering was demonstrated.^[^ ^149^, ^151^ ^]^ To achieve this, the organic charge transport layers (CTLs) were replaced with inorganic alternatives, creating a device with the following structure: FTO/NiO_x_/MAPbI_3_/PMMA:PCBM/sp‐SnO_2_/Ag, where all layers were prepared by magnetron sputtering. Recognizing the potential for damage that could be caused by the high‐energy particles during SnO_2_ deposition, a mixed protective buffer layer of PMMA and PCBM was sputtered on top of the perovskite layer. These devices with all sputter‐deposited functional layers reached a commendable PCE of 14.62%. While lower than the 17.43% obtained when the perovskite layer was solution‐processed, this result highlights the potential of all‐sputtered PSCs. The authors emphasized the importance of further development of dense and stable inorganic CTLs to bridge the performance gap. Impressively, the devices demonstrated excellent stability, retaining 93.5% of their initial PCE for 2000 h when stored under nitrogen. Nevertheless, other perovskite compositions, specifically (FAPbI_3_)_0.867_(MAPbBr_3_)_0.133_ and CsPbI_3_, exhibited very poor performance, likely caused by inadequate surface morphology, underscoring the need to optimize the sputtering process for each specific composition.

Recently, deposition of mixed cation and mixed halide perovskites using magnetron sputtering was demonstrated.^[^ ^150^ ^]^ Using an optimized target composition of (FA_1‐x_MA_x_)Pb(I_1‐x_Br_x_)_3_ with x = 0.133 and a 30 min post‐deposition annealing procedure, the authors achieved dense and pin‐hole free perovskite films, yielding PCEs of up to 20.1% when incorporated into an n‐i‐p‐type solar cell. While MABr appeared essential for promoting proper crystal formation, analysis revealed that it evaporated from the film during annealing, leaving a FAPbI_3_‐based perovskite behind. The impact of the MABr additive on crystal growth was further investigated in follow‐up work by the same group, where they confirmed that x = 0.133 was indeed the optimal MABr content. Here, the authors further noticed an evolution of the sputtering process throughout the duration of the procedure when using a stoichiometric target: The relative amount of organic compounds decreased, while the ratio of inorganic compounds gradually increased over the course of an 8 minutes deposition process.^[^ ^151^ ^]^ These reports highlight the high potential of sputtering for the deposition of highly efficient devices, demonstrating its capability to rival traditional solution‐based fabrication methods.

#### 2.3.4 Direct Sputtering of Inorganic Perovskites

A number of reports focused on the deposition of wide bandgap perovskite compositions like CsPbBr_3_ and CsPbCl_3_, as well as double perovskite such as Cs_3_Bi_2_I_9_. These materials are attractive for applications not limited to photovoltaics, including photodetectors and radiation dosimeters. In 2020, Borri et al. successfully deposited a homogeneous CsPbBr_3_ film with uniform surface morphology directly by sputtering a CsPbBr_3_ target.^[^ ^152^ ^]^ The targets were prepared by ball‐milling and pressing equimolar mixtures of CsBr and PbBr_2_ powders. The sputtering process was carried out at room temperature, and the resulting 70 nm thin film did not require any subsequent postprocessing. XRD measurements revealed the presence of both orthorhombic CsPbBr_3_ and CsPb_2_Br_5_ phases. In addition, XPS measurements indicated an excess of Cs (≈45%) and a deficiency of Br (≈15%), compared to the ideal composition, which was attributed to the different sputtering rates of Cs and Br.^[^ ^145^ ^]^

In 2021, Xu et al. demonstrated the impressive capability of sputtering to produce large‐area, phase‐pure, and ultra‐smooth CsPbBr_3_ films on glass substrates with a size of 10 × 10 cm^2^.^[^ ^153^ ^]^ They highlighted the crucial role of substrate temperature in achieving high‐quality films. A stoichiometric target resulted in Cs‐deficient films due to the higher sputtering rate of Pb; hence, the target composition was adjusted to CsBr/PbBr_2_ = 1.5:1 to reach the desired stoichiometry in the deposited films. Temperature‐dependent photoluminescence measurements and femtosecond transient absorption spectra confirmed the absence of sub‐band defect levels, suggesting that these films are suitable for application in optoelectronics. In 2023, Morello et al. explored the amplified spontaneous emission (ASE) in phase‐pure orthorhombic CsPbBr_3_.^[^ ^154^ ^]^ 500 nm thick films were deposited by single‐step RF‐magnetron sputtering from a target with 1:1 and 1.15:1 CsBr/PbBr_2_ compositions. Both samples showed ASE over a broad range of temperatures from 10 K up to 270 K and above, with the CsBr‐rich films exhibiting a fourfold increase in ASE. A strong temperature dependence of the ASE threshold was evident from these measurements, with the best performance being recorded at ≈50 K. The reduced ASE at higher temperatures might be caused by thermally induced optical quenching. The report further highlights that, at higher temperatures, progressive free exciton dissociation favors higher carrier mobility, which increases trapping at defect states and reduces emission. The authors concluded that these efforts clear the path for the next generation of gain media with controllable optical features by exploiting the flexibility of sputtering deposition.

In 2022, Bruzzi et al. explored the potential of using sputtered wide‐bandgap perovskite CsPbCl_3_ for application as wearable dosimeters in clinical radiotherapy.^[^ ^155^ ^−^ ^157^ ^]^ Films with a thickness of ≈1 µm were grown by magnetron sputtering on plastic substrates pre‐patterned with Cu electrode arrays. The sputtering target was prepared by hot pressing the ball‐milled CsPbCl_3_ powders obtained from the equimolar mixture of CsCl and PbCl_2_, leading to thin films with a bandgap of 3 eV. Later, the same group demonstrated flexible UV photodetectors processed via a single‐step magnetron sputtering growth of polycrystalline CsPbCl_3_ films.^[^ ^158^ ^]^ Perovskite layers were deposited onto plastic substrates with interdigitated contacts, similar to their previous work. Measurements of the photoconductive response to pulsed UV light in the 0.1–100 Hz frequency range (10–500 W m^−2^) showed good signal stability, fast response to transient signals, and a signal‐to‐noise ratio of ≈100. The moderately low detectivity of ≈10^6^ Jones was attributed to the low responsivity caused by the sample geometry, as well as the non‐negligible dark current.

Caporali et al. reported the fabrication of 500 nm thick, lead‐free, inorganic Cs_3_Bi_2_I_9_ perovskite films using RF sputtering from a single target containing CsI and BiI_3_.^[^ ^159^ ^]^ To obtain a stoichiometric film, the target composition was optimized to compromise a 20% w/w excess of CsI. The effect of substrate temperature during deposition, as well as post‐growth annealing, was investigated. Annealing at 150 °C improved the uniformity of the perovskite film, while treatment at 300 °C inflicted damage. Similarly, deposition on a preheated substrate at 150 °C also yielded a higher film crystallinity. The as‐deposited films had a thin surface layer of bismuth oxide that disappeared after the post‐deposition thermal treatment. The best results were obtained by combining substrate heating at 150 °C during deposition with post‐deposition annealing at the same temperature, leading to improved crystallinity and more uniform morphology.

#### 2.3.5 Conclusion on Magnetron Sputter Deposition of Metal‐Halide Perovskites

Magnetron sputter deposition is valued throughout the industry as a scalable deposition method that is capable of producing films with great precision and of the highest quality. Motivated partly by the industrial appeal, the sputter deposition of MHPs has achieved respectable advancements in a short period. While the relatively complex instrumentation and process control might impose a barrier for new groups to explore this technique, the field will benefit when greater efforts are invested in optimizing this intriguing method. However, to catch up to more mature techniques such as TE, a number of challenges need to be addressed.

An intrinsic aspect of sputter deposition is a comparably high background pressure introduced by the inert mediator gas. The resulting particle collisions in the vapor phase have different effects on compounds depending on their mass. Most reports find a deficiency of A‐site cations relative to B‐site cations, most commonly compensated with post‐treatments or an excess of compounds containing the A‐site cation in the sputtering target. Specialized instrumentation, as available to some groups, can seemingly replace such less desirable measures to control film stoichiometry. Alternative approaches could draw inspiration from TE, where sequential and co‐deposition offer improved control and routinely yield the best outcomes. Once the precise control over film stoichiometry is achieved, the field will likely need to turn to additives. While the benefits of organo‐halide and metal‐halide salts were already demonstrated, it remains unclear if organic molecules of various sizes can be incorporated in targets and sputtered films to offer an additional dimension to control material properties such as crystal growth dynamics, grain sizes and orientations, crystal phases, defect passivation, and film stability. If these modifications are adopted successfully, magnetron sputtering is a highly promising approach for the high‐throughput industrial‐scale roll‐to‐roll production of flexible photovoltaics due to the potentially high deposition rates and the relatively facile scalability of sputter targets. **Table** **2** provides a summary of the herein discussed reports of magnetron sputtering processed PSCs.

**Table 2:** Summary of PSCs based on magnetron sputter deposition.

Perovskite	Small area photovoltaic parameters				Stability	Area [cm^2^]	Procedure	Architecture	Year	Refs.
	*V*_oc_ [V]	*J*_sc_ [mA cm^−2^]	FF [%]	PCE [%]						
MAPbI_3_	1.04	20.2	66.9	14.1	–	0.09	Reactive sputtering of PbO from Pb target, conversion with MAI solution	FTO/ nanocrystalline rutile titania (NRT)/Perovskite/Spiro‐MeOTAD/Au	2017	[138]
MAPbI_3_	0.813	3.61	62	1.84	–	–	Sputtering of PbI_2_, conversion with MAI vapor	FTO/TiO_2_/Perovskite /Spiro‐OMeTAD/Au	2017	[141]
MAPbI_3_	0.86	22.1	53	10.2	–	0.075	Sputtering of PbO, conversion with MAI vapor	FTO/c‐TiO_2_/Perovskite /Spiro‐MeOTAD/Au	2020	[139]
MAPb(ICl)_3_	0.97	22.53	71	15.52	80% PCE after 1000 h storage in N_2_	0.060	Sputtering of MAPb(ICl)_3_, post treatment with MAI and MA vapors	FTO/TiO_2_/Perovskite /Spiro‐OMeTAD/Au	2021	[146]
MAPb(ICl)_3_	1.02	22.97	73	17.1	85% PCE after 1000 h storage in N_2_	0.080	Sputtering of MAPb(ICl)_3_, post treatment with MAI and MA vapors	FTO/TiO_2_/Perovskite /Spiro‐OMeTAD/Au	2022	[148]
MAPbI_3_	0.97	25.1	74	12.2	–	0.075	Sputtering of PbI_2_, post treatment with I_2_ vapor, DMSO vapor, conversion with MAI powder, MA vapor annealing	FTO/TiO_2_/Perovskite /Spiro‐OMeTAD/Au	2023	[142]
MAPb(IBr)_3_	0.89	7.6	61	4.1	–	0.075	Sputtering of PbI_2_, conversion with MAI powder, ion exchange in MABr solution	FTO/TiO_2_/Perovskite /Spiro‐OMeTAD/Au	2023	[143]
MAPb(ICl)_3_	0.91	23.01	69	14.62	93.5% PCE after 2000 h storage in N_2_	0.060	Sputtering of MAPb(ICl)_3_, post treatment with MAI and MA vapors. All functional layers via magnetron sputter deposition	FTO/NiOx/Perovskite /PMMA:PCBM/SnO_2_/Ag	2023	[149]
FAMAPb(IBr)_3_	1.15	24.59	71	20.1	87% after 600 h in AM 1.5G illumination and N_2_	0.060	Sputtering of MAFAPb(IBr)_3_, MABr released during thermal annealing	FTO/TiO_2_/Perovskite /Spiro‐OMeTAD/Au	2024	[150]

### 2.4 Chemical Vapor Deposition

#### 2.4.1 Mechanism and Classification of Solvent‐Free CVD

**Figure 11:**
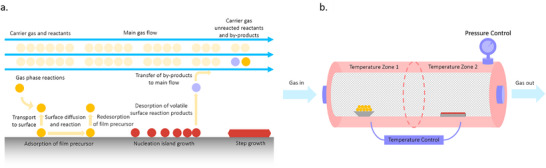
a) Illustration of the steps involved in a CVD process. b) Schematic diagram of a CVD setup. Reproduced with permission. Copyright 2019, Multidisciplinary Digital Publishing Institute.^[^ ^160^ ^]^

Film fabrication through CVD involves a series of chemical reactions in the vapor phase, leading to the deposition of a solid material on a substrate. CVD is a mature thin film deposition technique that is routinely used to prepare high‐quality uniform coatings. The CVD process typically consists of four main steps: i) generation of gaseous reagents, ii) transport of reactants, iii) chemical reaction, and iv) removal of by‐products, as illustrated in **Figure** **11**a. The temperature and gas pressure within the CVD reactor are important process parameters that influence the rate of gas diffusion and the overall deposition process. By carefully controlling these parameters, CVD offers the advantages of precise composition control, excellent film coverage, the ability to coat large area samples and multiple substrates simultaneously, and the capability to coat even samples with irregular surface morphologies conformally.^[^ ^160^ ^]^

Figure 11b displays the schematic of a typical tubular CVD furnace, which usually comprises distinct heating zones for materials and substrates. The process pressure is controlled by balancing the evacuation rate through a gated vacuum pump and the carrier gas inflow. This pressure control is crucial for regulating the reaction rate and, ultimately, the film properties. The pressure levels applied during deposition are commonly used to classify CVD processes into distinct categories: ambient pressure CVD, low‐pressure CVD, and ultra‐high vacuum CVD. The following sections provide an overview of the current progress in CVD‐based deposition of MHPs. We will examine specific processes used to achieve various compositions, pointing out key aspects such as the reaction pathway and temperature control method. Finally, we will discuss the target applications of CVD‐processed perovskites.

#### 2.4.2 Compositional Tuning of Organic–Inorganic Pb‐Based 3D Perovskites

The large difference in the sublimation temperatures between organic salts and lead halides imposes a technical challenge to the CVD of organic‐inorganic hybrid perovskites in a one‐step procedure. Consequently, only a few reports are based on one‐step CVD.^[^ ^160^, ^161^ ^]^ Most works rely on a two‐step approach that starts with the deposition of a lead halide (PbX_2_) film, followed by treatment with an organic salt vapor within the CVD furnace to convert the PbX_2_ layer into perovskite. The deposition technique of inorganic salts in the first step can be CVD, TE, or another solvent‐free method to obtain films with good uniformity. The formation mechanism of the sequential deposition in CVD is similar in principle to the sequential TE. The difference lies in the annealing process, which, in the case of CVD, occurs simultaneously with the deposition of the organic salt, making it an in‐situ process. In contrast, sequential TE typically requires a separate post‐annealing step to complete the perovskite formation.

**Figure 12:**
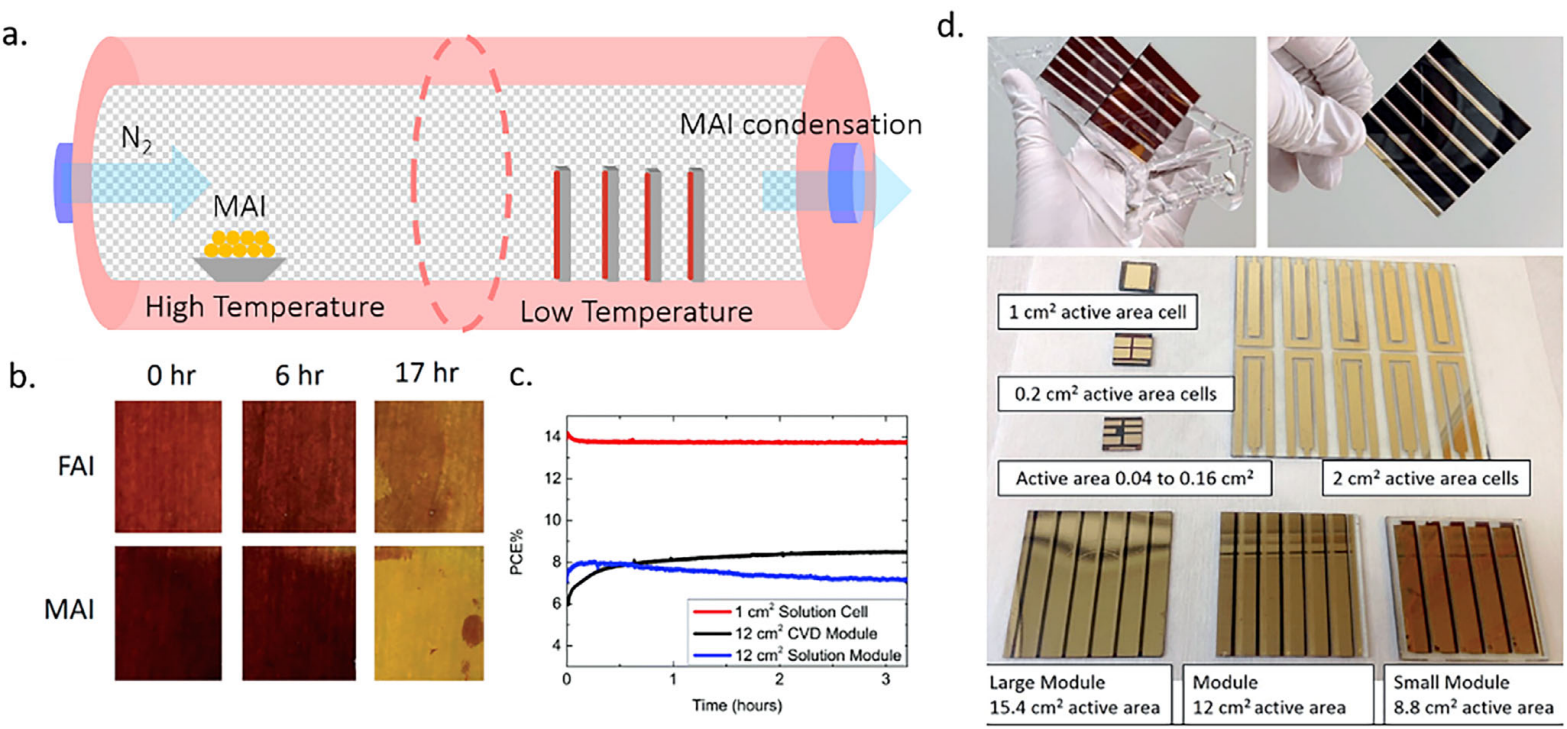
a) Schematic of CVD deposition of MAI in two zones Reproduced with permission. Copyright 2014, Royal Society of Chemistry.^[^ ^162^ ^]^ b) Comparison of the stability of CVD‐grown MAPbI_3_ and FAPbI_3_ films. Reproduced with permission. Reproduced under the Creative Commons license.^[^ ^165^ ^]^ c) Comparison of steady‐state power tracking for a solution prepared and CVD‐grown cells and modules. d) Photograph of patterned perovskite films, cells, and modules of (c). Reproduced under the Creative Commons license.^[^ ^166^ ^]^

Early research on CVD‐derived perovskites focused mainly on MAPbI_3,_ as reported by Leyden et al. in 2014.^[^ ^162^ ^]^ The deposition was achieved by a sequential process: first, a PbCl_2_ film was deposited on the substrate through TE to provide a more uniform morphology than could be achieved with PbI_2_. Next, the CVD deposition of MAI was performed. As indicated in **Figure** **12**a, the MAI powder and substrates were positioned in two separate temperature zones to control the sublimation and reaction independently. Smooth MAPbI_3_ films with a thickness of 300 nm and a grain size of ≈0.6 µm were obtained. The initial PbCl_2_ layer determined the thickness of the perovskite films. Surprisingly, substrates that were post‐annealed in air led to a better film quality than those annealed in N_2_. In the same year, Ha et al. reported a different CVD‐based approach for growing lead halide nano‐platelets: they used van der Waals epitaxy to coat on muscovite mica substrates in a vapor transport CVD deposition system.^[^ ^163^ ^]^ A gas‐solid reaction with organo‐halide salts converted the as‐grown platelets to perovskites. The lead halide perovskite nano‐platelets in this work exhibited a high crystal quality and good optical properties.

Interestingly, the stability of CVD‐processed MAPbI_3_ is superior to that of solution‐processed counterparts. Luo et al. found that MAPbI_3_ films fabricated with a double‐zone sequential CVD were more moisture‐resistant when compared to solution‐processed MAPbI_3_ over the same period.^[^ ^164^ ^]^ Despite this improved moisture stability, the poor thermal stability of the MA^+^ cation remained a concern, which motivated researchers to explore perovskites based on the more stable FA^+^ cation. In 2015, Leyden et al. reported the fabrication of FAPbI_3_ perovskite using sequential deposition of PbCl_2_ and FAI.^[^ ^165^ ^]^ The FAPbI_3_ film exhibited better stability than the CVD MAPbI_3_ film (Figure 12b). In a subsequent study from 2016, the authors modified the CVD process for the fabrication of large‐area films by slightly increasing the FAI source temperature. The large‐area films were incorporated into photovoltaic devices that exhibited superior operating stability compared to the solution‐processed equivalents, highlighting the suitability of CVD for the scale‐up of perovskite film fabrication (Figure 12c).

**Figure 13:**
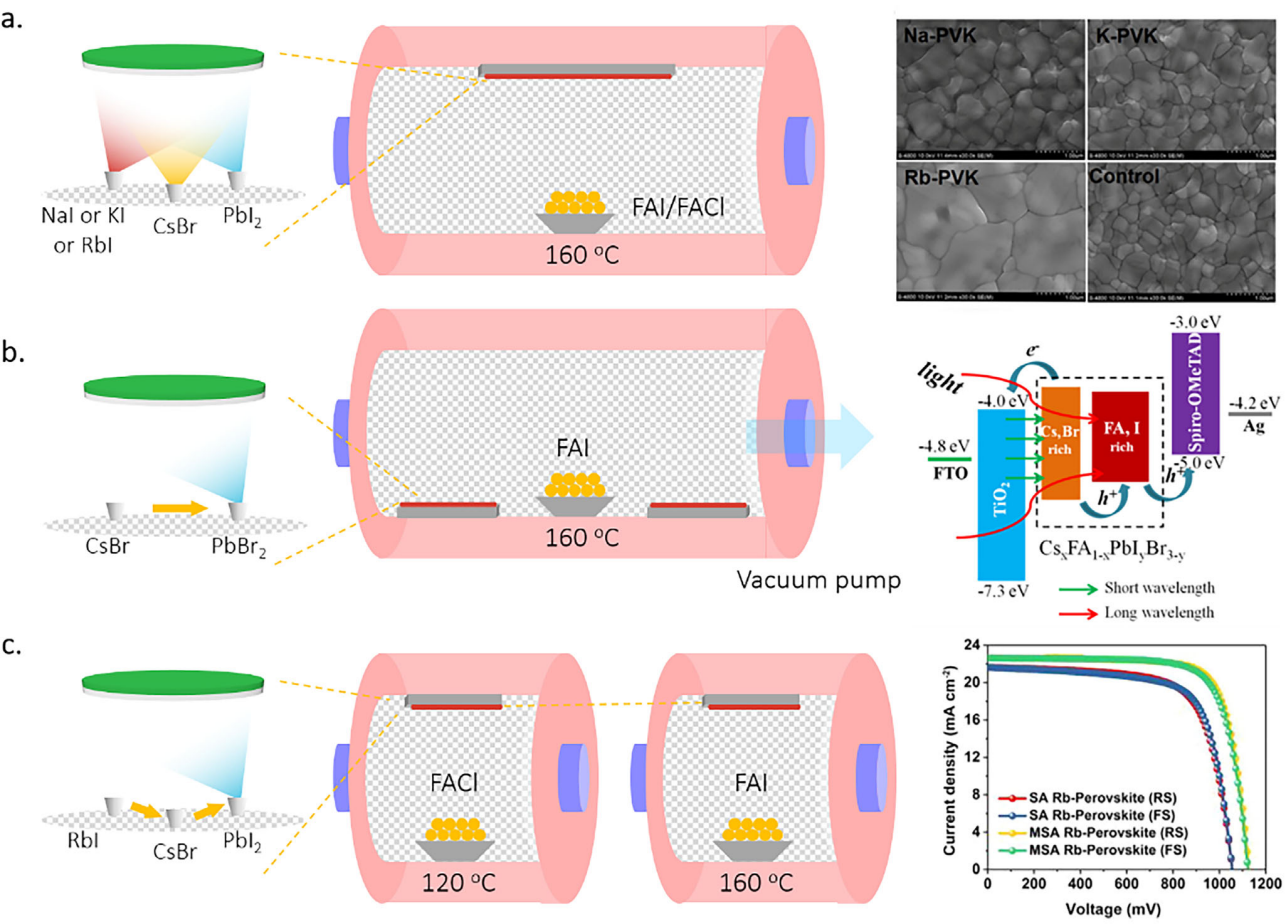
a) Left: Schematic of the addition of sodium, potassium, and rubidium alkali‐metal cations into Cs_0.14_FA_0.86_Pb(Br_y_I_1−y_)_3_ perovskite using a one‐zone sequential CVD method. Right: SEM of perovskite films. Reproduced with permission. Copyright 2021, Wiley‐VCH.^[^ ^172^ ^]^ b) Left: Schematic of the deposition of Cs_x_FA_1‐x_PbI_y_Br_3‐y_ using CVD. Right: energy level diagram of the PSC. Reproduced under the Creative Commons license.^[^ ^169^ ^]^ c) Left: a multistage atmosphere‐assisted process for the deposition of Rb_0.04_^−^Cs_0.14_FA_0.86_Pb(Br_y_I_1−y_)_3_. Right: The *J*–*V* curve of solar cell devices. Reproduced with permission. Copyright 2021, Elsevier.^[^ ^171^ ^]^

As the research focus gradually shifted from MA to the more stable FA‐based perovskites, incorporating two or more different A‐site cations has emerged as a popular approach for enhancing phase stability.^[^ ^167^ ^]^ In 2017, Luo et al. used a single‐zone sequential CVD method to structurally stabilize the α‐phase FAPbI_3_ by incorporating smaller inorganic Cs^+^ cations.^[^ ^168^ ^]^ Using a similar approach, Cheng et al. explored the addition of sodium, potassium, and rubidium alkali‐metal cations into Cs_0.14_FA_0.86_PbI_3‐x_Br_x_ perovskite with the aim of regulating the halogen exchange during the CVD process. This resulted in high‐quality films with large grain sizes and low trap density (**Figure** **13**a). Tong et al. reported that the sequential deposition of CsBr and PbBr_2_ leads to a non‐uniform incorporation of Cs^+^ cations in Cs_0.15_FA_0.85_PbI_2.85_Br_0.15_, resulting in a tailored gradient band gap profile (Figure 13b).^[^ ^169^ ^]^ By adding SrI_2_ into Cs_0.24_FA_0.76_PbI_3−x_Br_x_, Deng et al. found that the conversion rate of PbI_2_ to perovskite was increased, thereby eliminating the PbI_2_ residue at the bottom of the perovskite film.^[^ ^170^ ^]^

In order to process perovskites with more complex compositions and achieve even higher film quality, a wider range of CVD process parameters became subject to systematic investigations. Luo et al. proposed a multistage atmosphere‐assisted process in which RbI, CsBr, and PbI_2_ hybrid films first reacted with FACl vapor at low temperatures to create a RbCsFA_2_Pb(Br_y_Cl_1−y_)_4_ intermediate phase, followed by a second‐step reaction between FAI and the intermediate phase at high temperature to produce the final Rb_0.04_^−^Cs_0.14_FA_0.86_Pb(Br_y_I_1−y_)_3_ composition (Figure 13c).^[^ ^171^ ^]^ Compared to the direct reaction with FAI, the introduction of the intermediate phase improved the ion exchange rate and promoted the full conversion of the PbI_2_ and FAI precursors into the desired perovskite.

#### 2.4.3 Impact of Substrate and Carrier Gas on the Deposition of Perovskites by CVD

**Figure 14:**
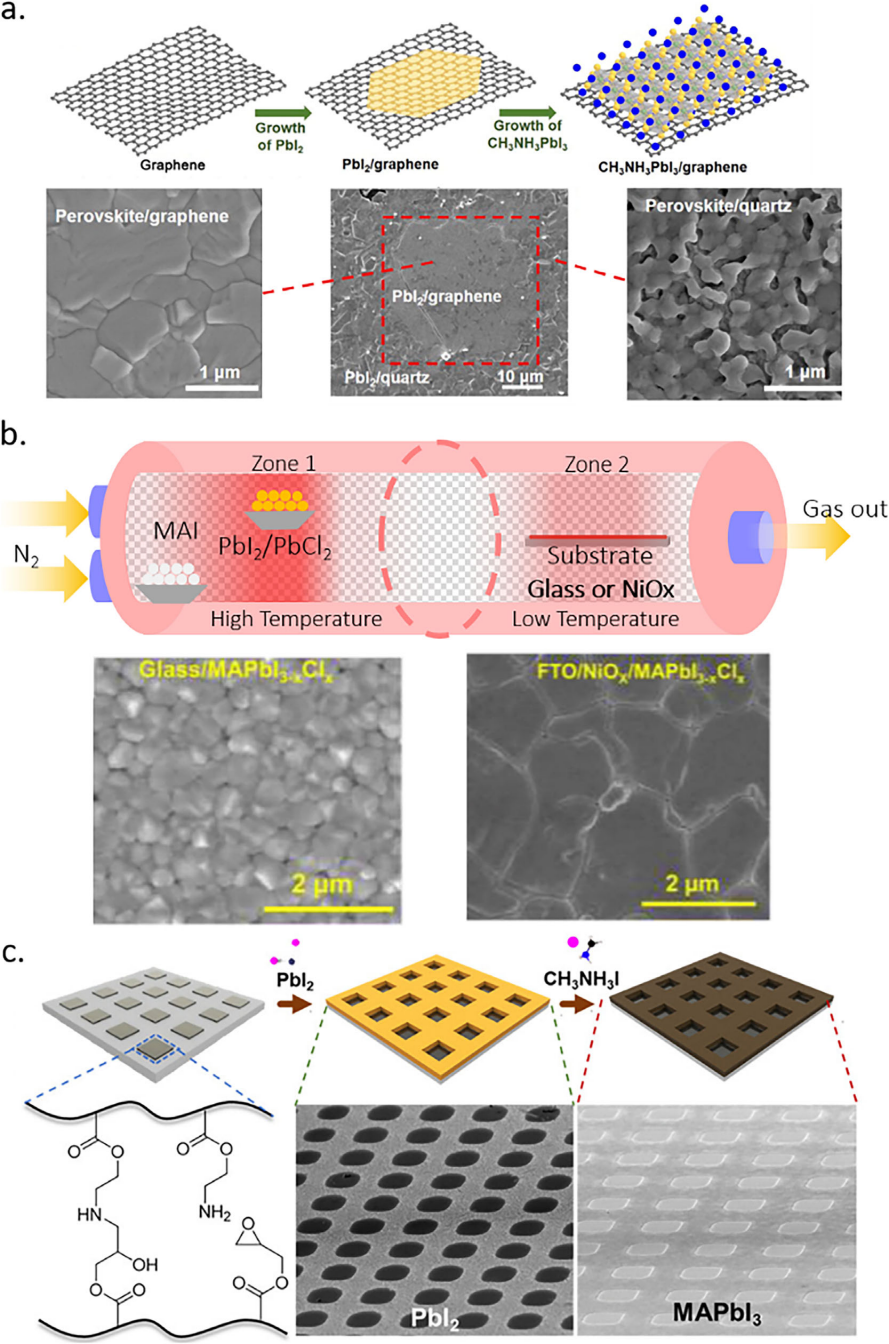
The impact of substrate on the growth and microstructure of CVD‐deposited perovskite layers comparing a) graphene with quartz. Reproduced with permission. Copyright 2019, Elsevier.^[^ ^173^ ^]^ b) NiO_x_, Reproduced with permission. Copyright 2020, Elsevier.^[^ ^174^ ^]^ c) Site‐selective deposition of MAPbI_3_ by CVD. Copyright 2019, American Chemical Society.^[^ ^176^ ^]^

The properties of CVD‐processed MAPbI_3_ are also strongly dependent on the choice of substrate materials. Smooth, flat substrates tend to promote the growth of perovskite with flat, large grains. For example, Xia et al. found that MAPbI_3_ forms dense and smooth films with a grain size of 2 µm when grown on graphene.^[^ ^173^ ^]^ In contrast, the average grain size in films deposited on quartz substrates was only about 150 nm (**Figure** **14**a). Pammi et al. compared the growth of MAPbI_3_ on glass and NiOx substrates.^[^ ^174^ ^]^ They found that NiOx promotes a flat surface and large grains (Figure 14b). Furthermore, the perovskite growth rate was significantly faster (13 nm min^−1^) on NiOx than on glass substrates (9.16 nm min^−1^). The accelerated growth rate originates from surface reactions between precursor molecules and active sites on the NiOx surface. The authors inferred that a high growth rate can be obtained when the substrate has a high surface energy (low contact angle), high roughness, and is more crystalline.^[^ ^175^ ^]^

Based on the idea of controlling perovskite growth by substrate regulation, Geemin et al. exploited the phenomenon of surface‐dependent growth to develop a method for the site‐selective deposition of MAPbI_3_ perovskite thin films.^[^ ^176^ ^]^ In this method, a photo‐lithographically micro‐patterned polymer template was used as the substrate for a sequential CVD process where PbI_2_ was first deposited and then converted into perovskite (Figure 14c). The difference in surface energy and diffusion between the Si substrate and the polymer regions promoted the patterned deposition of PbI_2_. The pattern selectivity reached 99.7% and was mostly driven by the PbI_2_ growth temperature. The patterned PbI_2_ films preserved their morphology throughout the MAPbI_3_ conversion process during the vapor phase intercalation of MAI. These studies confirm the importance of substrate surface properties in determining the reaction rate of CVD‐processed perovskites and facilitating micro‐patterning.

**Figure 15:**
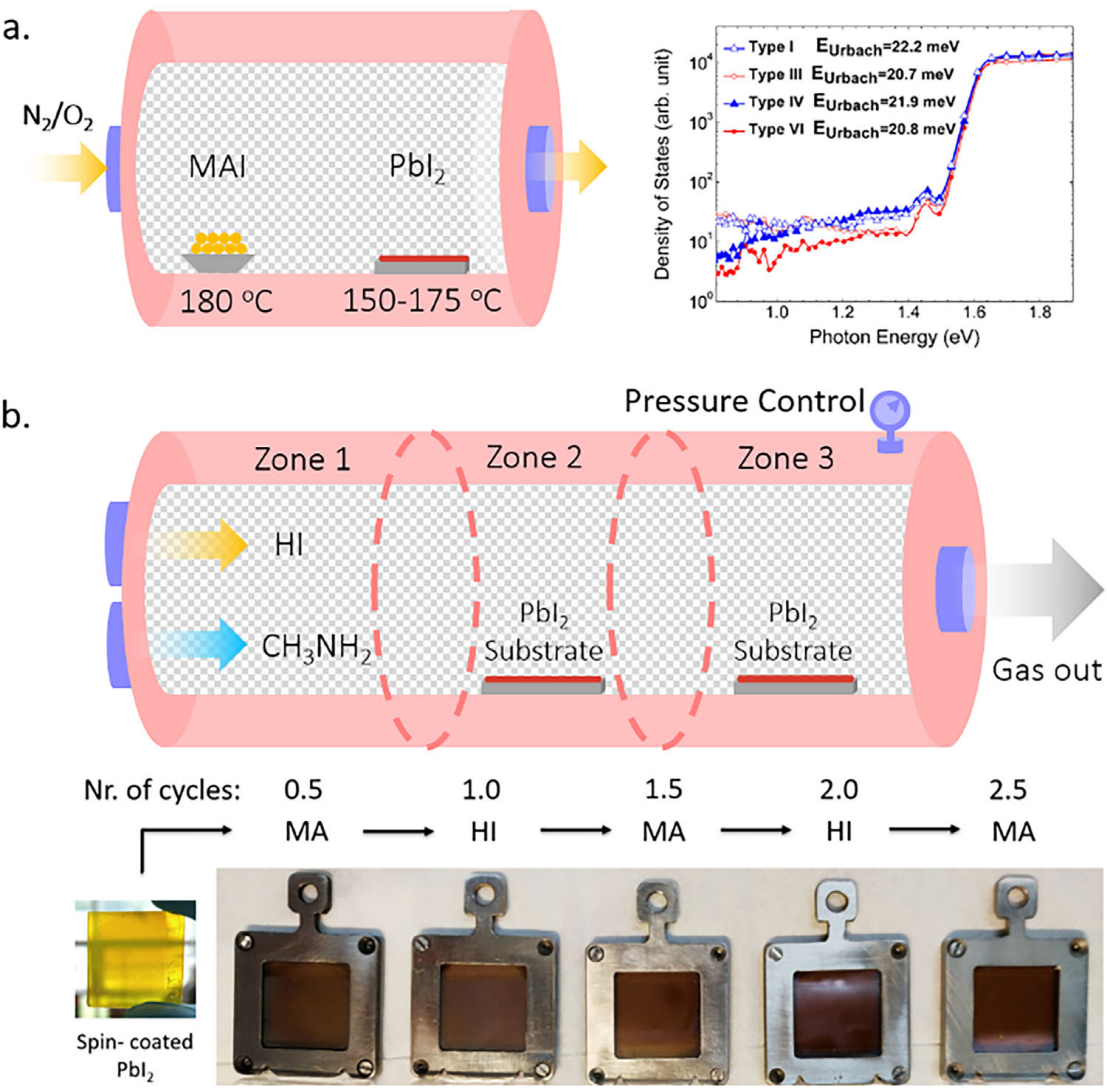
Impact of carrier gases. a) N_2_ and O_2_. Reproduced with permission. Copyright 2016, American Chemical Society.^[^ ^178^ ^]^ b) HI and CH_3_NH_2_ on the properties of CVD‐grown perovskite films. Reproduced under the Creative Commons license.^[^ ^180^ ^]^

The carrier gas fulfills the key functions of carrying the organic vapor to the sample surface and removing by‐products and unreacted reactants. Therefore, its rate, pressure, and temperature need to be carefully adjusted to promote full conversion of perovskites. The most commonly used inert gases are N_2_ and Ar, but some processes adopt air or humid air.^[^ ^177^ ^]^ Ng et al. studied the influence of O_2_ on CVD‐processed MAPbI_3_ by comparing the properties of films obtained in dry air and N_2_, respectively, as the carrier gas.^[^ ^178^ ^]^ They found that the high‐temperature CVD process conducted with N_2_/O_2_ (85%/15%) and using a slow post‐deposition cooling rate can successfully reduce the density of both the shallow and the deep level traps in MAPbI_3_ when compared with a pure N_2_ environment (**Figure** **15**a). Although this work is based on solution‐processed PbI_2_ layers, adding O_2_ to the carrier gas can be extended to solvent‐free CVD. The effect of moisture during CVD is not fully understood. While it was hypothesized that moisture could enhance the crystal quality during the growth of perovskite films,^[^ ^178^ ^]^ Akbulatov et al. proposed that moisture and oxygen cause more defects inside the perovskite layer.^[^ ^179^ ^]^ There is no consensus regarding the influence of the carrier gas and environment on the film quality and defect density, and further investigations are required.

Most efforts employ single organo‐halide salt vapors to convert PbI_2_ into perovskite, and only a few studies explored gas mixtures for this purpose, limiting the options for double organic cation perovskites. Being a technological challenge, controlling and maintaining a stable molar ratio of gas mixtures in CVD also limits the diversity of perovskite compositions that can be processed via CVD. In contrast to the conventional application of MAI vapor in the CVD of MAPbI_3_, Mortan et al. successfully prepared MAPbI_3_ by separately ventilating methylamine and hydrogen iodide gas into the furnace (Figure 15b).^[^ ^180^ ^]^ This method represents an alternative route in industrially established processes, allowing users to avoid the pre‐synthesis of MAI and providing a route towards the fabrication of multi‐organic cation perovskites.

#### 2.4.4 CVD Growth of Inorganic and Lead‐Free Perovskites

The stable structure and facile synthesis of CsPbX_3_ perovskite attracted attention from the scientific community. However, the fabrication of high‐quality CsPbX_3_ thin films via solution processes met several challenges. In addition, the complex formation and transition of different crystal structures can lead to undesired phases that are detrimental to its optoelectronic properties. The phase purity can be affected by other factors, such as the ratio of the precursors, the choice of solvent/antisolvent, and the growth temperature.^[^ ^181^ ^]^ Moreover, the low solubility of cesium‐halides (CsX) in common organic solvents such as DMF and DMSO results in stoichiometric imbalances, which limit scalable crystal growth and can also lead to undesired phases such as Cs_4_PbX_6_ or CsPb_2_X_5_.^[^ ^182^ ^−^ ^184^ ^]^ In addition, solvent residue from solution‐based CsPbX_3_ crystal growth causes persistent electronic defects in the crystal.^[^ ^185^ ^]^

In contrast, CVD was demonstrated as an effective approach to control morphology, minimize surface defects, and thereby improve material quality and stability of CsPbX_3_ films. Moreover, it circumvents the trapping of solvent during the crystal growth process and associated defects. Taking advantage of these aspects, pioneering works have demonstrated the growth of high‐quality all‐inorganic CsPbX_3_ perovskite through CVD. Among published reports on CsPbX_3_, most efforts are based on a one‐step CVD due to the similar sublimation temperatures of the CsX and PbX_2_. CsPbBr_3_ prepared by CVD ranges from 100 nm to micrometer level thickness^[^ ^186^ ^−^ ^190^ ^]^ and from polycrystalline films^[^ ^186^, ^189^ ^]^ to single crystals.^[^ ^187^, ^190^, ^191^ ^]^ Its application includes LEDs, photodetectors, photoelectric lasers,^[^ ^192^ ^]^ and FETs.^[^ ^187^ ^]^

**Figure 16:**
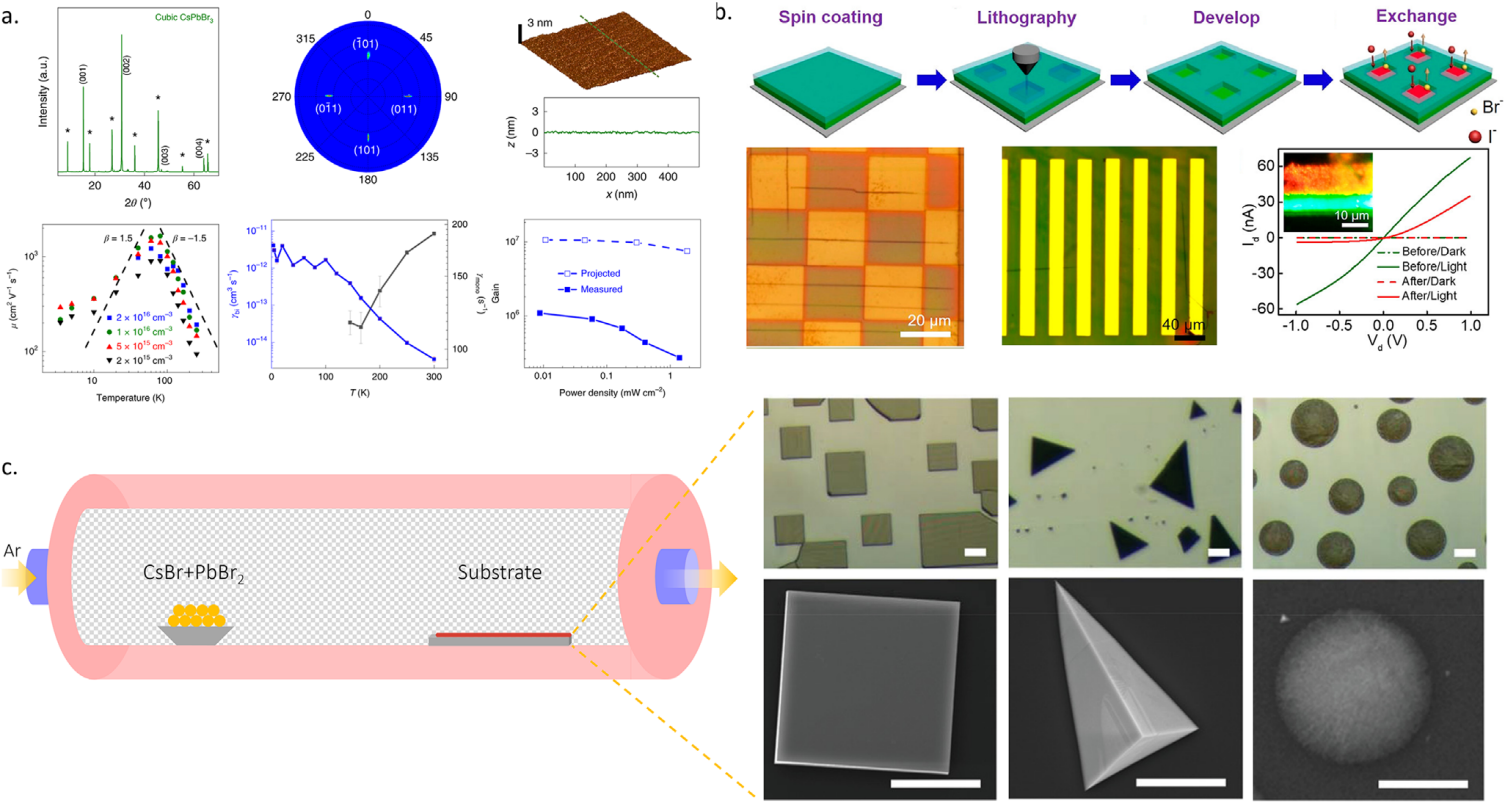
a) The crystal structure, roughness, mobility, recombination coefficient, and photocurrent gain of CVD grown CsPbBr_3_ single‐crystal. Copyright 2020, Springer Nature.^[^ ^190^ ^]^ b) CsPbBr_3_–CsPbI_3_ single‐crystal heterostructure arrays made from CVD. Reproduced under the Creative Commons license.^[^ ^188^ ^]^ c) Different CsPbBr_3_ micro‐morphologies obtained by tuning the cooling rates and growth times and deposition temperatures. Reproduced with permission. Copyright 2018, Wiley‐VCH.^[^ ^199^ ^]^

Bao et al. investigated the effect of temperature on the dynamics of CsPbBr_3_ formation during CVD.^[^ ^193^ ^]^ The authors formulated corresponding equations to relate the inflow rates, desorption coefficients, and concentrations of reactants on the substrates. They concluded that the deposition temperature should be chosen to balance the reaction and condensation rates. Wang et al. demonstrated the CVD of highly oriented single‐crystal CsPbBr_3_ thin films that grew along the (001) plane and had an ultra‐smooth surface with a root mean square roughness of 0.19 nm (**Figure** **16**a).^[^ ^190^ ^]^ The films also featured excellent electrical properties with a high room temperature Hall mobility of 118 cm^2^ V^−1^ s^−1^ and exceeding 2000 cm^2^ V^−1^ s^−1^ at around 80 K, an ultralow bimolecular recombination coefficient of 3.5 × 10^−15^ cm^3^ s^−1^, and a photocurrent gain over 10^6^_._ Xu et al. incorporated these single crystals into UV photodetectors with good performance, high moisture and oxygen stability over twenty‐one months, and good thermal stability.^[^ ^191^ ^]^

Wang et al. explored more complex structural designs based on CVD‐grown single‐crystal films.^[^ ^188^ ^]^ By combining electron beam lithography with site‐selective anion exchange, the authors produced CsPbBr_3_–CsPbI_3_ heterostructure arrays. This achievement represents a significant step towards integrating high‐quality CVD films into electronic devices (Figure 16b). A challenge in CVD growth of perovskites is achieving precise control over film thickness, particularly at the nanoscale. In most reported works, the thickness of single‐crystal films is in the micrometer range. Attempts to grow thinner films in the nanometer range resulted in either polycrystalline or micro‐structured films with discontinuities or uneven surfaces. Hence, the controlled preparation of nanoscale single‐crystal thin films remains to be demonstrated.

It was shown that the cooling rate and growth time can be finely tuned in CVD to obtain various CsPbBr_3_ micro‐morphologies with different phases, including microplates (cubic phase), pyramids (monoclinic phase), and microspheres (monoclinic phase). Moreover, by tuning the deposition temperature, different microstructures such as microwires, microplates, microrods, and triangular pyramids can be synthesized (Figure 16c) with potential in optoelectronic devices.

Besides lead‐based inorganic perovskites, a series of inorganic lead‐free perovskites have been synthesized through the replacement of PbX_2_, including Cs_3_Sb_2_X_9_,^[^ ^194^, ^195^ ^]^ Cs_2_TeI_6_,^[^ ^196^ ^]^ Cs_2_SnI_6_,^[^ ^197^ ^]^ and CsSnI_3_.^[^ ^198^ ^]^ However, these efforts are still in their infancy, and the crystal growth dynamics and suitable application fields for these materials and nanostructures are subject to ongoing investigations.

#### 2.4.5 Modification of CVD Facilities

**Figure 17:**
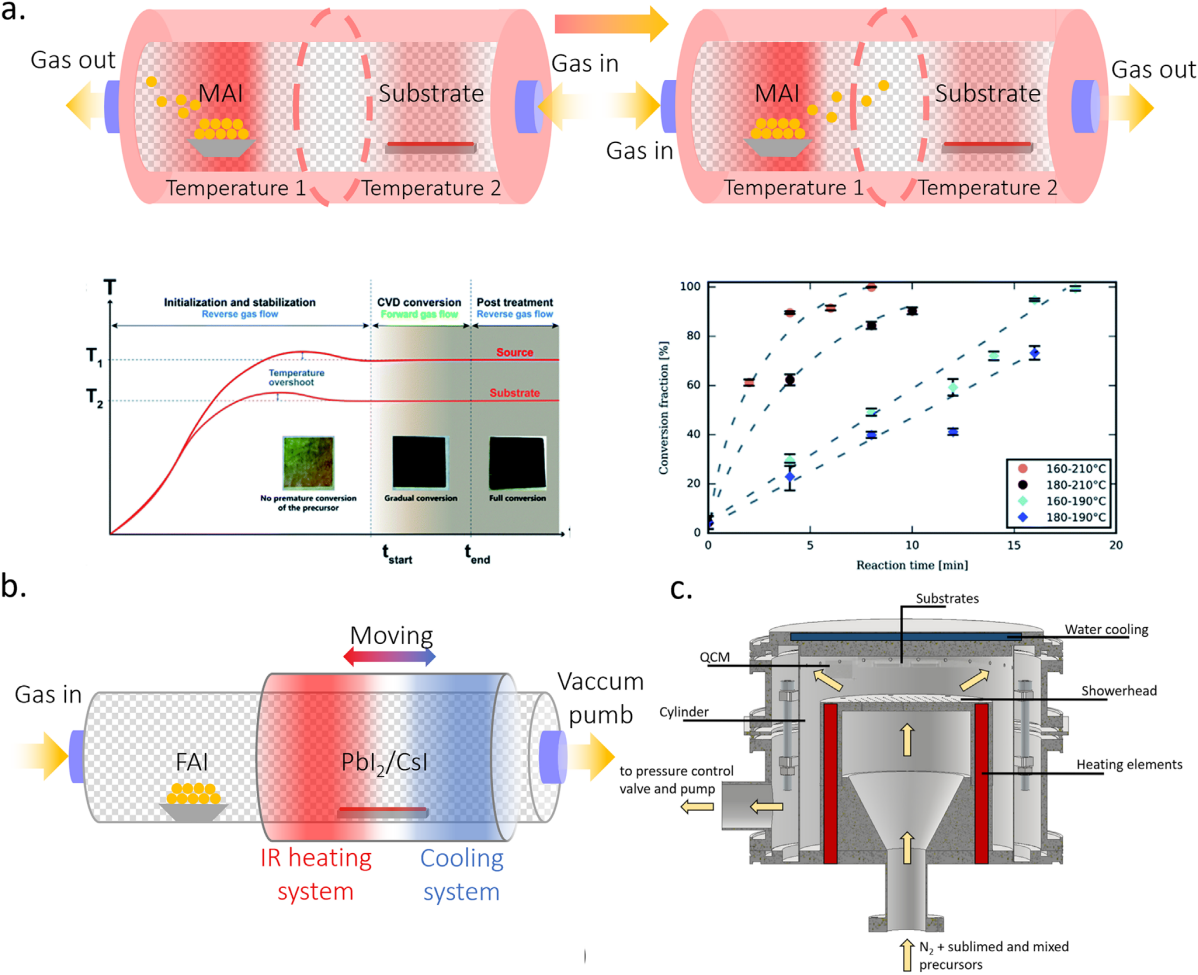
a) Modified CVD facilities with the possibility of switching the pumping direction and correspondingly changing the organic vapor flow direction, enabling the study of the conversion kinetics by CVD. Reproduced with permission. Copyright 2020, Royal Society of Chemistry.^[^ ^200^ ^]^ b) CVD with an integrated movable IR heating and cooling system. Reproduced with permission. Copyright 2020, Royal Society of Chemistry.^[^ ^201^ ^]^ c) Alternative design for a CVD system with a showerhead using a vertical chamber. Reproduced with permission. Copyright 2021, Springer Nature.^[^ ^186^ ^]^

The crystalline quality of perovskite films relies heavily on the design of the CVD equipment. Various modified CVD techniques have emerged, with similar underlying principles as introduced above, i.e., the sublimation or vaporization of precursors and the transport of the vapor to the substrate. A modified pumping system was introduced to regulate the transport of precursor gas and reduce side reactions. Recently, Thierry et al. developed an isothermal CVD process for perovskite growth based on a dual‐direction pumping system, in which the detrimental effect of the sublimated FAI vapor during the ramping and cooling process can be avoided.^[^ ^200^ ^]^ By switching the pumping direction and correspondingly changing the organic vapor flow direction, the reaction kinetics between the vapor and solid has been systematically studied (**Figure** **17**a). In this case, each end of the furnace tube can be connected to the carrier gas supply or vacuum pump, enabling gas circulation in the desired direction. Before reaching the stable temperature, the organic vapor was purged out directly with a carrier gas without passing by the PbI_2_‐coated substrate. By switching the vapor gas flow direction, the deposition of organic halide and conversion to perovskite were initiated. Switching the gas flow at a precisely controlled time prevented excess organic vapor supply, which promoted stoichiometric perovskite formation. Another benefit of this approach is the opportunity to study the conversion kinetics of the hybrid CVD for each deposition step (Figure 17a).

The rates of material sublimation and deposition can be rapidly adjusted with a modified temperature‐controlling system, which reduces the formation of by‐products. More specifically, a rapid and precise heating and cooling process can prevent the overshoot during the ramping process and the resulting over‐saturation of organic vapor. Moreover, electronic devices might benefit from shorter vacuum annealing process steps, which could deteriorate the quality of functional layers. Shorter vacuum annealing times are achieved through optimized thermal transfer and annealing times. An alternative approach is to exchange the conventional filament heater with infrared radiation heating. This rapid thermal process has been recently developed by Qiu et al.^[^ ^201^ ^]^ Ultra‐fast ramping and deposition were achieved, and the deposition time was reduced from 2 to 3 h to 10 min, comparable to solution‐based processes. Figure 17b displays the CVD system used for coating high‐quality perovskite films with rapid ramping, efficient annealing, cooling by infrared radiation, and a movable heating element. In this system, the quality of electron transport layers was preserved, and solar cell and module performances were improved accordingly.

Sanders et al. replaced the typical tubular furnace with a vertical chamber comprised of a custom‐built showerhead‐assisted CVD tool (Figure 17c). Compact and uniform perovskite films with grain sizes between 100 and 140 nm were achieved, and the demonstrated LEDs featured a maximum luminance of 125 cd m^−2^. Similarly, Sahli et al. also used a vapor transport deposition system featuring a showerhead.^[^ ^202^ ^]^ This setup physically separated the source of organic‐halide salt vapors from the deposition chamber to avoid the complex on‐site control of the sublimation. The transport of MAI vapor through a showerhead also ensured a spatially homogeneous conversion of PbI_2_ to MAPbI_3_, and homogeneous perovskite layers were deposited on textured silicon substrates. Overall, organic‐inorganic halide perovskites prepared by solvent‐free CVD still show higher trap densities than those derived from hybrid processes, including solution‐processing and CVD, which in turn are still inferior to fully solvent‐processed compounds. This is reflected in the device performance of the most common application, i.e., PSCs, suggesting that the growth of PbI_2_ and the deposition of organo‐halide vapors require further improvement. Upgrades and optimizations of custom‐built CVD tools appear to be a promising route to investigate a wider range of growth conditions.

#### 2.4.6 Photovoltaic Devices Based on CVD Perovskite

Due to the analogous crystal growth mechanisms between CVD and TE techniques, their developmental pathways for PSCs exhibit certain similarities. For instance, two‐step CVD processes have demonstrated superior device performance compared to one‐step approaches, a trend suggesting that CVD methods also require decoupling of precursor deposition, nucleation, and growth stages to simplify the complexity of crystallization control. Furthermore, for perovskite composition suitable for photovoltaic applications (FA, MA, Cs‐cation, and I‐anion), there has been a declining adoption of CVD for fabricating complete bilayer components. Most studies preferentially employ TE or solution‐processed methods for metal halide layer preparation, resulting in a scarcity of high‐efficiency PSCs entirely fabricated via CVD. Consequently, the majority of research has inherited optimization strategies from TE and solution‐processed methodologies, including additive engineering and compositional modulation. We summarize distinctive CVD‐based fabrication strategies for PSCs in **Table** **3**.

**Table 3:** Summary of PSCs fabricated based on CVD method.

Perovskite	small area photovoltaic parameters				Stability	Area	Evaporation Procedure	Architecture	Year	Key Strategy	Refs.
	*V*_oc_ [V]	*J*_sc_ [mA cm^−2^]	FF [%]	PCE [%]							
MAPbI_3_	0.92	19.1	62	10.8	100% of initial PCE after 1100 h in N2 under dark	0.09 cm^2^	TE/CVD sequential	n‐i‐p: FTO/TiO_2_/perovskite/Spiro‐OMeTAD/Au	2014	First CVD‐based PSC	[162]
MAPb(I,Cl)_3_	0.95	15.9	61	9.2	–	–	One‐step CVD	n‐i‐p: FTO/TiO_2_/perovskite/Spiro‐OMeTAD/Au	2015	First one‐step CVD	[161]
FAPbI_3_	1.06	21.7	68	15.6	–	0.09 cm^2^/2 cm^2^ (PCE = 10.4%)/8.8 cm^2^ module (PCE = 9.5%)/12 cm^2^ module (PCE = 9.0%)/15.4 cm^2^ module (PCE = 5.8%)	TE/CVD sequential	n‐i‐p: FTO/TiO_2_/perovskite/Spiro‐OMeTAD/Au	2016	First CVD‐based FAPbI_3_	[166]
Cs_0.15_FA_0.85_PbI_2.85_Br_0.15_	1.06	22.82	75.4	18.22	60% of initial PCE after 1400 h in air	0.09 cm^2^	TE/CVD sequential	n‐i‐p: FTO/TiO_2_/perovskite/Spiro‐OMeTAD/Au	2018	introducing CsBr	[169]
Cs_0.1_FA_0.9_PbI_3_	0.99	22.3	70.2	15.5	90% of initial PCE after 800 h under light illumination in N_2_	0.1 cm^2^/22.4 cm^2^ module (PCE = 12.3%)	TE/CVD sequential	n‐i‐p: ITO/SnO_2_/perovskite/Spiro‐OMeTAD/Au	2020	Using IR heating/cooling system for rapid thermal process	[201]
MAPbI_3_	1.04	18.6	66.8	12.9	/	0.325 cm^2^	CVD sequential	n‐i‐p: FTO/TiO_2_/perovskite/Spiro‐OMeTAD/Au	2020	Using methylamine gas and hydrogen iodide gas to replace MAI	[180]
Cs_0.04_F _0.96_PbI_3_	0.95	17.5	60	10.6	–	0.27 cm^2^	TE/CVD sequential	p‐i‐n: ITO/PTAA/perovskite/PCBM/ZnO‐nanoparticle	2020	developing an isobaric–isothermal process enable fast film growth rate	[200]
PEA_2_MA_n−1_Pb_n_I_3n+1_	1.08	23.75	70.4	18.08	30% of initial PCE after 48 h under 92% RH/ 87% of initial PCE after 30 days under 55% RH	–	TE/CVD sequential	n‐i‐p: FTO/TiO_2_/C_60_/perovskite/Spiro‐OMeTAD/Au	2020	Intoducing PEAI along with MAI	[203]
Cs_0.24_FA_0.76_PbI_3−y_Br_y_	1.021	21.96	18.9	17.66	95% of initial PCE after 40 days under ambient condition	0.16 cm^2^/25 cm^2^ module (PCE = 13.59%)	TE/CVD sequential	n‐i‐p: ITO/SnO_2_/perovskite/Spiro‐OMeTAD/Au	2020	Introducing SrI_2_ into the inorganic halide layer to promote crystallization	[170]
MAPbI_3_	0.927	17	65.4	12.3	–	0.25 cm^2^	TE/CVD sequential	n‐i‐p: ITO/LiF/C_60_/perovskite/Spiro‐OMeTAD/Au	2021	Using showerheat for MAI deposition	[202]
Rb_0.04_^−^Cs_0.14_FA_0.86_Pb(Br_x_I_1−x_)_3_	1.13	22.2	78	19.6	–	0.16 cm^2^	TE/CVD sequential	n‐i‐p: ITO/SnO_2_/perovskite/Spiro‐OMeTAD/Au	2021	introduction of Rb, Cs FACl to modulate crystallization	[172]

#### 2.4.7 Conclusion on Chemical Vapor Deposition

Similar to early TE methods, CVD faces challenges in achieving solvent‐free crystallization control without DMSO/DMF‐like coordination. Sequential deposition of metal halides and organic halides was shown to improve crystal quality for photovoltaics, yet CVD lags behind TE in this field. This gap may stem from differences in vacuum levels, equipment design (e.g., TE's precise rate control via quartz crystal microbalances and shutters), and process dynamics. However, CVD offers unique possibilities, such as growing diverse nanostructures and single crystals, opening opportunities beyond photovoltaics and towards, e.g., photonics. Targeted investigations of CVD‐fabricated perovskites in sophisticated applications, such as photonic sources, gain materials or optical amplifiers, appear highly promising.

### 2.5 Atomic Layer Deposition

Atomic layer deposition (ALD) is a technique that is relevant to research and industrial applications due to its capability to deposit ultra‐thin and conformal films with precise control over thickness, uniformity, and composition.^[^ ^204^ ^]^ ALD's compatibility with large‐area substrates and roll‐to‐roll manufacturing makes it particularly attractive for the scalable production of PSCs.^[^ ^205^ ^]^ However, applying ALD to process metal‐halide perovskites is not trivial, and no PSC with an ALD‐processed active layer has been reported so far.

Early studies focused on preparing precursor layers that were then converted to perovskites. Specifically, ALD‐grown PbS layers were converted first to PbI_2_ and finally to MAPbI_3_ through treatment with I_2_ vapor and MAI solution, respectively.^[^ ^206^ ^]^ In a key advancement, processes were developed that enabled the deposition of metal‐halides, initially PbI_2_ and later PbCl_2_, PbBr_2_, and the mixed‐halide PbX_n_Y_2−n_ (X, Y = Cl, Br, I).^[^ ^206^ ^−^ ^208^ ^]^ While PbI_2_ is readily converted to MAPbI_3_ through MAI vapor treatment, it was not until the development of the ALD process for CsI that metal‐halide perovskites could be fabricated exclusively through this single technique. Weiß et al. found that by depositing PbI_2_ on a layer of CsI, the γ‐CsPbI_3_ forms following a chemical reaction at a substrate temperature of 100 °C.^[^ ^209^ ^]^ This two‐step ALD process was then adapted to accommodate the growth of CsSnI_3_, as reported by the same group shortly after.^[^ ^210^ ^]^

**Figure 18:**
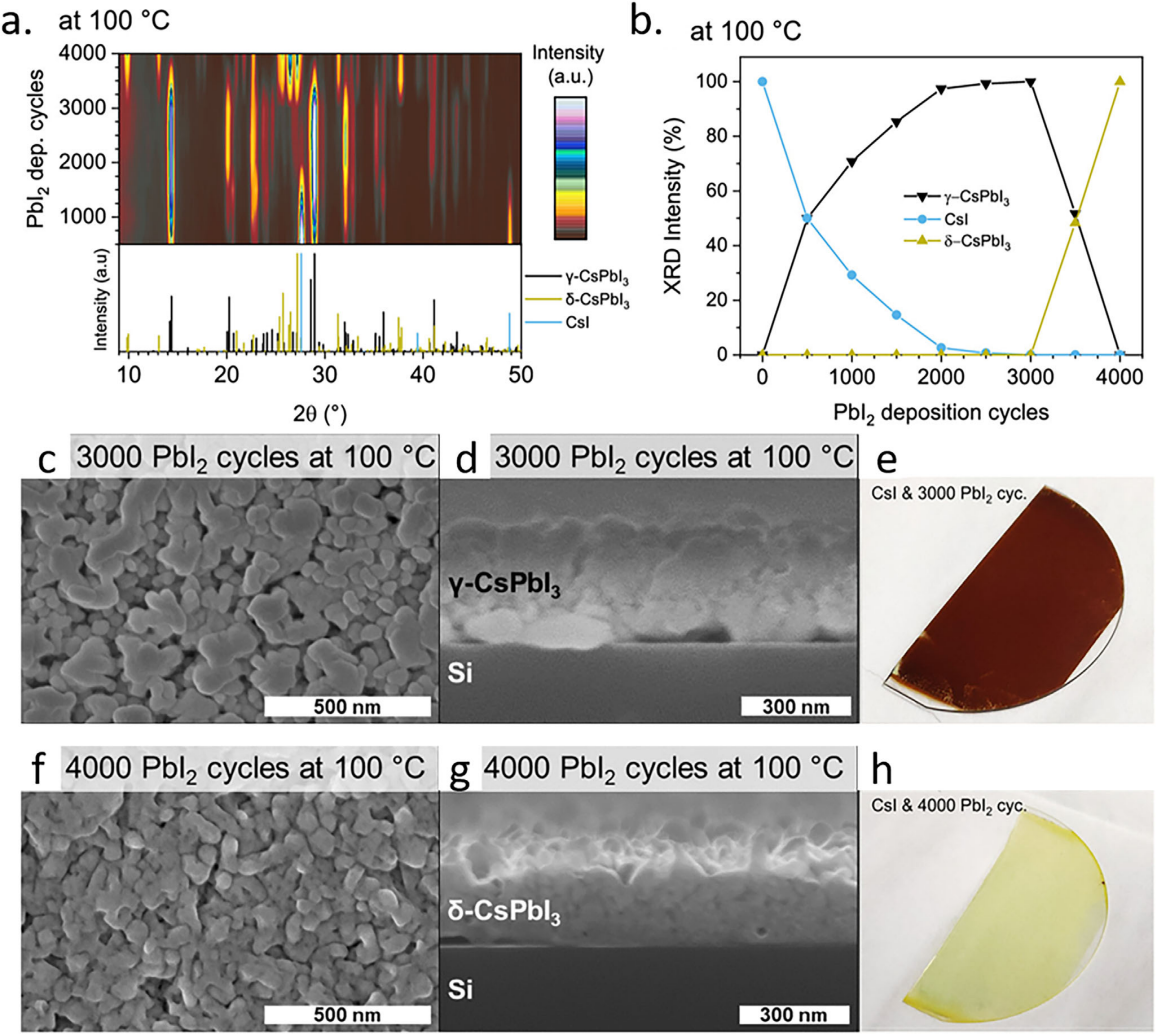
a) XRD patterns of a CsI layer after different numbers of PbI_2_ deposition cycles. b) XRD peak intensities as a function of PbI_2_ deposition cycles indicate the conversion of CsI into γ‐ CsPbI_3_, followed by the sudden transformation into δ‐CsPbI_3_. C) Top‐view SEM image, d) cross‐sectional SEM image, and e. photograph of γ‐ CsPbI_3_. f) Top‐view SEM image, g) cross‐sectional SEM image, and h. photograph of δ‐ CsPbI_3_. Reproduced under the Creative Commons license.^[^ ^209^ ^]^

Despite these encouraging recent breakthroughs that highlight the promise of ALD for processing perovskite films, significant challenges still remain. One problem is that lead‐halide perovskite growth is not possible through a one‐step process, a so‐called supercycle approach, which is a common way for depositing ternary compounds. Instead, two distinct ALD processes are necessary because different materials require different temperatures.^[^ ^209^, ^210^ ^]^ This two‐step method compromises the self‐limiting aspect of ALD and, consequently, the precision in stoichiometry control, potentially leading to film irregularities and small grain sizes. Another issue is the narrow processing window. Although it was reported that excess PbI_2_ is sublimed away from the sample once CsI is fully reacted to obtain CsPbI_3_, adding more PbI_2_ could trigger the conversion of the entire γ‐ CsPbI_3_ layer into the undesirable δ‐CsPbI_3_ phase (**Figure** **18**). In addition, processing temperatures and precursors must be carefully selected to avoid contamination with by‐products. Finally, developing perovskites with more complex compositions, such as mixed‐halide and mixed‐cation, would be desirable because these may enable better crystal formation during ALD deposition. Promoting the application of ALD perovskite films in photovoltaic devices would also be of great interest to achieve outcomes comparable to those of other deposition methods.

### 2.6 Pulsed Laser Deposition

#### 2.6.1 Mechanism and Advantages of PLD

**Figure 19:**
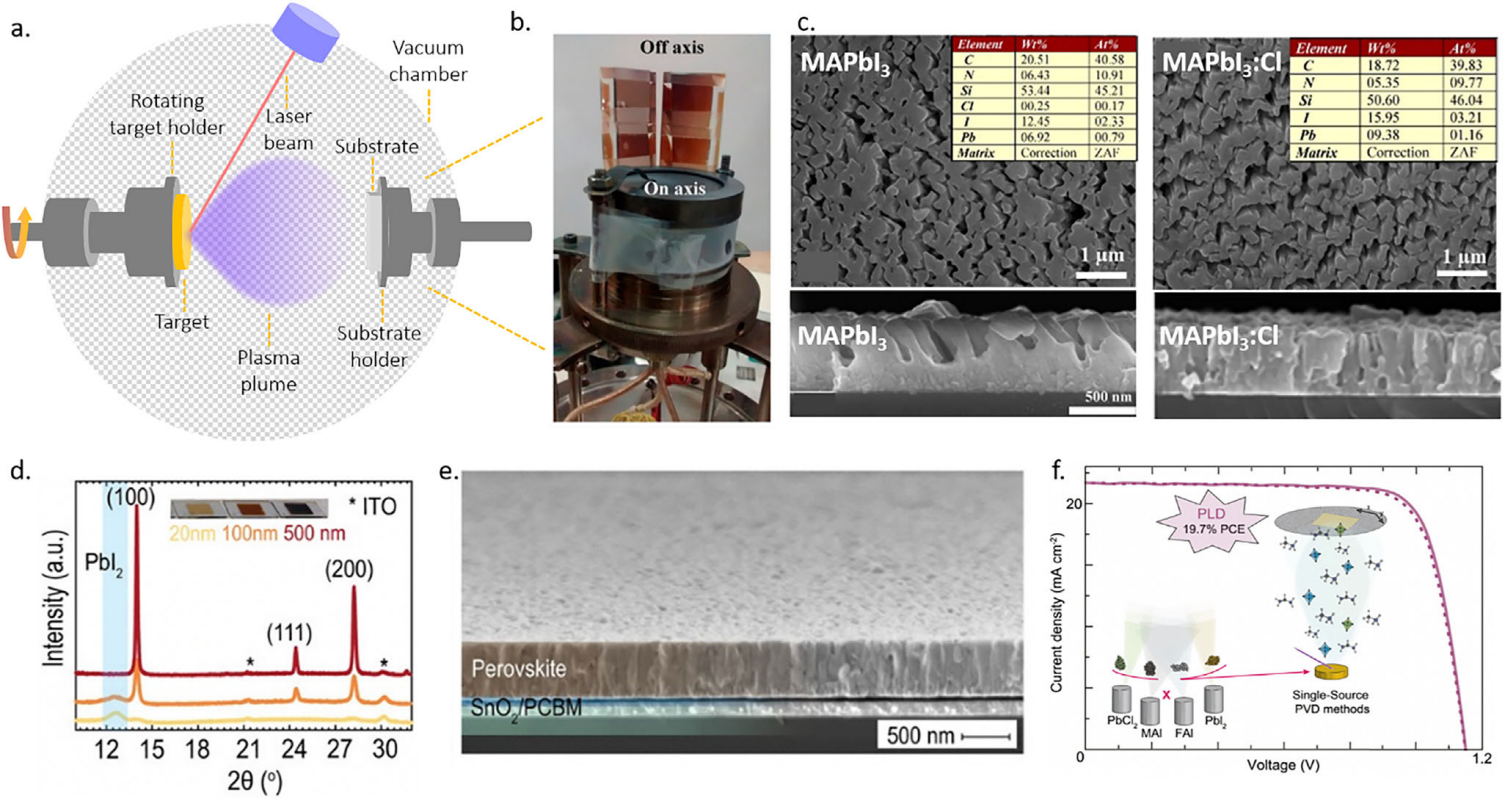
a) A schematic illustration of a PLD setup. b) Photograph of substrate arrangement for “on‐axis” and “off‐axis”. c) The microstructures of the resultant MAPbI_3_ perovskites with and without Cl^−^. Reproduced with permission. Copyright 2015, American Chemical Society^[^ ^211^ ^]^ d) XRD patterns, e) microstructure of PLD deposited MA_1–x_FA_x_PbI_3_ thin films. Reproduced under the Creative Commons license.^[^ ^212^ ^]^ f) *J*–*V* curve of PLD deposited MA_1–x_FA_x_PbI_3_ solar cells. Copyright 2024, Elsevier.^[^ ^213^ ^]^

Pulsed laser deposition (PLD) is a widely used method for depositing high‐quality thin films. PLD is performed inside a vacuum chamber, where high‐power laser pulses melt, evaporate, and ionize material from the surface of a target made of the material one wants to deposit. This process produces a transient, highly luminous plasma plume that contains neutral specimens, ions, electrons, and photons that expand rapidly away from the surface of the target and precipitate on an appropriately positioned substrate (**Figure** **19**).

In essence, PLD comprises three steps, starting from 1) the laser‐target interaction where the electromagnetic energy is converted into electronic excitation and then into thermal and/or mechanical energy that causes ablation, 2) the creation of plasma that contains atoms, molecules, electrons, ions, clusters, particles, and molten globules that expands in three‐dimensions in an isothermal way and 3) precipitation of the ablated materials on the substrate surface. The first two steps occur during the laser pulse incidence, while the last process initiates after the termination of the laser pulse. The nucleation and growth of the crystalline films depend on numerous factors, such as density, energy, degree of ionization, the type of condensing material, substrate temperature, and the substrate's physical and chemical properties. PLD has several benefits over other film deposition methods:

- It utilizes non‐volatile, multi‐component, and multiple targets at a time to deposit layered films or alloys.
- Conventionally, it can accurately transfer the composition of the target to the substrates such that the films obtained possess the exact chemical composition to that of the target.
- A reasonably high deposition rate of about 100 Å min^−1^ can be achieved at moderate laser fluencies.
- Deposition can be carried out at a wide range of pressures, from atmospheric to a few mTorr, in any background gas environment.
- Thickness can be controlled easily by regulating the number of shots, laser power, and gas pressure.

PLD disadvantages include a certain difficulty in achieving uniform films over large areas, the unavoidable modification of the target surface during the deposition process, occasional contamination of the film with splashing of micron‐sized particulates, and non‐congruent removal of the target material. These drawbacks have hindered the widespread adoption of PLD and impeded its industrial upscaling.

#### 2.6.2 Compositional Tuning of Organic–Inorganic Pb‐Based 3D Perovskites

In 2015, Bansode et al. introduced a room‐temperature dry process for the growth and stoichiometry control of MAPbI_3_ films by using nonstoichiometric single target ablation by excimer laser via off‐axis growth.^[^ ^211^ ^]^ The authors discussed the specific challenge related to PLD, namely the two substantially different properties of the volatile (easily vaporizable) organic and non‐volatile inorganic components of the hybrid materials. They studied the film growth and found that it occurs via the kinetics and thermodynamics of formation, dissolution, reactions, and evolution of small‐scale ion‐induced clusters, where the thermal stability of the cluster intermediates has a crucial impact on the film growth.^[^ ^211^ ^]^ For instance, the iodine in MAPbI_3_ evaporates quickly even at room temperature. As a consequence, a stoichiometric target (PbI_2_:MAI = 1:1) failed to produce the desired perovskite phase by the conventional on‐axis PLD approach, instead resulting in a dominant PbI_2_ phase. At the same time, a brown film was obtained at an off‐axis position (substrate perpendicular to the plume) that also lacked proper stoichiometry. An optimized ratio of PbI_2_: MAI  =  1:4 in the target facilitated the growth of near‐perfect stoichiometry in the off‐axis geometry, while an even higher portion of the organic component was required for the conventional on‐axis growth. The resulting films exhibited a pillar‐like structure separated by pinholes and voids as confirmed by both top and cross‐section images (Figure 19b). The addition of Cl^−^ to the target yields a similar surface morphology but a different bilayer‐type bottom structure near the substrate, while the use of F^−^ produced an intrinsic MAPbI_3_‐like structure. Ultimately, they were able to fabricate PSC devices using this adapted technique, with a structure of ITO/ZnO/MAPbI_3_:Cl/Spiro‐OMeTAD/Au, achieving a PCE of ≈7.7% (Figure 19c).

In a follow‐up study, the same authors further refined their PLD technique and reported that an off‐stoichiometric target (PbCl_2_: MAI = 1:12) and a momentum softening gas mixture of Ar and H_2_ (90%:10%) can produce a dense stoichiometric film with good optical quality.^[^ ^214^ ^]^ They tuned the bandgap from 1.6 to 2.3 eV, using either a mixed halide composite target or successive depositions of inter‐diffusing MAPbBr_3_ and MAPbI_3_ layers, claiming to be the first to achieve a fully dry process. A planar heterojunction solar cell with device architecture FTO/TiO_2_/MAPbI_3_/Spiro‐OMeTAD/Au based on the dry‐processed on‐axis PLD‐grown film exhibited a champion conversion efficiency of 10.9%.

In 2023, Soto‐Montero et al. reported a single‐step PLD of MA_1–x_FA_x_PbI_3_ thin films with tunable stoichiometry.^[^ ^212^ ^]^ By controlling the laser fluence and background Ar gas pressure, near‐stoichiometric transfer from target to film was achieved from a target containing 1:8 PbI_2_:(MA_1–x_FA_x_)I (x = 0.12–0.50). Films deposited with a stationary mode of the substrates showed an MA^+^: FA^+^ ratio close to that of the PLD target. An optimized MA^+^: FA^+^ ratio of 55:45 facilitated the room temperature formation and stabilization of cubic α‐phase MA_1–x_FA_x_PbI_3_ thin films with optimal optoelectronic properties (Figure 19d,e). A photoluminescence analysis confirmed the shift of the bandgap with varying MA^+^ and FA^+^ ratios. A proof‐of‐concept n‐i‐p solar cell with a device structure of glass/ITO/SnO_2_/PCBM/MA_0.55_FA_0.45_PbI_3_/Spiro‐OMeTAD/Au demonstrated PCE of 14% with no post‐treatment. This work foreshadows the feasibility of PLD for the in‐situ integration of new hybrid perovskites, wide‐bandgap perovskites for monolithic tandem devices with precise control over stoichiometry and hence optoelectrical properties. In subsequent work, the same authors applied chlorine passivation to further enhance the PLD‐processed MAFAPbI_3_ films, thereby achieving a champion PCE of 19.7 % (Figure 19f).^[^ ^213^ ^]^

As previously mentioned, solvent‐free fabrication methods often encounter challenges in precisely regulating perovskite nucleation and growth processes due to the inherent difficulty in introducing appropriate coordinating groups. Solomon et al. recently demonstrated that PLD enables room‐temperature epitaxial growth of MAPbI_3_ film on KCl substrates. The pulsed deposition characteristics of PLD facilitate the delivery of ablated species with substantial kinetic energy, thereby enhancing their surface mobility. This mechanism provides enhanced opportunities for substrate‐diffusing atoms to locate energetically favorable lattice sites on lattice‐matched substrates. In contrast to the continuous heating approaches employed in other dry fabrication techniques, the intermittent heating nature of PLD exhibits the potential for simultaneously controlling precursor kinetic energy and evaporation rates. This distinctive feature offers a viable strategy for balancing crystal nucleation and subsequent growth processes, thereby addressing a critical limitation in conventional dry synthesis methodologies.^[^ ^215^ ^]^

#### 2.6.3 PLD Growth of Inorganic and Lead‐Free Perovskites

PLD has also been successfully applied to the deposition of all inorganic perovskite compositions such as CsPbBr_3_, CsSnI_3_, and Pb‐free double perovskite Cs_2_AgBiBr_6_. In 2019, Wang et al. used PLD to successfully deposit CsPbBr_3_ thin films using targets made from CsPbBr_3_ single crystal powders.^[^ ^216^ ^]^ The substrates were heated to 320 °C during deposition, and post‐deposition annealing was performed for 2 h at 350 °C. The films were dense, compact, and thermally stable. Elemental mapping showed an atomic concentration of Cs, Pb, and Br close to 1:1:3. An optimal thickness of 200 nm for both m‐TiO_2_ and CsPbBr_3_ thin films was established, and a device with structure FTO/c‐TiO_2_2/m‐TiO_2_/CsPbBr_3_/Spiro‐OMeTAD/Ag resulted in a PCE of 6.3%. The authors attributed this performance to a dense and uniform CsPbBr_3_ layer that penetrated the m‐TiO_2_ layer with uniform distribution—contrary to the study by Gao et al., 2022 for sputtered films.^[^ ^148^ ^]^

Solvent‐free, single‐source, room temperature deposition of γ‐CsSnI_3_ through PLD was reported by Kiyek et al. in 2020.^[^ ^217^ ^]^ A solid target was fabricated by mixing CsI and SnI_2_ powders using both uniaxial and isostatic pressing. This target was then placed in a vacuum chamber and vaporized with a 248 nm KrF laser at room temperature. A 200 nm thin film of CsSnI_3_ was formed in 800 s without any additional reactive gas, resulting in perovskite layers with a bandgap of 1.32 eV.

The same group used PLD to deposit Pb‐free Cs_2_AgBiBr_6_. The target was ball‐milled from a stoichiometric CsBr, AgBr, and BiBr_3_ powder mixture.^[^ ^218^ ^]^ Controlled laser ablation of the double perovskite target with an Ar background pressure of 0.15 bar and at 200 °C substrate temperature formed highly crystalline Cs_2_AgBiBr_6_ films. The Ar pressure during deposition was identified as a critical factor for the film growth with proper stoichiometry, while elevated substrate temperatures improved film crystallinity.

#### 2.6.4 Modification of PLD Facilities

Dunlap‐Shohl et al. have introduced a new type of deposition method: resonant infrared matrix‐assisted pulsed laser evaporation (RIR‐MAPLE).^[^ ^219^ ^]^ They applied RIR‐MAPLE to fabricate MAPbI_3_ thin films and successfully integrated them into solar cells, achieving a PCE of 12%. The authors highlighted that the RIR‐MAPLE deposited MAPbI_3_ films exhibit comparable composition, morphology, and optical properties to those produced by conventional spin coating methods, suggesting that this new technique can produce device‐quality films.

#### 2.6.5 Conclusion on Pulsed Laser Deposition

PLD processing of perovskite photovoltaics has recently achieved notable advancements, establishing it among the most promising solvent‐free alternatives to TE. However, we believe that several aspects deserve further investigation. One key advantage of conventional PLD is the stoichiometric transfer of material from the target to the sample. This desirable property remains elusive for hybrid MHP, which are the most attractive compositions for optoelectronic and photovoltaic applications, necessitating targets with an over‐stoichiometric content of the organic component to date. Strategies should be established to reliably control film stoichiometry with great precision. Moreover, it is very likely that additives beyond the already reported halide salts will be necessary to further improve device performance.

Conventional PLD is valued for its capability to produce homogenous, high‐quality films with exact thickness control on the wafer scale. If the abovementioned challenges regarding stoichiometry control, additives, and potential dopants can be addressed, PLD might offer itself for the demanding fabrication of (opto) electronic devices that are conventionally processed on wafers, such as perovskite‐based LEDs, memory devices, and transistor circuits. **Table** **4** provides a summary of the herein‐discussed reports of PLD‐processed PSCs.

**Table 4:** Summary of PSCs based on pulsed laser deposition.

Perovskite	Small area photovoltaic parameters				Stability	Area [cm^2^]	Procedure	Architecture	Year	Refs.
	*V*_oc_ [V]	*J*_sc_ [mA cm^−2^]	FF [%]	PCE [%]						
MAPbI_3_	0.97	11.1	68	7.7	–	0.09	Target from PbCl_2_, MACl, and MAI (1:2:2) mixture, off‐axis sample position	ITO/ZnO/ Perovskite /Spiro‐OMeTAD/Au	2015	[211]
MAPbI_3_	0.98	20.1	46	10.9	–	0.09	Target from PbCl_2_ and MAI (1:12) mixture, Ar/H_2_2 (9:1) background gas	FTO/TiO_2_/ Perovskite /Spiro‐OMeTAD/Au	2017	[214]
CsPbBr_3_	1.37	6.4	72	6.3	–	–	Solution‐grown CsPbBr_3_‐single crystals, ground and pressed into a target.	FTO/TiO_2_/ Perovskite /Spiro‐OMeTAD/Ag	2019	[216]
MAFAPbI_3_	1.0	19.9	70	14	–	0.01	Target from PbI_2_2, MAI, and FAI mixtures (8× excess of organo‐halide content, varied MAI:FAI), treated in a rotary ball mill, Ar background gas.	ITO/SnO_2_/PCBM/ Perovskite /Spiro‐OMeTAD/Au	2023	[212]
MAFAPb(ICl)_3_	1.16	21.6	79.4	19.7	85% PCE after 1000 h at 85 °C	0.06	Target from PbI_2_, PbCl_2_, MAI, and FAI mixtures (PbI_2_/PbCl_2_:MAI/FAI = 1:8, MAI:FAI = 3:1), treated in a rotary ball mill, Ar background gas, 2D passivation.	ITO/2PACz/perovskite/C_60_/BCP/Ag	2024	[213]

### 2.7 Electron Beam Evaporation

To date, to the best of our knowledge, there is only one report on metal‐halide perovskites processed by means of electron beam (e‐beam) evaporation. In this study, Liu et al. obtained CsPbBr_3_ films with good uniformity using a single‐source e‐beam evaporation followed by a post‐annealing treatment, facilitating the fabrication of a solar cell with a PCE of 7.81%.^[^ ^220^ ^]^ However, the harsh conditions, namely high local temperatures and radiation involved in e‐beam evaporation, could make it challenging to deposit more delicate perovskite compositions, especially those containing organic cations or iodine, which tend to be less stable.

## 3 Solvent‐Free Printing

Printing is a transformative technology that has entirely changed the way we create patterns and designs on a variety of surfaces and is currently an essential instrument in contemporary manufacturing. Its inherent affordability is one of its main advantages and enables the realization of complex products without exorbitant costs. This adaptability extends to large areas and flexible substrates, facilitating manufacturing across various sizes and shapes. Consequently, printing emerges as a versatile solution that transcends the limitations of conventional methodologies for producing electronic devices, fostering vast opportunities for innovative and adaptable form factors. Printed electronics brings sleeker, lighter, more aesthetically pleasing, and environmentally friendly alternatives, hence closely aligning with the industry's roadmap for next‐generation electronics.^[^ ^156^ ^]^ This technology is i) driving a shift towards additive manufacturing in electronics, enabling the creation of significantly thinner form factors, ii) eliminating bulky discrete surface‐mounted device components with a large footprint, and iii) facilitating sustainable practices by utilizing recyclable and environmentally friendly materials.

Offering undeniable advantages in terms of cost and compatibility with a wide range of substrates, inkjet printing, aerosol‐jet printing, and other solution‐based printing methodologies have rapidly grown as viable technologies for manufacturing perovskite optoelectronic devices.^[^ ^221^ ^−^ ^223^ ^]^ Nevertheless, the high toxicity of commonly used solvents, such as DMF, chlorobenzene, and dimethyl sulfoxide, represents a significant drawback.^[^ ^224^ ^]^ The high‐volume processing of perovskite devices in large‐scale manufacturing processes will amplify the solvent toxicity problem, thus imposing additional challenges on the environment and the health of participating workers. In recent years, there has been an increasing interest in finding environmentally friendly or green solvents for perovskite manufacturing to minimize the impact of perovskite device manufacturing on the environment. Green solvents have replaced toxic alternatives in several instances, but it is crucial to consider how this may affect the functionality of perovskite devices. Unfortunately, so far, the resulting film quality, morphology, and device efficiency are subpar.^[^ ^225^, ^226^ ^]^

While solution‐based printing of perovskite films continues to be an invaluable and dynamic area of research, the quest for alternative, solvent‐free printing methods, capable of replicating the cost‐effectiveness, scalability, and versatility of solution coating, yet devoid of its inherent drawbacks, holds the key to unlocking the true potential of perovskites for next‐generation optoelectronic devices. This section examines the current state of the art in solvent‐free printing of perovskite devices, highlighting the advantages and challenges associated with each methodology.

### 3.1 Laser Printing

Laser printing is a high‐throughput, rapid, scalable, low‐cost additive printing method akin to solution coating, but with the advantage of being environmentally friendly. This solvent‐free coating method allows for simultaneous material deposition, patterning, and purification, eliminating the need for multiple steps and thus reducing costs and ecological footprint. Yet, despite its ubiquitous presence in office printing, it has surprisingly found limited application in the processing of electronic materials and devices.^[^ ^227^ ^−^ ^229^ ^]^

**Figure 20:**
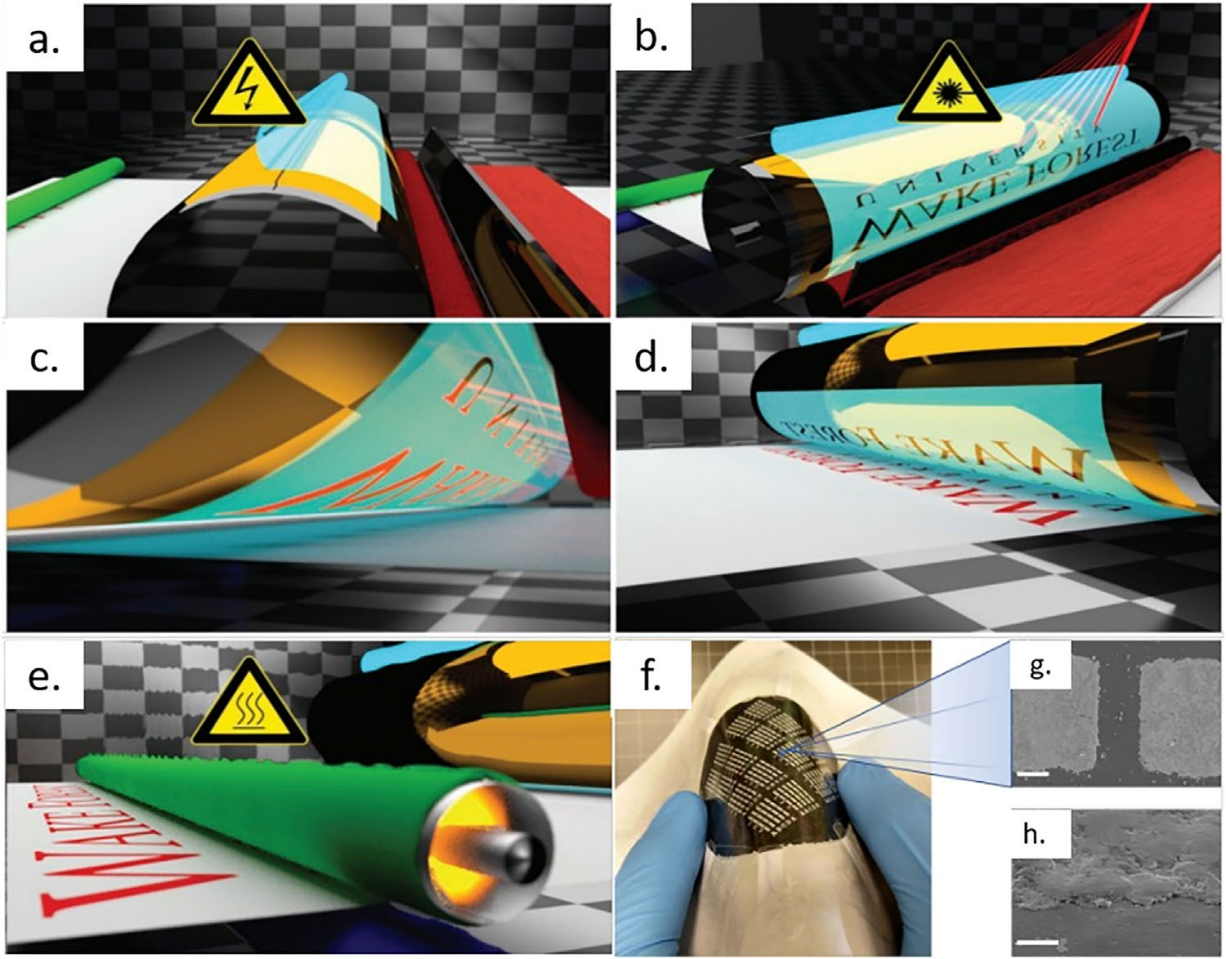
Laser printing of electronic devices. a) The OPC drum is statically charged, b) laser writing, c) toner developing, d) toner transfer, and e) the fusing process of laser printing. Reproduced with permission. Copyright 2022, Wiley‐VCH.^[^ ^227^ ^]^ Optical (f) and scanning electron microscopy images (g,h) of aerosol spray lithography contact patterns. This device was printed on a piece of standard printer paper. Reproduced under the Creative Commons license.^[^ ^228^ ^]^

Given the pervasive use of lasers in various perovskite processing and post‐processing techniques,^[^ ^230^ ^]^ it is important to distinguish the specific process of laser printing when discussing the manufacturing of electronic devices. This clarification is crucial to avoid confusion and ensure that “laser printing” is understood within the context of its unique application in device fabrication, distinct from other laser‐based processing methods. Therefore, we will start by describing the laser printing process for manufacturing perovskite layers. Laser printers create images by transferring the solid‐phase toner particles on the substrate through manipulating the electrostatic interactions between these particles and their designated locations, generating the desired pattern. The laser printing of the perovskite layer follows the steps shown in **Figure** **20**. A rotating cylindrical drum coated with an organic photoconductor (OPC) is initially uniformly charged (Figure 20a). Then, a laser beam selectively hits specific areas corresponding to the desired image (Figure 20b). The OPC layer preserves the charge in the dark but discharges when exposed to light. This selective discharge alters the electrostatic properties of the drum, allowing toner particles to adhere only to the areas that were exposed to the laser beam during the “development” step (Figure 20c). Subsequently, the OPC drum carrying the embedded image is rolled over the substrate, and the image is transferred (Figure 20d). Lastly, a fusing roller applies mild pressure and heat to embed the toner powder onto the substrate, creating a permanent image (Figure 20e).

The toner formulation plays a crucial role in the laser printing process. The toner consists of a fine powder composed of a mixture of pigment (perovskite), a polymer binder, and a charge control agent (CCA). The polymer, often called wax, acts as a rheological modifier, enhancing the adhesion to the substrate. The generated electrostatic forces control the transfer of the toner particles, and the CCAs regulate the polarity and the magnitude of the toner charge, ensuring optimal coulombic interactions. Solid‐state mixtures of these three components are prepared to obtain the perovskite toner. Their ratio is adjusted to provide efficient toner transfer and produce continuous, uniform laser‐printed films. To increase film uniformity, hexamethyldisilazane (HMDS) can be added to the toner to improve charge control and make the film more hydrophobic. The wax is added in a melt phase, while the other components are added in the solid phase. Mechanical grinding of the toner mixture by mortar and pestle, ball‐milling, or air jet milling is conducted to make the perovskite toner finer since the particle size correlates positively with the printing resolution. The fine toner powder is placed into the cartridge, which is then integrated into the printer.

Laser printing offers an additional advantage for creating perovskite devices by enabling precise patterning of the film. Patterning is essential for preventing crosstalk between individual devices within large‐area arrays. However, traditional techniques are not suitable for MHP semiconductor films because of their solubility in water and the ease of damaging their surface when exposed to photoresist or developer solvents,^[^ ^231^, ^232^ ^]^ which are key component in photolithography, leading to material degradation during the fabrication process. The simultaneous coating and definition of the desired pattern during laser printing represents a tremendous advantage over the traditional deposition methods, which typically yield blanket films. Another advantage of this solvent‐free method is that it eliminates the complications related to complex multi‐layer device architectures, where layer degradation often occurs because of interactions with the solvent of consecutive layers. Laser printing could potentially enable all‐printed devices on arbitrary substrates in conjunction with using aerosol spray lithography for defining contact patterns (Figure 20f–h).

**Figure 21:**
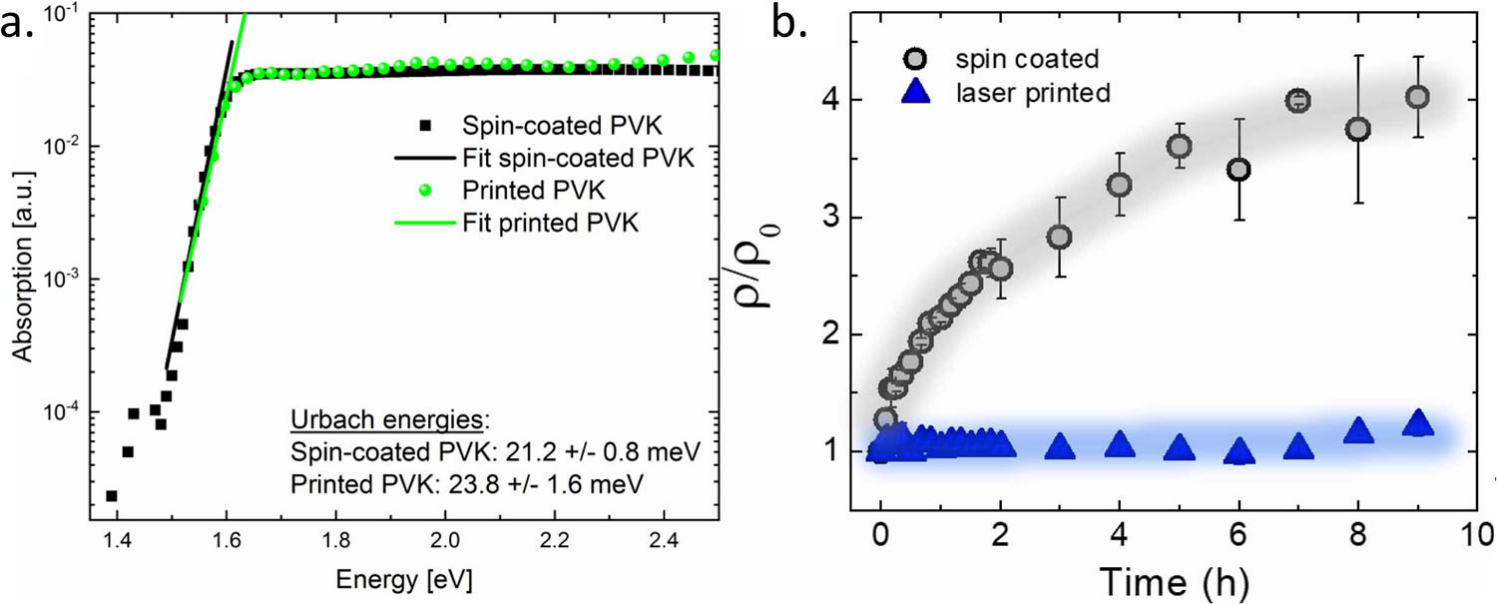
a) Absorption spectra of laser‐printed and spin‐coated perovskite films measured using photothermal deflection spectroscopy. b) Evolution of the normalized resistivity with respect to the initial value of the spin‐coated and laser‐printed perovskite films measured in dark and ambient as a function of time. Reproduced with permission. Copyright 2020, IOP Publishing Ltd.^[^ ^229^ ^]^

The first laser‐printed perovskite films were reported in 2020 by Tyznik et al., who deposited MAPbI_3_.^[^ ^229^ ^]^ Remarkably, these films demonstrated optoelectronic properties comparable to single crystals, despite their microstructure consisting of randomly oriented crystallites. Indeed, the sub‐band gap optical absorption edge spectrum showed similar values for the Urbach energy in the laser‐printed and spin‐coated perovskite films of 23.8 ± 0.8 and 21.2 ± 0.8 meV, respectively, indicating excellent electronic quality and a low degree of energetic disorder (**Figure** **21**a). Charge transport in these films approached the intrinsic limit, as determined from single‐crystal measurements, exhibiting negligible hysteresis in current‐voltage characteristics and remarkable environmental stability.

Figure 21b shows the evolution of film resistivities in laser‐printed perovskite films as a function of time; these films have been measured under ambient conditions, and the results obtained on spin‐coated films are also included as a reference. Over a 10 h period, the resistivity of the laser‐printed perovskite film exhibited minimal change, while that of the spin‐coated film increased significantly. The exceptional stability of the laser‐printed perovskite film is a direct consequence of the vertical phase separation that occurs during the printing process, leading to self‐encapsulation, with the non‐perovskite layers sandwiching the perovskite layer between them and acting as a protective barrier, shielding the perovskite layer from exposure to air and other environmental factors. These results reinforce the potential of laser printing as a solvent‐free, cost‐effective alternative to existing manufacturing techniques for the fabrication of flexible, large‐area electronic perovskite devices. However, while some areas exhibited properties comparable to single crystals in these first‐generation films, the properties varied across the film, and the dynamics of the phase separation of toner components substantially impacted the yield and homogeneity of the printed layers. Nonetheless, these results serve as a beacon, highlighting the potential performance levels achievable and hinting at the exciting applications that can be realized with a deeper understanding of the underlying film formation processes during laser printing. Transition of this technology from laboratory‐scale production to industrial applications depends on the ability to develop strategies for controlling film formation and enhancing film homogeneity to consistently achieve high performance across length scales relevant to applications.

### 3.2 Flash Evaporation Printing

Flash evaporation printing (FEP) is another solvent‐free method that is very fast, with deposition times in the order of seconds, making it well‐suited for large‐scale, high‐volume manufacturing. It involves rapidly heating a perovskite precursor to its evaporation temperature, followed by transporting the resulting vapor towards the substrate, where it condenses to form a thin film. The high temperatures, exceeding 1000 °C, employed in FEP facilitate the complete vaporization of all the organic and inorganic constituents from the target materials, leading to the formation of stoichiometric films, a feature often challenging to achieve through co‐evaporation.^[^ ^50^, ^111^, ^233^ ^−^ ^235^ ^]^

Flash‐evaporation printing has been first proposed by Wei et al. as a modified PVD method, meticulously crafted within a compact system engineered to attain rapid annealing.^[^ ^236^ ^]^ A modified FEP technique was developed by integrating PVD with a printing method, enabling efficient fabrication of high‐quality thin films. A key component of this FEP process is the flash‐evaporation transfer ribbon, constructed by coating target materials onto a freestanding carbon nanotube (CNT) sheet. This FEP method facilitates printing perovskite thin films through gas‐phase transport from the ribbon to the substrate.

The key to FEP's speed lies in the unique system geometry: the evaporator had a thickness of only ≈1.5 µm and a heat capacity per unit area of 0.1 J m^−2^ K^−1^, which is more than three orders of magnitude lower than that of the metal heaters used in typical TE.^[^ ^236^ ^]^ In the proof‐of‐concept demonstration, first, the target material (perovskite precursor) was printed on a CNT flash evaporator.

In this process, freestanding CNTs dry‐spun from the CNT arrays deposited on silicon wafers by CVD technique serve as the foundation for the flash evaporator. A cross‐stacked CNT sheet comprising 15 single layers was deposited on a metal frame, dipped in ethanol, and air‐dried at room temperature to generate a compact CNT layer. This layer is coated with a PbI_2_ solution via spin‐coating to fabricate the flash evaporation transfer ribbon. The resulting PbI_2_ film precursor was then heated at 100 °C for 10 min. This transfer ribbon fabrication process is scalable due to the availability of large‐area superaligned CNT arrays grown on 8‐inch wafers, which enables the production of ≈200 m‐long CNT thin films from each wafer.

Finally, the flash‐evaporation transfer ribbon was positioned 1 mm above a substrate, and a sample holder was used to place both the substrate and transfer ribbon within the compact FEP chamber. In order to make MAPbI_3_ perovskite film on a transporting layer for the fabrication of the solar cell device, the PbI_2_ film was deposited onto the substrate using the FEP. Subsequently, the perovskite layer was formed by dipping the PbI_2_ precursor in a 10 mg mL^−1^ MAI solution in isopropyl alcohol for 10 minutes, followed by drying at 100 °C for 30 min in a glove box.

**Figure 22:**
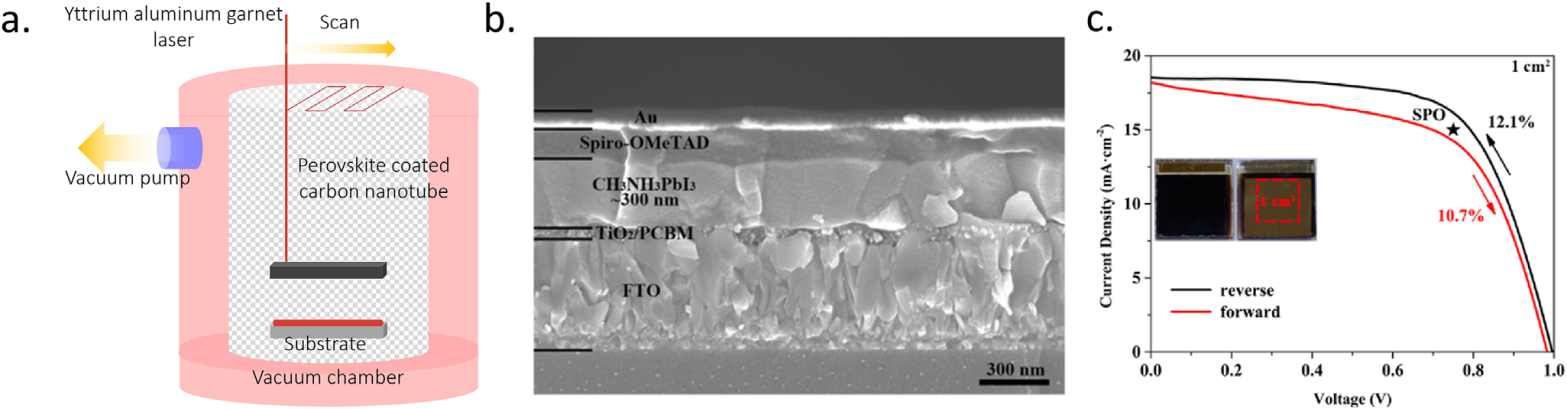
a) Illustration of the FEP equipment and process. b) Cross‐sectional SEM image of a PSC where the perovskite layer was coated with FEP. c) *J*−*V* curves of PSC with an active area of 1 cm^2^. Reproduced with permission. Copyright 2018, American Chemical Society.^[^ ^233^ ^]^

During the FEP process, a laser beam rapidly heats the source, causing the precursor to vaporize and condense on the nearby cold substrate, as shown in **Figure** **22**a. This process happens very quickly due to the source's rapid heating and cooling capabilities. The high responsivity of the CNT flash evaporator at high temperatures makes FEP a very successful technology. Essentially, FEP combines printing and vapor deposition to achieve a fast and efficient method for creating perovskite films.

Tai et al proposed a modified FEP for depositing perovskite films instead of the MAI dipping. In their work, PbI_2_:MAI at different ratios were prepared by dissolving the components in DMF. The perovskite precursor solutions were deposited onto the CNT sheets by spin coating and subsequent annealing at 100 °C for 5 minutes. Then, the samples were transferred into a vacuum chamber. Rapid evaporation of the precursor was achieved through laser scanning under a pressure of 3 × 10^−3^ Pa, and the films were then annealed.

Later, Tai et al deposited CsPbI_2_Br by FEP. A precursor solution containing CsI, PbI_2_, and PbBr_2_ in a 2:1:1 molar ratio was prepared in DMSO. A YAG laser (1.06 µm, 12 W) served as the heating source. The perovskite precursor solution was deposited onto the CNT sheets by spin coating and subsequently dried at 130 °C for 5 min. The precursor deposited CNT sheets, and the substrates were then transferred into a vacuum chamber. Rapid evaporation was induced by laser scanning along an S‐shaped route at a speed of 1 m s^−1^ under high vacuum conditions (3 × 10^−3^ Pa). The evaporated films were subsequently annealed under various conditions.

MAPbI_3_^[^ ^233^, ^236^ ^]^ and CsPbI_2_Br^[^ ^237^ ^]^ perovskite layers were printed through FEP, and the resulting films were incorporated into PSCs, as described below. To fabricate the MAPbI_3_ perovskite layer, a two‐step deposition process involving FEP and a dipping process was adopted. First, the PbI_2_ precursor solution was spin‐coated on the CNT sheets. Subsequently, the PbI_2_ film was transferred from the CNT substrate onto the PCBM/TiO_2_ stack using FEP, where an yttrium aluminum garnet (YAG) laser provided the heating source. Finally, the sample was immersed in an MAI solution to react and form MAPbI_3_.^[^ ^233^ ^]^ The MAI solution was based on isopropyl alcohol, which is a green solvent. The remaining device layers were spin‐coated, except for the gold electrode, which was thermally evaporated. Photovoltaic devices with the structure FTO/TiO_2_/PCBM/MAPbI_3_/Spiro‐OMeTAD/Au, where the perovskite layer was deposited using FEP, exhibited a PCE of 10.3%.^[^ ^236^ ^]^ This performance is nevertheless inferior to that obtained in solution‐deposited devices, which was attributed to the imperfections introduced by the conversion through immersion into the MAI solution. The surface of the partially FEP‐coated perovskite film was rough and had many defects, which ultimately restricted the PCE. The same group later improved the FEP technique and coated perovskite in one step.^[^ ^233^ ^]^ In their work, the CNT sheets were manufactured by CVD, and the perovskite precursor solution was deposited on top by spin coating as a target material. The CNT sheets were used as a material carrier, and perovskite flash evaporation was performed by using a YAG laser beam (wavelength of 1.06 µm) scanning under the pressure of 3 × 10^−3^ Pa. In Figure 22b, the cross‐sectional SEM image of the FTO/TiO_2_/PCBM/Perovskite/Spiro‐OMeTAD/Au solar cell structure, where the perovskite layer was FEP‐coated, is displayed; the resulting perovskite films coated through FEP have compact surfaces and are pinhole‐free. A PCE of 16.8% with a negligible hysteresis was achieved on an active area of 0.06 cm^2^. The authors also manufactured large‐area PSCs with a size of 1 cm × 1 cm, which yielded a PCE of 12.1% under reverse scan (Figure 22c).^[^ ^233^ ^]^ Tai et al. also reported single‐step FEP‐coated CsPbI_2_Br films, where they achieved a PCE of 12.2% with an active area of 0.06 cm^2^ and a PCE of 9.4% with an active area of 1 cm^2^.^[^ ^237^ ^]^ The successful fabrication of large‐area devices using FEP demonstrates its practicality and potential for scalability. However, the suboptimal PCE implies that further optimization is necessary. Refining the FEP method to produce high‐quality thin films, including, but not limited to organic–inorganic hybrid perovskites, is necessary for developing efficient and scalable applications. FEP enables the fabrication of patterned perovskite films using techniques such as shadow masking or point‐by‐point printing. The compact and scalable design of the FEP system makes it suitable for large‐area applications. This FEP technology holds significant promise for printing functional materials, with various applications in printed electronics, organic electronics, and future flexible electronics.

### 3.3 Transfer Printing

Transfer printing is a versatile deposition method that utilizes the controlled transfer of a thin film from one substrate to another. It finds applications in diverse fields, including the manufacturing of flexible electronics, the production of various functional layers, and the assembly of complex device structures.^[^ ^238^ ^−^ ^242^ ^]^ The steps included in the transfer printing process involve fabricating the material onto an interim substrate, lifting or detaching the material from this temporary substrate, and subsequently transferring it to the target substrate.

**Figure 23:**
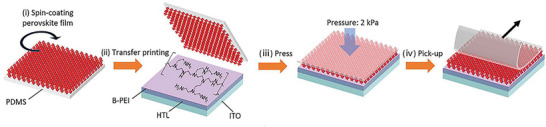
Illustration of the mass transfer‐printing process for perovskite films. Reproduced with permission. Copyright 2022, Wiley‐VCH.^[^ ^243^ ^]^

To transfer‐print perovskite films, the film was first spin‐coated on a poly(dimethylsiloxane) (PDMS) substrate as the transfer area, as shown in **Figure** **23**. The use of ultrathin branched polyethylenimine (B‐PEI) interfacial chemical bonding layers enabled the fabrication of transfer‐printed perovskite films with morphology, composition, and optoelectronic properties comparable to those of the spin‐coated counterparts.^[^ ^243^ ^]^ The receiver (hole transporting layer in Figure 23) was coated with an ultrathin amine functional polymer, B‐PEI, to create chemical bonds between the NH_2_ group and Pb‐I octahedral, improving the contact of HTL with the perovskite layer. Perovskite LEDs fabricated using the transfer‐printed films demonstrated high external quantum efficiencies of 10.5% and 6.7% for red (680 nm) and sky‐blue (493 nm) emissions, respectively.^[^ ^243^ ^]^ Furthermore, this robust transfer printing method enabled the fabrication of high‐resolution perovskite micropatterns with pixel densities up to 1270 pixels per inch. By employing multiple printing processes, horizontally aligned red and sky‐blue perovskite microstrips were successfully produced, opening up exciting possibilities for real‐world applications in full‐color displays, white LEDs, lasers, and other optoelectronic devices.

Successful transfer printing of the perovskite films requires a higher separation energy at the perovskite/substrate interface *G* ^Perovskite/Substrate^, than at the perovskite/PDMS interface *G* ^Perovskite/PDMS^(*v*) *= G*_0_^Perovskite/PDMS^[1+ Φ(*v*)], where Φ is directly proportional to the velocity.^[^ ^244^ ^]^ The significantly higher G_0_^Perovskite/Substrate^ adhesion strength compared to G_0_^Perovskite/PDMS^ stems from the exceptionally low surface energy of PDMS (19.8 mJ m^−2^) compared to most organic films and glass (>200 mJ m^−2^).^[^ ^245^ ^]^ Based on these considerations, a pressure of 2 kPA was required to achieve a peeling speed of 1 cm s^−1^ for the transfer printing process.

For PeLED fabrication by the transfer printing process, first, red and sky‐blue perovskite solutions were prepared. A 0.2 m solution of Cs_0.7_MA_0.3_Pb(I_0.8_Br_0.2_)_3_ was prepared by dissolving stoichiometric amounts of PbI_2_, MAI, CsI, PbBr_2_, FPMAI, and PEABr in DMF. An additional 60 mol% excess of FMAI and PEABr (FMAI:PEABr molar ratio is 0.8:0.2) was added to the perovskite solution. A 0.3 m solution of CsPb(Br_0.84_Cl_0.16_)_3_ was prepared by dissolving stoichiometric amounts of CsBr and PbBr_2_ in DMSO. An additional 20 mol% excess of MABr and 60 mol% excess of PEACl were added to the perovskite solution. Cs_0.7_MA_0.3_Pb(I_0.8_Br_0.2_)_3_ perovskite solution with a toluene antisolvent and CsPb(Br_0.84_Cl_0.16_)_3_ perovskite solution with a 1:1 (v/v) toluene:chloroform antisolvent were spin‐coated on the PDMS substrates at 5000 rpm. Later, the perovskite‐coated PDMS was brought into contact with the target substrate at 2 kPa pressure, and the PDMS was picked up at a speed of 1 cm s^−1^. For removing residual solvent from the film, the transferred Cs_0.7_MA_0.3_Pb(I_0.8_Br_0.2_)_3_ and CsPb(Br_0.84_Cl_0.16_)_3_ films were annealed at 70 and 50 °C for 10 min, respectively. To fabricate white PeLEDs, the mixed CsPb(Br_0.84_Cl_0.16_)_3_ perovskite layer was transfer‐printed first. After the CsPb(Br_0.84_Cl_0.16_)_3_ perovskite was coated onto the PDMS by spin coating, a poly(N‐vinylcarbazole) (PVK) layer (3 mg mL^−1^) was deposited on top of the sample. To transfer the perovskite nanostripes onto the poly[N,N′‐bis(4‐butylphenyl)‐N,N′‐bis(phenyl)‐benzidine] (poly‐TPD) substrate, the PDMS was brought into contact with an intaglio silicon template under a pressure of 20 kPa and peeled off at a speed of ≈5 mm s^−1^, and the sample was then annealed at 50 °C for 10 min. For the second layer, Cs_0.7_MA_0.3_Pb(I_0.8_Br_0.2_)_3_ solution was spin‐coated onto the PDMS substrate pre‐coated with a PMMA layer. The perovskite stripes were fabricated using the same transfer printing method. A custom‐built alignment system with 10 µm resolution 3D movable platform, and an optical vision system was employed to align the second layer with the first one. The final film was annealed at 50 °C for 10 min.

Transfer printing allows for roll‐to‐roll processing, which enables high‐throughput and potentially low‐cost fabrication of perovskite devices. This is crucial for commercialization and large‐scale applications. Transfer printed perovskite layers can be deposited on flexible and non‐planar substrates, and could therefore be used for the development of flexible electronics, wearable devices, and medical applications. Compared to other techniques, transfer printing can offer better control over film thickness, morphology, and grain size, resulting in potentially higher device efficiency and reproducibility, while also minimizing material waste by using pre‐formed perovskite layers, leading to more sustainable manufacturing. The challenges in implementing this technique and adopting it in an industrial setting arise from the fact that it typically involves multiple processing steps and requires simultaneous and precise control over multiple parameters like temperature, pressure, and surface properties. This complexity can increase costs and introduce new failure points. Additionally, the sensitivity to moisture and heat typical to perovskite layers can be exacerbated during the transfer process and could affect device stability and lifetime. Achieving high transfer efficiency and ensuring uniform layer thickness across large areas can be challenging, potentially impacting device performance and reproducibility.

Transfer printing halide perovskite layers offers exciting opportunities for scalable, high‐performance device fabrication. However, addressing the technical challenges and improving cost‐effectiveness are critical for its wider commercialization and impact on various industries.

### 3.4 Dry Powder Aerosol Deposition

Dry Powder Aerosol Deposition (DPAD), also known as the Aerosol Deposition Method (ADM), is commonly employed for the manufacturing of dense ceramic films at room temperature directly from ceramic powder, which eliminates the requirement for chemical reactions or phase transformations.^[^ ^246^, ^247^ ^]^ This method has been demonstrated for the deposition of various functional materials, such as TiO_2_ for dye‐sensitized solar cells,^[^ ^248^ ^]^ oxidic perovskites,^[^ ^249^ ^]^ metal oxides,^[^ ^250^, ^251^ ^]^ halide perovskites for solar cells,^[^ ^252^ ^]^ and inorganic perovskites for humidity sensors.^[^ ^253^ ^]^

In contrast to the aerosol‐assisted CVD already in use for halide perovskites,^[^ ^254^, ^255^ ^]^ DPAD is a solvent‐free method. The dry nature of the ADM provides several advantages, including the low environmental impact and the ability to coat uniform films.

**Figure 24:**
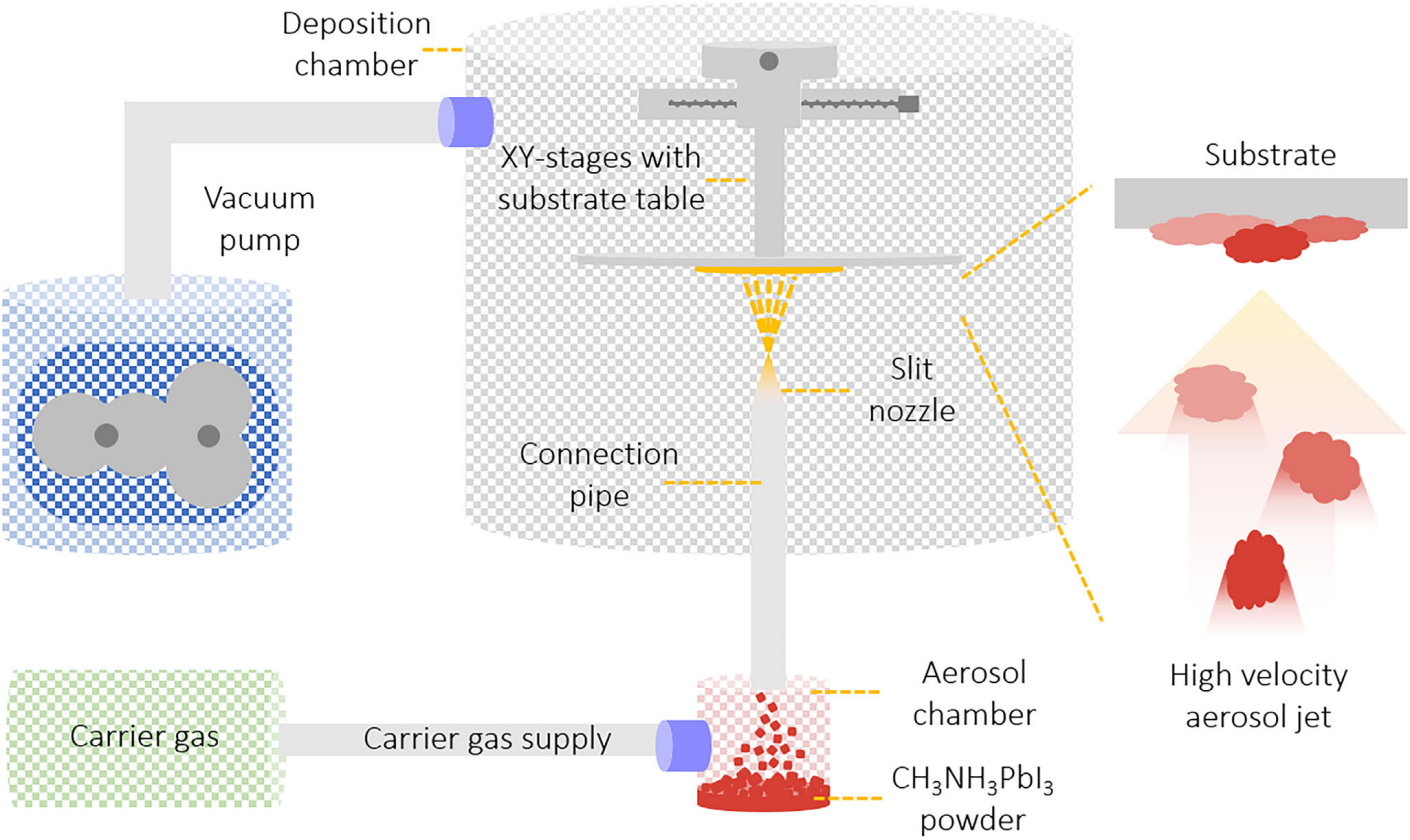
Schematic illustration of an aerosol deposition process. Reproduced with permission. Copyright 2016, Multidisciplinary Digital Publishing Institute.^[^ ^257^ ^]^

In the PAD process (**Figure** **24**), a carrier gas (e.g., N_2_, He, or O_2_) is directed through a powder within an aerosol chamber, which creates a dry powder aerosol.^[^ ^246^, ^247^, ^256^ ^]^ The substrate is placed in the deposition chamber, which is then evacuated to ≈1 to 10 mbar using a vacuum pump. This pressure gradient introduces a flow of the dry powder aerosol, which migrates into the chamber through a pipe. Afterward, the aerosol is accelerated to a high velocity ranging from 100 to 600 m s^−1^, generating a high‐velocity aerosol jet, facilitated by a nozzle. This accelerated jet is then directed toward the substrate, where it undergoes film formation upon impact and consolidation of the particles. Several processing parameters, including particle size, material hardness, and the velocity of the particle jet, play important roles in generating a high‐quality, uniform film via Aerosol Deposition. The intricate interplay of these factors affects the efficacy of the film formation process. In this method, the properties of the perovskite, which result from the exact synthesis procedure, and the formation of the film are closely related. The changes in the processing of dry powder aerosol‐deposited perovskite films can lead to differences in film quality and, ultimately, variations in the optoelectronic properties. Therefore, it is not easy to optimize the characteristics of the perovskite films independently from the synthesis of the perovskite.

Panzer et al. first deposited MAPbI_3_ perovskite layers by ADM in 2016.^[^ ^257^ ^]^ and tuned layer thickness with high precision. They first synthesized MAI powders by mixing PbI_2_ and MAI in a 1:1 molar ratio. This mixture was then dispersed in 2 mL of DMF and stirred for 30 minutes. The mixture was degassed under a nitrogen atmosphere for 30 min to remove dissolved gases. The mixture was dried under a nitrogen atmosphere at 100 °C, and the dried powder was then ground to finer particle sizes using a mortar. The AD system has three primary components: a deposition chamber, a vacuum pump, and an aerosol generation unit. In the aerosol generation unit, a controlled flow of carrier gas (N_2_) is directed to the perovskite powder, resulting in the formation of aerosolized particles. A pressure difference between the aerosol generation unit and the deposition chamber was generated by a vacuum pump. This pressure difference leads to the aerosol flow into the deposition chamber through a connecting pipe. To generate a high‐velocity jet, at the exit of this pipe, a slit nozzle is placed to further accelerate the aerosol flow. This high‐velocity jet is then precisely directed towards a movable substrate. The perovskite particles within the jet consolidate on the substrate and generate a thin film.

In their work, the perovskite layer showed a strong adhesion to the TiO_2_ substrate, with the thicknesses ranging from a few micrometers to less than 1 µm. No phase impurities could be detected in XRD patterns of films, and the obtained PL spectra were characteristic of pure MAPbI_3_. These results confirmed the non‐destructive nature of PAD as a processing method for halide perovskites. However, the high surface roughness of the layers posed a significant challenge for the successful manufacturing of efficient optoelectronic devices based on the dry aerosol‐deposited perovskite layer. In 2019, Kim et al. utilized DPAD to deposit CsPbBr_3_/Al_2_O_3_ composite powders, resulting in films with a thickness in the low micrometer range.^[^ ^258^ ^]^ These films have been reported to exhibit exceptional long‐term stability, enduring 20 days at 150 °C or over 5 months under ambient conditions. Furthermore, they have also proven effective as green emitter layers in white light backlight units. The versatility of DPAD was further demonstrated by its ability to process patterned shapes. In 2020, Cho et al. combined CsPbBr_3_ and CsPb_2_Br_5_ perovskites with various ceramics (Al_2_O_3_, TiO_2_, and BaTiO_3_), and these unique nanocomposites were then deposited by aerosol deposition.^[^ ^253^ ^]^ To prepare the ceramic‐perovskite nanocomposite films by using the AD process, the nanocomposite powders were kept in a dry oven at ≈100 °C for at least 24 h prior to use to eliminate any residual moisture or aggregates. After the drying process, the powder was sieved using a fine mesh to ensure a homogeneous‐fine dispersion, and the nanocomposite perovskite powders were placed within the aerosol chamber. A highly pure nitrogen carrier gas is introduced into the chamber to propel the powder particles. To diminish the deceleration of the particles exiting the nozzle, both the aerosol and the deposition chambers are pre‐evacuated with a vacuum pump to a pressure of ≈3.2 Torr. During the deposition of the film, the nanocomposite perovskite particles, including blown aerosol, were carried by the nitrogen gas through a Teflon tube and then through a stainless‐steel nozzle. The accelerated particles then impacted the substrate, which was placed at a distance of 5 mm from the nozzle outlet. The strong cohesive forces between the particles (both particle‐to‐particle and particle‐to‐substrate) yielded the formation of the nanocomposite perovskite film.

A humidity sensor utilizing the CsPb_2_Br_5_/BaTiO_3_ nanocomposite, prepared through aerosol deposition, showed higher sensitivity when compared to CsPbBr_3_/Al_2_O_3_ and CsPbBr_3_/TiO_2_ sensors. The nanocomposite exhibited high humidity sensitivity (21426 pF RH%^−1^), superior linearity (0.991), rapid response/recovery time (5 s), low hysteresis (1.7%), and high stability. The first solar cell including a completely dry‐processed powder‐based MAPbI_3_ absorber layer was reported in 2023 by Biberger et al.^[^ ^252^ ^]^

MAPbI_3_ perovskite films were coated using a custom‐made PAD apparatus. The mechanochemically synthesized MAPbI_3_ powders were kept at 120 °C for a minimum 1 h to dry. Then, one gram of this dried powder was loaded into the aerosol generation unit of the PAD system. Helium gas was used as the processing gas, flowing at a rate of 0.25 L min^−1^ to effectively generate the aerosol. To increase the acceleration of the generated aerosol, an ejector was placed into the system, delivering a flow rate of 20 L min^−1^, and then the aerosol was directed through a converging slit nozzle with dimensions of 10 mm × 0.5 mm. The distance between the nozzle and the substrate was kept at 3 mm. During film deposition, the substrate was moved past the nozzle 30 times at a velocity of 1 mm s^−1^, and the generated pressure within the aerosol generation unit and the deposition chamber was at 122 and 6 mbar, respectively.

In order to improve the perovskite film quality, a hot pressing posttreatment step was performed by using glass blocks with a thickness of 8 mm and roughness of 2 nm as press die material. This material is covered with a silanization coating (trichloro(octadecyl)silane in toluene from the liquid phase) to hinder sticking to the perovskite film with the glass blocks. The films were pressed under 25 MPa at 120 °C for 5 min with anti‐sticking coated glass blocks.

The MAPbI_3_ active layer with a thickness of 1 µm was coated using the PAD. They also applied a post‐treatment with (hot‐) pressing of the films. This led to larger grain size, enhanced crystallinity, reduced surface roughness and energetic disorder, and a longer charge‐carrier lifetime. They also showed that PAD yields an increase in the disorder of the SnO_2_ ETL of the solar cell in absorption measurements, which was related to the defects at higher intensity within SnO_2_ because of high mechanical impact. The champion device achieved a PCE exceeding 6%, with a V_oc_ of 0.95 V and a fill factor (FF) of 56%. Nevertheless, these devices exhibited a low short circuit current (*J*_sc_) of 7.6 mA cm^−2^, which restricted the performance of the PAD‐processed solar cells.

### 3.5 In Situ Conversion

**Figure 25:**
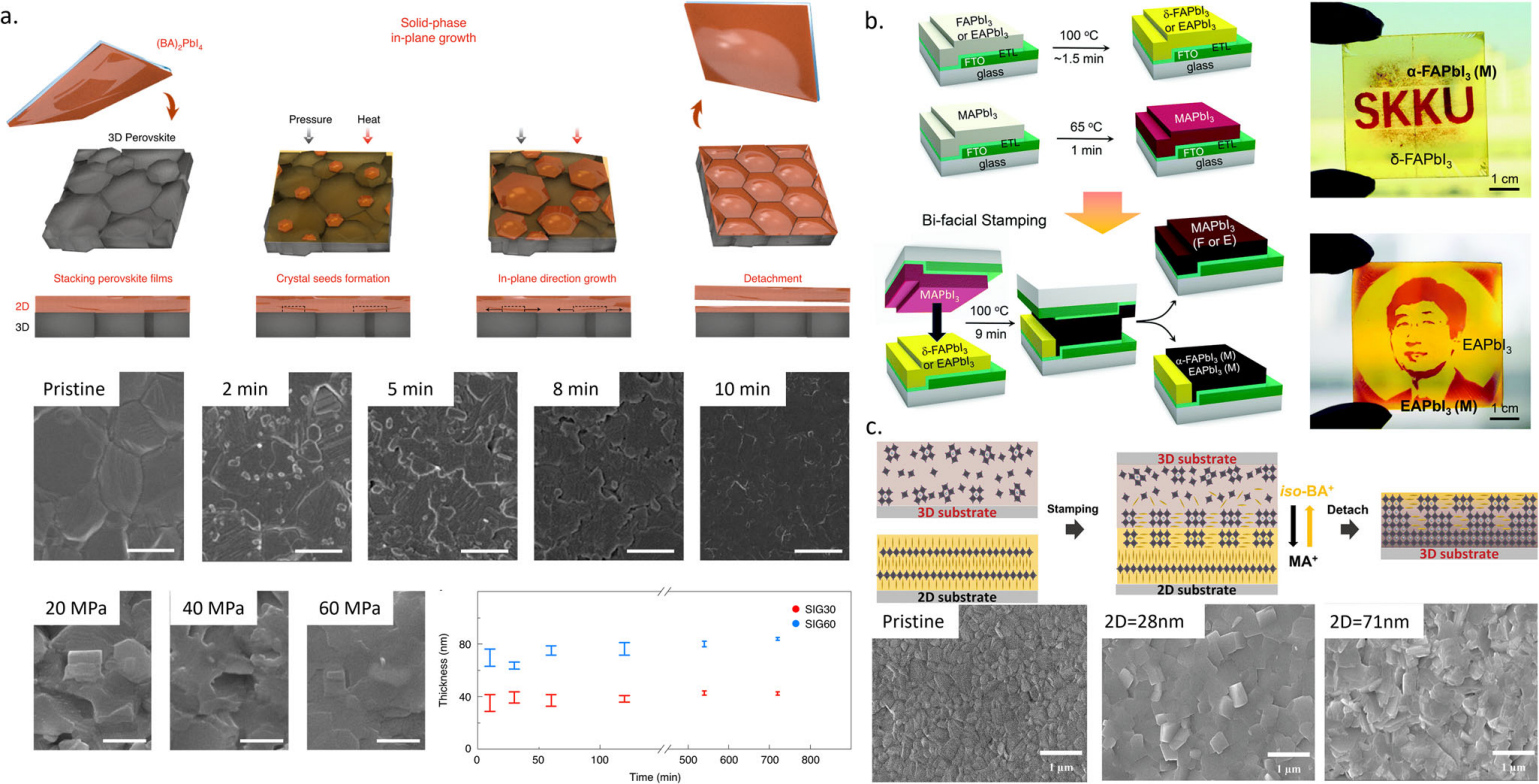
a) Formation of 2D (BA)_2_PbI_4_/ 3D (FAPbI_3_)_0.95_(MAPbBr_3_)_0.05_ heterostructures by in‐situ conversion. Reproduced with permission. Copyright 2021, Springer Nature.^[^ ^259^ ^]^ b) A bifacial stamping method of MAPbI_3_ onto FAPbI_3_ or EAPbI_3_. Reproduced with permission. Copyright 2019, Royal Society of Chemistry.^[^ ^260^ ^]^ c) Formation of reverse graded quasi‐2D layers. Reproduced with permission. Copyright 2022, Wiley‐VCH.^[^ ^261^ ^]^

Solid in‐situ conversion is a method for creating single‐layer or multi‐layer perovskite films by applying pressure or heat to trigger in‐situ reactions and ion exchange between solid films or between solid films and powders, thereby obtaining a film or structure with the required composition and thickness. Zhu et al. demonstrated this technique by placing a layer of MAI powder onto a film comprising a mixture of PbCl_2_ and CsCl.^[^ ^113^ ^]^ Upon heating to 150 °C for 20 minutes, PbCl_2_ and CsCl were converted into high‐quality, pin‐hole‐free MA_0.77_Cs_0.23_PbI_3_ films with uniform crystal grains. This method offers several advantages. It avoids the difficulties of using MAI in traditional TE methods, where its volatile nature makes it challenging to control. It also provides excellent reproducibility, meaning the process consistently produces high‐quality films. Jang et al. adopted the solid in‐situ conversion method to fabricate 2D‐3D perovskite hetero‐structures, growing a 2D perovskite layer on a 3D perovskite layer by applying pressure and heat.^[^ ^259^ ^]^ The in‐situ conversion prevented the formation of unintended phases and allowed the adjustment of the thickness of the 2D layer (**Figure** **25**a). Notably, the possibility of tuning the thickness of the 2D perovskite layer enabled fine control of the built‐in potential at the junction. Moreover, the stable 2D layer provided full coverage of the 3D surface and improved the stability of the entire layer stack. While similar structures have been fabricated through solution processes, these techniques, thus far, have led to the unintended formation of a quasi‐2D phase with adverse effects on uniformity and stability.

The so‐called “bifacial stamping” method, which resembles solid in‐situ conversion, was proposed by Zhang et al.^[^ ^260^ ^]^ Two films are brought into contact and heated to induce a reaction. In this case, they sandwiched FAPbI_3_ and MAPbI_3_ and annealed them at 100 °C for 9 min without applying pressure. This treatment resulted in a fast and complete δ to α phase transformation of FAPbI_3_ and effectively reduced recombination losses at the surface of MAPbI_3_, ultimately improving the material's performance in solar cells. By replacing FAPbI_3_ with CH_3_CH_2_NH_3_PbI_3_ (EAPbI_3_), the initially photoinactive (yellow) EAPbI_3_ was transformed into photoactive EAPbI_3_ after stamping with MAPbI_3_ (Figure 25b). The same strategy was applied by Lee et al. to prepare highly oriented and reverse‐graded 2D perovskite films by placing 3D perovskite films face down on 2D perovskite films.^[^ ^261^ ^]^ The spatial orientation is caused by the low supersaturation during crystallization, allowing heterogeneous crystal growth from the contact interface between the 2D and 3D perovskite films and inducing a strong vertical orientation of the 2D perovskite (Figure 25c). Ma et al. showed that bifacial stamping is compatible with roll‐to‐roll processes.^[^ ^262^ ^]^ However, although this solid‐solid reaction offers various advantages regarding the control of composition and film quality, its reaction principle is inseparable from the DMSO‐containing adduct, a media that triggers the ion exchange reaction. Therefore, other additives must be explored to achieve a fully solvent‐free process.

## 4 Melt‐Assisted Growth

**Figure 26:**
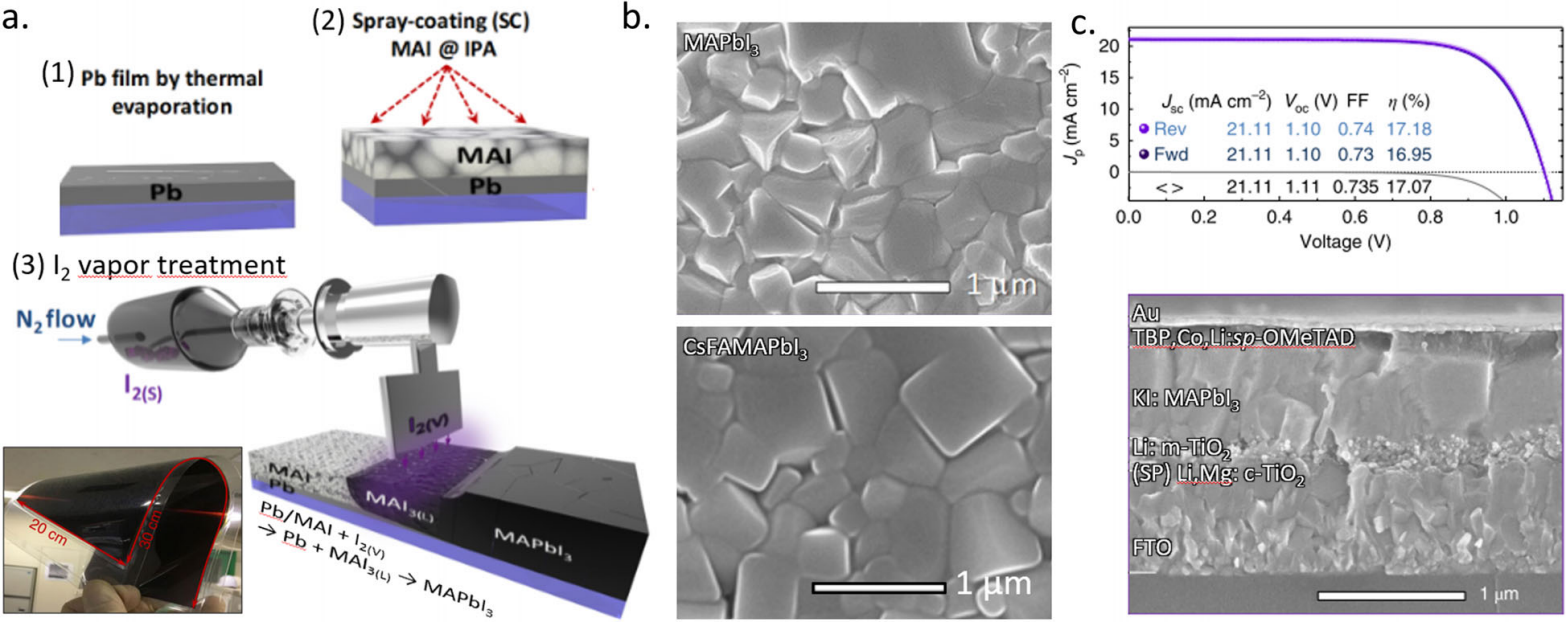
Melt‐assisted growth **a**. Illustration of layer growth via the evaporation‐solution‐vapor route. Inset image displays an encapsulated flexible 600 cm^2^ film on a flexible PET/ITO substrate. b) Top‐view SEM images of melt‐processed MAPbI_3_ and CsFAMAPbI_3_ films. c) *J*–*V* characteristics of melt‐processed PSC and cross‐section SEM image. Reproduced with permission. Copyright 2018, Springer Nature.^[^ ^267^ ^]^

While the other herein‐reviewed deposition methods rely on solid, gaseous, or melted precursors at high temperatures, another unique method uses so‐called reactive polyhalide melts (RPMs) that are liquid at room temperature.^[^ ^263^ ^]^ As first reported by Petrov and coauthors in 2017, RPMs react from mixtures of halide salts and elemental iodine, forming compounds such as MAI_x_ (2 ≤ x ≤ 5.5), FAI_x_ (2 ≤ x ≤ 5.5), and FA_1–x_Cs_x_I_4_ (0 ≤ x ≤ 0.3) spontaneously and at a temperature below 50 °C.^[^ ^264^ ^]^ RPMs are highly reactive and convert thin layers of metallic lead into APbX_3_‐type perovskite. When adapted for thin‐film device fabrication, the application of liquid RPM onto lead films, e.g. via spin coating, makes it difficult to control stoichiometry, while the rapid reaction complicates control over film morphology.^[^ ^263^, ^265^, ^266^ ^]^ In an alternative approach, a thin (multi‐)layer of a halide salt was deposited onto metallic lead with stoichiometric amounts, either by evaporation or from solution.^[^ ^266^ ^−^ ^268^ ^]^ Then, the layer stack was exposed to iodine vapor (**Figure** **26**a). The elemental iodine reacted with the halide salt, forming the RPM as observed by Raman spectroscopy, which then successively reacted with the metallic lead to form the perovskite (Figure 26b). This method, although involving multiple distinct processing steps that increase manufacturing complexity, generated highly crystalline and uniform mixed‐cation and mixed‐halide perovskite films, leading to first reports of RMP‐processed PSC in the n‐i‐p architecture (FTO/TiO_2_/MAPbI_3_/spiro/Au) with a peak performance around 17 % (Figure 26c).^[^ ^267^ ^]^ The absence of solvents enabled high film purity and eradicated drying and shrinkage related defects. In contrary, the volumetric expansion of from lead to lead‐halide perovskite seems to eliminate pin‐holes.

The omission of not only solvents, but also water‐soluble lead salts in favor of metallic lead improves environmental and workplace safety. Regarding technological opportunities, RPMs offer a pathway to investigate novel compositions that are not compatible with solution processing, as was shown by a report on gold‐based perovskite.^[^ ^269^ ^]^ Moreover, some material systems that are not viable under solution processing, such as perovskite on CuI, can be processed using this method.

In‐situ RPM processing might become a viable alternative or complement to conventional evaporation. Although having similar advantages and limitations, this approach allows to separate the relatively facile deposition of material onto a substrate from the complex process of perovskite formation, potentially promoting scalability, reproducibility and homogeneity. Whether this proves to be advantageous is subject to evaluation. To date, the efficiency of RPM‐processed mixed‐cation/mixed‐halide PSCs is substantially lower than that of comparable evaporation‐processed material systems. This discrepancy might be rooted in the community's limited optimization efforts, as only a few groups have adopted this method. Inherent challenges will be the precise control of stoichiometry, especially the halide‐to‐lead ratio, and additive engineering. While these two aspects lead to crucial advancements in other, more mature fabrication techniques, it is not immediately obvious how these can be considered in RPM‐assisted growth. More rigorous investigations are needed to evaluate the potential of RPM in PSC fabrication properly.

## 5 Supplement: Solvent‐Free Fabrication of Perovskite Source Materials for Thin Film Deposition

Many of the thin‐film deposition techniques discussed herein require source materials such as perovskite powders, crystals, or targets. This chapter briefly introduces two solvent‐free methods, mechanosynthesis and vertical Bridgman growth, for preparing such perovskite bulk material. Additional resources are referenced within the sections for a deeper understanding of these techniques.

### 5.1 Mechano‐Synthesis

#### 5.1.1 Mechanism and Advantages of Mechano‐Synthesis of Perovskites

**Figure 27:**
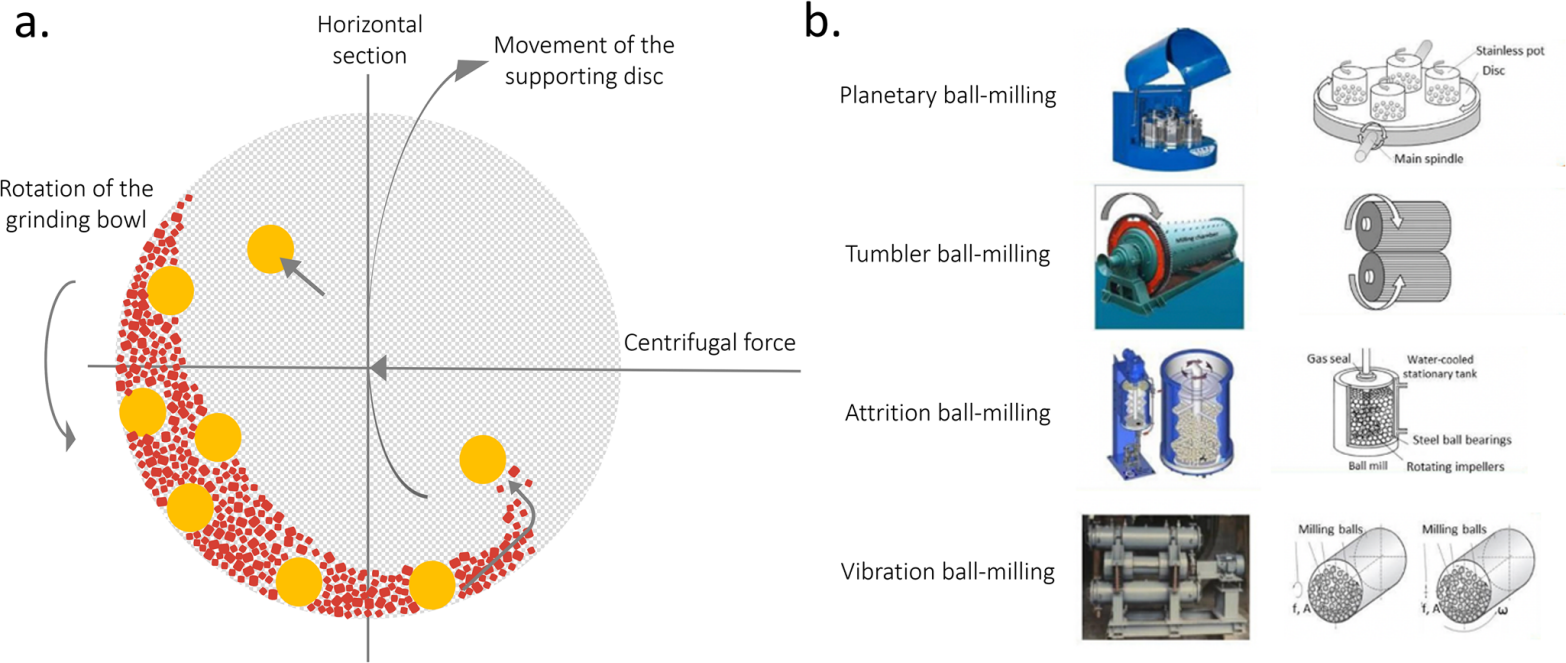
a) Schematic illustration of the working principle of the ball milling process. b) Different types of ball milling and their working principles. Reproduced with permission. Copyright 2020, Elsevier Limited.^[^ ^276^ ^]^

While the synthesis of perovskite powders is widely realized through solvent‐based wet chemistry, mechano‐chemistry offers an alternative, solvent‐free approach.^[^ ^270^ ^]^ This section provides a brief overview of the dry mechanical processing of the perovskite precursors as a means to prepare source material for solvent‐free thin‐film deposition. For an in‐depth discussion of the field, the available literature should be consulted.^[^ ^271^ ^−^ ^273^ ^]^ Mechano‐synthesis is a solid‐state synthetic technique in which mechanical energy absorption and conversion through impact, compression, or shear on reactants leads to chemical reactions. In short, when the precursor mixture is crushed between two solid objects, mechanical energy is transferred to the precursors, leading to deformation, fractures, and dislocations. This process drives the reaction to form the product as a fresh surface is repeatedly exposed.^[^ ^273^, ^274^ ^]^ Due to the low formation energy of perovskites and the soft crystal structure of the organic components, perovskite crystals can be easily synthesized by grinding the components, for instance, organic ammonium halide and inorganic metal halide precursors, in a mortar‐pestle or a ball mill with desired stoichiometry. An automatic shaker mill or planetary ball mill can provide better control over the experimental conditions with jars and balls made of hardened materials such as stainless steel, zirconia, agate, etc. The resulting powder size is controlled through various parameters, including rotation speed, rotation time, and ball‐to‐powder ratio.^[^ ^275^ ^]^ A schematic of various ball milling types and ball milling methods is shown in **Figure** **27**.

The fabrication of perovskite powders via dry ball milling has several advantages:

- Mechano‐synthesis can enable the synthesis of new material compositions by evading the solubility limitations of the used precursors, which otherwise is not possible by means of solution processing.^[^ ^273^ ^]^
- Mechanochemically synthesized cubic α‐phase CsPbI_3_ and FAPbI_3_ powders exhibit excellent long‐term storage stability.^[^ ^277^, ^278^ ^]^ MAPbI_3_ powder exhibited a high stability under humidity. This was attributed to a lower inclination angle of the PbI_6_ octahedrons, likely caused by a small MA deficiency, leading to a higher Goldschmidt's tolerance factor.^[^ ^279^ ^]^
- Large‐scale ball milling is already suitable to produce batch sizes at an industrially relevant scale,^[^ ^280^, ^281^ ^]^ thereby promoting large‐scale production as reported previously.^[^ ^93^, ^277^ ^]^
- Mechano‐synthesis offers good control over the stoichiometry of the final product and does not require toxic solvents, reducing its environmental impact and potentially promoting worker safety.

While mechano‐synthesis offers a convenient way to produce perovskite materials, this method has a few disadvantages. One issue is the lack of real‐time monitoring of the internal temperature, which can lead to inconsistent results at extreme conditions, such as very high rotation speeds, long run times, or extreme ball‐to‐powder ratios. Another concern is contamination, which can occur due to the wearing and tearing of the bowl and grinding balls, especially with prolonged, improper handling, or when processing materials with low hardness, e.g., steel.

#### 5.1.2 Mechano‐Synthesis of Perovskites

In 2013, Stoumpos et al. pioneered the use of mechano‐synthesis to create both lead‐based and tin‐based perovskites.^[^ ^93^ ^]^ They prepared Pb‐based and Sn‐based perovskites by hand grinding mixtures of metal iodide and organic iodide salt in an agate mortar until they turned black, indicating perovskite formation. Most works on ball‐milled organic‐inorganic lead‐halide perovskites used the obtained powders in solution‐based processes to fabricate solar cells, which fall outside the scope of this review. Further information can be found in available literature.^[^ ^278^, ^282^ ^−^ ^285^ ^]^

Nevertheless, a small number of reports cover the use of ball‐milled inorganic perovskite for single‐source vapor deposition of thin films for solar cells. For example, Ajjouri et al. fabricated fully inorganic CsPbX_3_ thin films using fast single‐source evaporation.^[^ ^286^ ^]^ The resulting films, whether used as‐deposited or after annealing, exhibited superior quality compared to those prepared from multi‐source evaporation. Two years later, the same group reported the deposition of CsPbI_2_Br films through single‐source evaporation at 750 °C for 1 min.^[^ ^287^ ^]^ The resulting 700 nm thick film showed a PCE of 8.3% when integrated into solar cells without annealing and a PCE of 10.0% when annealed at 150 °C for 5 min.

Leupold et al. showed a ball milling method in order to prepare perovskite powders. Reactants FAI, MAI, PbI_2_, and PbBr_2_ were added in a ZrO_2_ milling jar at desired stoichiometric ratios. They used 10 mm diameter ZrO_2_ milling balls and kept the ball‐to‐powder ratio as 10:1 in the jar. 11 mL of cyclohexane was added as a milling agent into the 80 mL jar. Ball milling was performed in a Fritsch “Pulverisette 5/4” planetary ball mill at 400 rpm for 5 minutes, followed by a 20 minutes cooling time. This process was repeated as needed to achieve the desired milling time.

Significant efforts were dedicated to developing mechanosynthetic methods for the formation of inorganic perovskites by ball milling. Potassium halides (KX; X = I, Br, or Cl) were incorporated as partial replacements of CsBr in the mechano‐synthesis of CsPbBr_3_ by Ajjouri et al. The partial substitution of both the cation (K) and halogen (I, Br, or Cl) led to the formation of mixed Cs_1‐x_K_x_PbBr_3‐y_X_y_ perovskites, which exhibited longer carrier lifetimes, possibly due to the formation of a passivating non‐perovskite KPb_2_X_5_ layer.^[^ ^288^ ^]^ Solvent‐free mechano‐chemical ball milling was further reported by Hong et al. for the fabrication of phase‐pure, air‐sensitive cubic CsSnCl_3_, cubic CsPbI_3_, and trigonal FAPbI_3_ at ambient temperature and pressure without any post‐synthetic processing.^[^ ^288^ ^]^ Lead‐free, all‐inorganic photodetectors were fabricated using CsSnBr_1.5_Cl_1.5_, which showed a tenfold difference between on‐off currents. Tsvetkov et al. reported that even trace amounts of water can significantly speed up the formation of CsPbI_3_ from the mixture CsI and PbI_2_ through increased mobility of the constituting ionic species.^[^ ^289^ ^]^ This finding highlights the importance of controlling moisture levels during the process and helps explain the wide discrepancies in reaction times necessary for obtaining single‐phase APbX_3_ perovskites and the activation energies of ionic diffusion of the same. According to a study by Aleksanyan et al., Nd doping was more efficient in stabilizing CsPbX_3_ perovskite than partial replacement of I by Br, as monitored through time‐dependent absorption spectrometry.^[^ ^290^ ^]^ In addition, the fabrication of CsPbX_3_ quantum dots was reported through mechano‐chemically grinding/milling, yielding luminescent multicomponent co‐crystals while avoiding the routine use of toxic organic and inorganic solvents, high reaction temperatures, and inert gas protection.^[^ ^291^ ^]^

To improve traditional opaque ball milling equipment, which hinders real‐time reaction progress monitoring, Xiao et al. recently developed a time‐lapsed in situ spectrometer technique, which enabled real‐time observation of the impact of varying the compositions on the absorption properties and quantum yield of perovskite during ball milling. Furthermore, this technology helps to understand the mechanism of mechanosynthesis of perovskite and accelerates the speed of formulation optimization.^[^ ^292^ ^]^

### 5.2 Vertical Bridgman Growth

This section provides a brief introduction to the Vertical Bridgman Growth (VBG) as a solvent‐free method for the fabrication of bulk MHP single crystals. While, to our knowledge, not reported to date, VBG might provide an alternative pathway to synthesize MHPs as source material for further solvent‐free processing and thin‐film deposition. Further information on VBG can be found in the available literature.^[^ ^293^ ^]^

**Figure 28:**
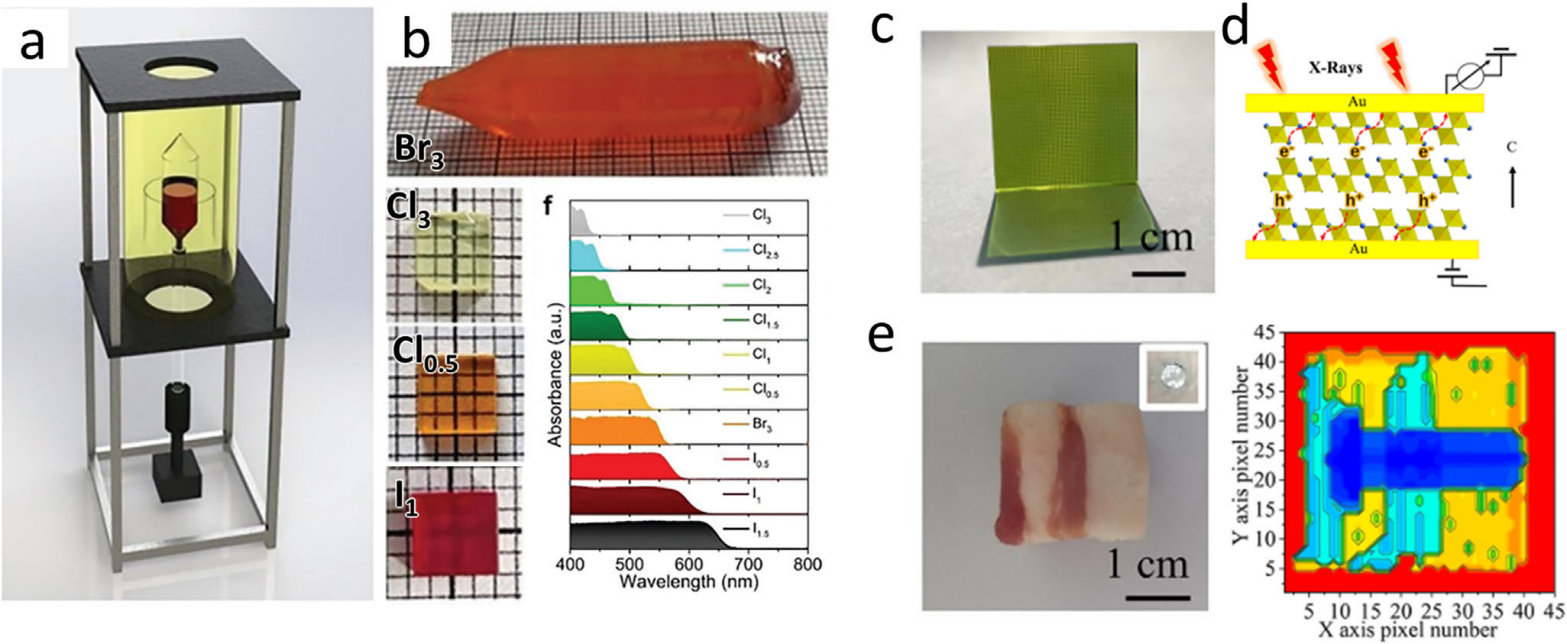
a) Depiction of Bridgman furnace with movable, molt‐filled crucible. b) Photographs of color‐tunable CsPbBr_3‐3n_X_3n_ single crystals, as grown and cut, with corresponding absorption spectra. Reproduced with permission. Copyright 2021, Royal Society of Chemistry.^[^ ^295^ ^]^ c) Up: X‐ray detector array with 45 × 45 pixels based on a Cs_3_Bi_2_Br_9_ single crystal, and schematic of X‐ray detection. Down: photograph of pork phantom tissue with embedded screw and corresponding 40 keV soft X‐ray image. Reproduced with permission. Copyright 2022, American Chemical Society.^[^ ^314^ ^]^

Originally developed by P. W. Bridgman in 1925 to process metals, the VBG method has been adapted to fabricate large cylindrical perovskite single crystals with typical lengths of a few centimeters and around a centimeter in diameter.^[^ ^294^ ^]^ This was achieved by sealing precursors in a cylindrical crucible that was heated in a vertical tubular furnace to create a melt (**Figure** **28**a). Slowly lowering the crucible out of the furnace into ambient air or an oil bath lowers the temperature of the melt and initiates crystallization starting from the bottom. The shape of the crucible is designed to promote the growth of a large single crystal, taking advantage of preferential anisotropic crystal growth. When adapted to grow large perovskite single crystals, this approach offers a few advantages, especially when compared to solvent‐based growth. Precise control over temperature, the spatial‐temporal temperature gradient, and cooling rates, allows for excellent control over the crystal growth process. Furthermore, the absence of solvents eliminates challenges like mismatch of precursor and single crystal composition, poor precursor solubility, and solvent‐induced defects.^[^ ^295^ ^]^ On the other hand, the high temperatures necessary to create the melts in the VBG impose a number of limitations and drawbacks: Precursors must be stable and non‐volatile at these temperatures (e.g., around 600 °C for the popular CsPbBr_3_), which excludes many organic cations used in other processes. Moreover, the crystallization is extremely sensitive to detailed kinematic parameters, and crystallization from melt was described as a highly intricate and non‐isothermal process.^[^ ^296^, ^297^ ^]^ Common defects include halide and cation vacancies and 1–2 µm‐sized inclusions, and vaporization of precursors inside the crucible can distort the crystal stoichiometry.^[^ ^298^ ^−^ ^300^ ^]^ While crystal quality generally benefits from the upward thermal drift of impurities, this also leads to inhomogeneous crystal properties along the growth direction.^[^ ^301^ ^]^ Careful tuning of process parameters and precursor purification benefits crystal quality and improve their optical and electronic properties; however, preparing the highest purity crystals with desirable trap densities down to 10^11^ remains highly challenging.^[^ ^302^, ^303^ ^]^

VBG was used for the creation of lead‐halide perovskites with the F,^[^ ^304^ ^]^ Cl, Br, and I,^[^ ^305^ ^]^ but CsPbBr_3_ is by far the most frequently reported composition. More recently, mixed‐halogen compositions drew much attention due to their tunable bandgaps and reduced vacancy densities (Figure 28b).^[^ ^295^ ^]^ In addition, doping with Li, Ag, and Bi was reported to adjust the bandgap, Fermi levels, and defect densities.^[^ ^306^, ^307^ ^]^

Moreover, VBG allows the exploration of unconventional compositions and phases, such as low‐dimensional layered RP with ultra‐low thermal conductivity or lead‐free 2D Bi‐based perovskites with suppressed ion migration, good shelf lifetimes, and promising performance in X‐ray detection (Figure 28c).^[^ ^308^ ^−^ ^310^ ^]^ Experimental evidence of bandgap‐tunability in Ti(IV)‐based perovskite was demonstrated with VBG‐processed material samples, promising potentially stable and environmentally friendly compounds for optoelectronic applications.^[^ ^311^ ^]^ While the omission of solvents improves sustainability, the high temperatures required by this method offset this positive effect. One current development of melt‐grown perovskites relates to lead‐free compounds at reduced temperatures as low as 142 °C.^[^ ^312^, ^313^ ^]^ This advancement could potentially reduce the energetic footprint and thus the environmental impact, and opens VBG to more volatile and temperature‐sensitive compounds.

With regard to device engineering, VBG offers itself as an easily scalable process that is, however, so far limited by the range of perovskite compositions that can be produced and the difficulty of fabricating thin‐film devices from bulky single crystals for incorporation into devices,^[^ ^295^, ^296^ ^]^ which are all layered structures. CsPb‐halides grown by VBG have found applications in X‐ray and γ‐ray detectors, taking advantage of their high atomic weight, large bandgap, high resistivities, long exciton lifetimes, and ability to withstand high photon fluxes of up to 10^10^.^[^ ^296^, ^301^, ^315^, ^316^ ^]^ Their scintillating properties were further used for γ‐ray and neutron detection.^[^ ^307^, ^317^ ^]^ While these materials seem promising for application in detectors and dosimeters, it is unclear how they will perform under realistic conditions and in the long term, especially since reports generally mention storage rather than operational stability. More recently, bandgap‐adjusted single crystals allowed the fabrication of self‐filtering color‐sensing elements.^[^ ^295^ ^]^ While VBG‐processed perovskites with bandgaps down to 1.9 eV (in CsPbI_3_) have been reported, there are no reports that we are aware of on more complex, multilayered optoelectronic devices such as solar cells or LEDs. This is likely due to the challenges associated with processing large single crystals into thin films, integrating them with charge carrier transport layers, and engineering their interfaces. These processing difficulties are currently limiting the broader application of VBG‐derived perovskite materials in optoelectronic devices.

## 6 Challenges and Future Directions

### 6.1 Comparison of Techniques

**Figure 29:**
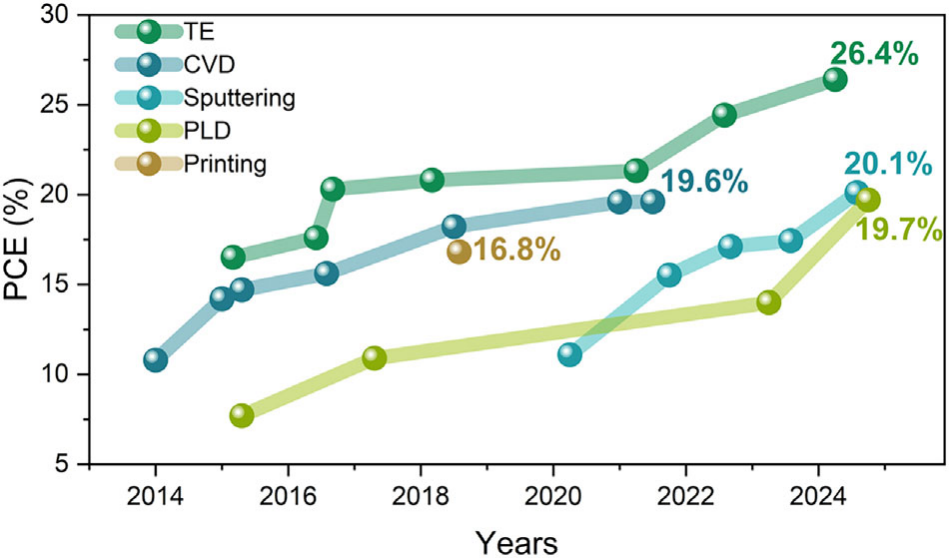
Maximum PCE of solvent‐free PSCs as a function of time.

To illustrate the progress of solvent‐free perovskite thin film deposition methods, we summarize the characteristics of the methods mentioned in this review in **Table** **5**. We also examine the performance evolution of solar cells, the most widely explored perovskite‐based device, as depicted in **Figure** **29**. A clear trend of improvement is evident for each solvent‐free since its first demonstration. Most notably, PSCs fabricated via TE have reached PCEs exceeding 26%,^[^ ^42^ ^]^ rivaling their solution‐processed counterparts.^[^ ^6^ ^]^ While this high‐performance level has yet to be widely reproduced across multiple research groups, potentially due to variations in equipment and processing parameters, these results unequivocally demonstrate that solvents are not essential for obtaining high‐quality perovskite films. Furthermore, upon comparing the state of TE to other methods that would be applied in similar industrial settings, a stark contrast in maturity becomes apparent, which is reflected in the photovoltaic performance as well as the extent of the toolkit available to enhance the process and material properties.

**Table 5:** Characteristics of solvent‐free methods.

Method	Film properties	Post‐treatment requirements	Deposition rate [Å s^−1^]	Stoichiometry control	Additive engineering	Substrate size	Reported device applications	Reported compositions	Refs.
Thermal Evaporation	Smooth, with nanometer‐level roughness, film thickness adjustable from 10 nm to micrometer level, usually without pinholes.	Annealing necessary for sequential TE, optional for one‐step TE.	0.01‐10	Control through relative evaporation rate of independent sources. Organic halide salts are volatile and unstable, complicating rate control.	Additives must be solid, non‐volatile and stable at processing temperatures, e.g. halide salts and some small molecules.	400 cm^2^ device	Laboratory‐scale solar cells demonstrated with PCE up to 26.4%.	Almost all compositions applicable	[25, 37, 42, 45, 46, 71, 72, 75, 115, 132]
Magnetron Sputtering	As‐prepared films prone to buried voids, pin‐holes and large included particles. Post‐treatment necessary.	Deposition of precursors with conversion treatment or direct deposition mostly with vapor or solution post process; recently limited to annealing.	15	Tuned by target composition, high organo‐halide excess might be required.	Halide salts in targets used to modify film properties.	6 mm^2^ devices and 100 cm^2^ films	Laboratory‐scale solar cells demonstrated with PCE up to 20.1%. Demonstrated UV‐ and X‐ray detectors.	MAPbI_3_; FAMAPb(IBr)_3_; CsPbX_3_ X = Br,Cl; Cs_3_Bi_2_I_9_	[138, 139, 141, 142, 143, 146, 148, 149, 150]
Chemical Vapor Deposition	Usually smooth, can be shaped, patterned, or single crystal thin films by adjusting the process	Annealing is necessary for sequential CVD	1–10	Hard to control, as the deposition rate is not monitored.	Additives must be solid, non‐volatile and stable at processing temperatures.	15 cm^2^ device	Laboratory‐scale solar cells demonstrated with PCE up to 19.6%.	Almost all compositions applicable	[161, 162, 166, 169, 170, 172, 180, 200, 201, 202, 203]
Atomic Layer Deposition	Compact, relatively rough films with small grains.	Thermal annealing optional	Deposition carried out in cycles.	In sequential, stoichiometry determined by relative number of ALD steps for CsI and PbI_2_.	–	25 cm^2^ films	–	MAPbI_3_ via solution‐based conversion; CsPbX_n_Y_n‐2_ X,Y = I,Cl,Br; CsSnI3	[206, 207, 208, 209, 210]
Pulsed Laser Deposition	Columnar film growth and homogenous coverage. Medium to small grain size of 50–100 nm. Voids can be included.	Thermal annealing optional	1	Tuned by target composition, high organo‐halide excess required.	Halide salts in targets used to modify film properties.	≈2.5 cm^2^ substrate and 6 mm^2^ device	Laboratory‐scale solar cells demonstrated with PCE up to 19.7%	MAPbI_3_ MAPbBr_3_; MAFAPbI_3_; CsPbBr_3_; CsSnI_3_; Cs_2_AgBiBr_6_	[211, 212, 213, 214, 216]
Electron Beam Evaporation	Compact film, large grains after intense annealing (CsPbBr3).	Thermal annealing	5	Composition of precursor mixture affects film stoichiometry.	–	6 mm^2^ device	Laboratory‐scale solar cells demonstrated with PCE up to 7.61%	CsPbBr3	[220]
Laser Printing	Not uniform, rough, includes non‐perovskite components; vertically phase separated.	–	Multiple printing cycles needed.	Typically yields PbI_2_ ‐rich films due to MAI sublimation.	–	2.25 cm^2^ substrates	Stable under high doses of X‐ray radiation > 200 Gy, blue laser illumination, 90% relative humidity, and thermal stress up to 80 °C for over 300 min in air.	MAPbI_3_	[229, 318]
Flash Evaporation Printing	Low crystallinity, tens nanometers grain size for as‐evaporated films before annealing.	Thermal annealing required	–	Determined by source material.	–	6 mm^2^ and 1cm^2^ device	Laboratory‐scale solar cells demonstrated with PCE up to 16.8%.	MAPbI_3_; CsPbI_2_Br	[233, 236, 237]
Transfer Printing	Higher PLQY compared to the spin coated films. Carrier lifetime and radiative/ nonradiative recombination rates depend on the composition of perovskite.	Post‐transfer annealing at 50 °C for 10 min.	–	Determined by source material.	–	4 mm^2^ device	PeLEDs with EQE 10.5% (680 nm) and 6.7% (493 nm)	CsMAPb(IBr)_3_; CsPb(BrCl)_3_	[243]
Dry Powder Aerosol Deposition	Rough surface, reduced by thermal press. Large agglomerates lead to porous films with voids. The reduction of the number of agglomerates within the powder aerosol is crucial for obtaining working solar cells	Hot‐pressing improves film quality.	Multiple coating iterations necessary.	Determined by source material.	–	6 mm^2^ device	Laboratory‐scale solar cells demonstrated with PCE up to >6% Humidity sensor with a high sensitivity of 21 426.6 pF RH%^−1^, linearity of 0.991, and hysteresis of 1.7%.	MAPbI_3_; CsPb_2_Br_5_; CsPbBr_3_	[252, 253, 257]
Melt‐assisted Growth	Homogenous and compact films with large grains up 1 µm	Pb/MAI bilayer exposed to I_2_ vapor to form reactive melt and trigger perovskite formation.	Sequential process	–	–	600 cm^2^ films and 2.45 cm^2^ devices	Laboratory‐scale solar cells demonstrated with PCE up to 17%	MAPbI_3_	[266, 267, 268]

Among these techniques, TE is closely related to CVD as the source materials undergo similar physical processes during sublimation and film growth. Despite this fundamental similarity, these techniques differ considerably in a number of practical aspects. While TE offers precise control over thickness and deposition timing using quartz crystal monitors and shutters across multiple sources, CVD facilities allow less control over timing and, thus, film composition. Although CVD requires only relatively simple instrumentation and is capable of processing large‐area substrates homogeneously, making it potentially appealing for application on industrial scales, these desirable properties are offset by the relatively low deposition rates. While it seems as if TE will continue to lead the race for high‐throughput solvent‐free fabrication, which is critical for photovoltaic production, CVD is well‐positioned to supply high‐quality films and single crystals for specialized applications.

One notable property common to magnetron sputtering, PLD, and e‐beam evaporation is their industrial relevance driven by, for instance, scalability and potentially short deposition times. What sets them apart from TE are the harsh conditions applied to the source material and potential additives, including high‐power plasma, laser light, and electron beams, respectively. These harsh processes require especially precise control to prevent early degradation of source materials. This is further aggravated since, up to now, these techniques require pre‐synthesized perovskite powders or targets rather than the more robust precursor materials. This inherent single‐source approach imposes an additional drawback related to composition control. The critical importance of stoichiometry is well established both in solution‐processed and TE PSC. In solution processing, the composition is exactly controlled by the weight of the precursors. In TE, the final composition is controlled precisely by the relative rates of independent sources. The single‐source approach common to published PLD, magnetron sputtering, and e‐beam procedures introduces an additional challenge, as film stoichiometry is not easily tuned in situ, but requires the fabrication of a new target or source material. Moreover, the stoichiometric transfer of material from source to sample appears to be particularly difficult in techniques that rely on mediator gas‐related background pressure, i.e., magnetron sputtering and PLD. Available reports address this drawback through highly specialized instrumentation or, more commonly, with a sizeable excess of the organo‐halide component in the source material. While deposition from a single source is an elegant approach, we believe that alternative deposition modes, such as sequential or co‐deposition, should be considered to advance these fields.

### 6.2 Challenges of Solvent‐Free Processing Methods

While many of the solvent‐free processing methods outlined above have demonstrated their potential for fabricating a variety of functional devices based on perovskite materials, there are a number of challenges that hinder their widespread adoption and ability to compete with solution‐based methods. These challenges are shared by many of the aforementioned methods despite their different operational principles and requirements, and include:

1. *Accessibility, expertise, and safety considerations*: Unlike solution processing, which relies for the most part on the readily available spin‐coater, solvent‐free methods often require specialized equipment that is only available in dedicated facilities, is expensive and complex to operate, and has been modified for a specific application. This limits the accessibility of these methods. Additionally, operating these devices necessitates the development of specific handling protocols, often requires a longer training period, and is more time‐consuming than spin coating. For example, while a typical spin coating procedure of a sample might take a few minutes, solvent‐free deposition could require several hours due to the preparation of targets, inks, or powders and the need for vacuum in many deposition techniques. This limits the number of different experiments that can be attempted in a comparable period of time.
  Solvent‐free deposition methods offer inherent safety advantages, especially when considering the potential hazards associated with large‐scale solution processing. While solution processing can be managed effectively in a laboratory setting with gloveboxes and careful handling of small quantities, scaling up these processes for industrial production introduces increased risks due to the larger volumes of hazardous solvents involved. In contrast, solvent‐free methods often involve handling perovskite precursors in bulk form, for example, during mechano‐synthesis or target preparation. While current solvent‐free deposition setups may not always be integrated with gloveboxes, especially for large vacuum systems, this highlights the importance of proactively developing robust safety protocols. These may include the use of personal protective equipment and modifications to deposition facilities to ensure the safety of both operation and maintenance, especially as these techniques mature and transition to industrial scales. Despite these considerations, the relative safety and environmental friendliness of solvent‐free methods, combined with their potential for large‐scale production, underscores their importance for the future of perovskite technology. The fact that a relatively small number of research groups are currently exploring these methods makes their achievements even more remarkable, especially when compared to the extensive research on solution processing. We hope this review inspires more researchers to investigate solvent‐free deposition and contribute to the growing body of knowledge in this promising area.
2. *Difficulty in controlling film stoichiometry*: Precisely controlling film stoichiometry is crucial for achieving the desired perovskite composition and properties. As long as the precursors are soluble in the selected solvents, ink composition can be tuned by adjusting the precursor quantities in the perovskite solution,^[^ ^319^ ^−^ ^321^ ^]^ which is reflected in the stoichiometry of the resultant films. This process becomes far more complicated in the case of solvent‐free methods due to differences in the rates with which the different precursors reach and stick to the substrate and re‐evaporation tendencies. As such, achieving the target stoichiometry may require extensive trial‐and‐error experimentation and careful adjustment of deposition parameters. For example, frequently, highly non‐stoichiometric compositions (typically rich in organic halides) are obtained and extensive post‐treatments are required to achieve the intended perovskite stoichiometry in the deposited layer.^[^ ^139^, ^211^, ^212^, ^214^ ^]^ Considering that for many techniques it is necessary first to form the perovskite powder by mechano‐synthesis and then press them into a target or form a printable toner,^[^ ^146^, ^214^, ^228^ ^]^ the process turns lengthy and labor‐intensive, thus significantly limiting which compositions can be attempted.
  Moreover, the composition of the resultant layer is often impacted by other deposition parameters, such as the rate and the background pressure, making it necessary to test multiple conditions even for the same precursor ratios. Finally, the choice of substrate has also been identified as an important factor, meaning that different preparation methods are necessary for each type of substrate to obtain comparable film stoichiometries. The fast‐evolving pace of the development of novel charge extraction layers makes it necessary to constantly adapt the fabrication procedures to be compatible with the newly discovered, most efficient extraction layers. Experts in certain deposition methods develop some intuition regarding the best way to control the stoichiometry of the coated film, a more systematic approach that elucidates the relationship between precursor ratios and film composition is essential for wider adoption and optimization of these techniques.
  A compelling pathway for addressing this challenge lies in harnessing the power of lab automation tools that are becoming increasingly indispensable across scientific fields. Such tools make it possible to test a wide range of experimental procedures and utilize combinatorial approaches to effectively navigate a wide parameter space. A key advantage of this approach is the fact that the generated data sets can serve as input for machine learning algorithms that are particularly powerful at identifying optimal conditions in a multidimensional parameter space.^[^ ^322^ ^−^ ^324^ ^]^ Such automated approaches can significantly expedite discovery and bring solvent‐free deposited perovskite closer to industrial applications.
3. *Additive engineering*: The strategic incorporation of additives in the solution‐processing of perovskites has proven to be a game‐changing approach. Solution‐processed additives are used for multiple purposes, including controlling the crystallization rate, fine‐tuning the film microstructure, phase, and orientation, preventing the degradation of perovskite precursors, and effectively passivating performance‐limiting defects. In the case of solvent‐free processing, the use of additives remains relatively unexplored. This disparity is likely related to two major factors. First, the compatibility challenges arise from the stringent requirements of solvent‐free processing. For example, liquid additives or additives with low decomposition temperatures are often unsuitable for these methods. Consequently, the design of additives must be meticulously tailored to align with the specific requirements imposed by the solvent‐free deposition methods, resulting in a limited repertoire of suitable options. Secondly, the use of additives further complicates the process of optimization to the standard layer deposition procedure. The presence of additives might require adjustments from the standard deposition conditions since the interaction between the perovskite precursors is altered by the additive's presence. An optimization of the concentration of the additive is also needed. These factors make using additives in solvent‐free deposition methods relatively rare despite the potential benefits they may bring.
  The most promising approach to increasing the use of additives in solvent‐free processing involves focusing on organic ammonium salts, which have proven invaluable in solution‐based techniques. These salts can be added in small amounts to the organic halide precursors (e.g., MAI or FAI) without drastically disrupting the solvent‐free process. This compatibility stems from the similarities between the properties of these additives and the organic halide perovskite precursors, ensuring minimal interference with the deposition process. Concurrently, more efforts are required in the design and synthesis of additives explicitly developed for solvent‐free deposition, taking into account the specific requirements of these methods.
4. *Application in photovoltaic devices*: Despite extensive research efforts of multiple groups, the performance of perovskite devices fabricated by solvent‐free methods still lags behind those processed through solution‐based techniques. In fact, several solvent‐free methods outlined above are yet to yield to functional devices, indicating that further advances are necessary before this application becomes possible. Moreover, even some devices that have been demonstrated still rely on solution processing for specific steps like post‐treatment or deposition of charge extraction layers. While this approach makes it possible to increase device performance, it limits the transferability of the gained knowledge towards industrial applications, where combining multiple deposition methods is impractical. To fully realize the potential of solvent‐free methods, future research needs to prioritize the fabrication of the entire device using solvent‐free methods. To make this possible, it is necessary to develop new charge extraction layer materials and processes that are compatible with solvent‐free deposition. While some existing materials might be easily deposited by such methods (e.g., metal oxides by sputtering or self‐assembled monolayers by TE), others, such as organic polymers, would need to be replaced by suitable alternatives. Much inspiration can be drawn from the field of organic photovoltaics, where solvent‐free processing is well‐established in industrial settings. Research on the deposition of charge extraction layers and metal contacts can also leverage the vast expertise gained from other research fields, like as coatings, electronics, and photonics, which extensively utilize solvent‐free thin‐film deposition methods. We hope that researchers from these fields join the efforts of the perovskite community, and this cross‐disciplinary collaboration will accelerate the advancement of solvent‐free deposition methods for perovskite devices, facilitating their industrial adoption and integration into various applications.
5. *Reproducibility*: Reproducibility remains an inconspicuous challenge in the field of perovskites. Although solution‐processed perovskites also suffer from irreproducibility issues, these can be compensated for by increasing the number of fabricated layers and devices due to the relatively short time that is required for these processes. However, this approach is less feasible for solvent‐free deposition methods, in which sample processing is far more time, resource and labor‐intensive. Another critical factor affecting reproducibility is related to the specific condition of the deposition chamber, which impacts the outcome of the experiments. For example, prior depositions can leave residual precursor materials on the chamber walls, which may inadvertently contaminate subsequent samples, altering their properties despite seemingly identical deposition procedure. Similarly, methods that rely on the preparation of a target might also lead to irreproducibility depending on how often the same target is used for deposition, as the amount of material and its microstructure within the target change with every use as the target material composition and microstructure evolve with each use.
  To address these issues, it is necessary to develop standardized procedures for handling the deposition chambers and implementing reference test protocols that can be used to assess process consistency. For instance, pre‐heating the vacuum chamber walls before use can minimize residual precursor contamination, thus improving process reliability. Detailed documentation and open sharing of experimental data are crucial for fostering knowledge exchange and developing effective mitigation strategies. These measures are essential pre‐requisites for transfer of the deposition processes to industry, where stringent yield requirements demand exceptional reproducibility.
6. *Scalability*: Perovskite photovoltaics are on the brink of widespread industrial adoption. However, the fabrication of PSC at a high throughput that would satisfy economic requirements is elusive to date. Silicon‐perovskite‐tandem cells and flexible perovskite solar modules will likely be among the first large‐area applications. Their industry‐scale fabrication through solvent‐free methods presumes that two distinct challenges can be overcome: High‐quality perovskite needs to be deposited on large substrates or spinning rolls first homogeneously and second rapidly.
  A portion of the techniques discussed here are already conventionally used in large‐area processes, most prominently TE, magnetron sputtering, e‐beam evaporation, CVD, and ALD. We believe that the scientific community should work closely with industrial partners to promote upscaling through the joint development of instrumentation, e.g., appropriately sized linear evaporation sources or sputter targets. While most reports available to date focus on important characteristics such as the performance, stability, or resource efficiency of PSC, only a few specifically emphasize the deposition rate as a target metric. Going forward, we suggest that materials and processes should also be tuned to facilitate fast deposition speeds. Source material will likely be exposed to even harsher conditions, compared to the laboratory setting, when higher ablation rates or vapor pressures are desired. Here, compositional tuning and additives might contribute threefold if applied strategically: First, as already reported, by improving film quality. Second, promoting crystal growth and facilitating faster film formation. Third, stabilize the source materials, prevent their premature degradation, and promote a steady deposition process.

## 7 Conclusions

Solvent‐free deposition methods have been adopted for processing many different types of perovskites, showcasing their potential for industrial scalability and environmentally conscious manufacturing. While significant progress has been made, these processes still face many challenges in achieving the device performance levels currently attained through solution‐based processing. To fully unlock the potential of solvent‐free methods, several areas require further development. Refining crystallization control methods is crucial for improving the quality of perovskite films. Identifying and incorporating compatible additive systems designed to match the specific requirements imposed by the solvent‐free process conditions is essential for further performance improvements. Prioritizing the development of devices that rely entirely on solvent‐free processing may accelerate their integration into industrial manufacturing. Progress can be further expedited by developing a deeper fundamental understanding of the crystallization and growth mechanisms governing solvent‐free processing. Moreover, developing methodologies for defect passivation, interface engineering, and strain management will be crucial for optimizing device performance and stability.

While scalability and sustainability represent the main advantages of solvent‐free methods, their full potential in these areas remains largely untapped. Most reported devices are still small‐scale (typically <1 cm^2^), and comprehensive sustainability assessments, including life cycle analyses, have yet to be performed. Nevertheless, solvent‐free deposition methods remain particularly attractive to the industry and are expected to accelerate the transition of perovskite technologies to real‐life applications.

## Conflict of Interest

The authors declare no conflict of interest.
